# Evaluation of 24-Month Effects of the Close to Home Program on Youth Sexual and Dating Violence Across 22 Communities in California: Protocol for a Quasi-Experimental Cluster-Matched Control Trial

**DOI:** 10.2196/81249

**Published:** 2026-07-15

**Authors:** Emma C Jackson, Sabrina C Boyce, Ricardo Vera Monroy, Holly (Shakya) Baker, Jay G Silverman

**Affiliations:** 1 Community Health Sciences, School of Public Health, University of California, Berkeley, 2121 Berkeley Way #6130, Berkeley, CA, 94704, United States, 1 916-709-1123, 1 510-642-2810; 2 Center on Gender Equity and Health, School of Public Health, University of California, San Diego, La Jolla, United States; 3 Tulane Center on Community-Engaged GBV Research, Celia S. Weatherhead School of Public Health and Tropical Medicine, Tulane University, New Orleans, LA, United States

**Keywords:** research design, matched groups, intimate partner violence, primary prevention, social norms, sexual harassment, violence, adolescents, United States

## Abstract

**Background:**

Sexual violence (SV; any sexual activity where consent is not obtained or freely given) and dating violence (DV; abuse or aggression that occurs in a romantic relationship among youth) are interrelated public health concerns among youth in the United States. Despite its urgency, there is a dearth of comprehensive SV and DV prevention approaches that intervene across multiple social ecology levels (ie, at the individual, interpersonal, community, and societal levels). Therefore, the field of violence prevention has called for the development and evaluation of community-level interventions for prevention. Close to Home (C2H) is a community-driven community mobilization primary prevention program that aims to have community-level effects on reducing SV and DV, as well as improving community connection and social norms related to SV and DV, yet it has never been rigorously evaluated.

**Objective:**

This research study aimed to rigorously evaluate the C2H model at three levels of the social ecology (individual, interpersonal, and community) in 22 diverse communities in California.

**Methods:**

This protocol outlines a quasi-experimental cluster-matched control trial to evaluate the 24-month effects of C2H on SV and DV among youth ages 14‐24 years. The study used a social network (SN) sampling design to assess the diffusion of intervention effects across youth and their SNs, as well as community-wide school-based data from the California Healthy Kids Survey to provide critical evidence on the effectiveness of community mobilization as a viable community-level approach for SV and DV prevention. Primary outcomes included experiencing SV and DV with a 12-month incidence for youth ages 14‐24 years, including sexual assault, in-person and online sexual harassment, and DV. Secondary outcomes included protective social norms rejecting SV and DV and community connection. Further, 11 preselected implementation sites from across California participated in the evaluation and were matched with 11 control program sites based on key demographics and county-level factors related to SV.

**Results:**

This study was funded in September 2020, and institutional review board approval was obtained in February 2021. Baseline survey data were collected on a rolling basis between October 2021 and June 2023 from 953 participants of the SN sample. Follow-up survey data were collected from September 2023 to November 2025 from 746 participants. Data analysis will be conducted from summer to fall 2026. Study findings will be published in late 2026.

**Conclusions:**

This study is among the first to rigorously evaluate a community mobilization approach to SV or DV prevention in the United States. The evidence will support community-level approaches to address the high prevalence of SV and DV in US youth populations, and this study was developed in response to the field’s call for further investigation of prevention strategies that address prevention at the outer levels of the social ecological model (ie, community and societal-level prevention).

## Introduction

Sexual violence (SV; any sexual activity where consent is not obtained or freely given) [1] and dating violence (DV; abuse or aggression that occurs in a romantic relationship) [2] are prevalent and interrelated public health concerns among youth in the United States. Experiencing SV or DV during the developmental period of adolescence has been shown to increase risk for lower educational attainment [3-5], continued experiences of violence [6,7], poor cognitive function, substance use, disordered eating, poor mental health, and suicide [8-10]. Data from the 2023 Youth Risk Behavior Survey, the most recent nationally representative youth sample, show that 11.4% of youth (ages 13 to 18 years) in the United States report past 12-month experiences of SV, 5.9% report past 12-month experiences of sexual DV, and 10.4% report past 12-month experiences of physical DV [11]. Disproportionately high prevalences of SV and DV are experienced by female youth (17% SV, 9.3% sexual DV, and 11.4% physical DV) and sexual and gender minority (LGBTQ+) youth (20.3% SV, 10.7% sexual DV, and 17.6% physical DV) [11]. While both SV and DV are preventable, evidence-based approaches to reducing SV and DV prevalence among youth at the population level are limited.

Primary prevention approaches that aim to prevent SV or DV among youth have primarily focused on individual-level or school-based approaches [12-16]. SV and DV are complex public health problems, and there is a growing consensus that multilevel interventions, including community-level prevention strategies, are necessary [17]. Despite decade-old federal recommendations to target social norms and create protective environments to effectively prevent SV and DV, there is a limited number of community- or societal-level evidence-based SV or DV prevention approaches in the United States [15]. In response to this gap, the field has called for the development and evaluation of community-level interventions to prevent SV and DV [17-19].

Evidence suggests that community mobilization may be a promising strategy for multilevel SV and DV prevention. Community mobilization is a dynamic process of community organizing that mobilizes community members and existing resources to create community-led change [20]. There is evidence of community mobilization’s positive effects on adolescent pregnancy [21], HIV testing and safe sex practices [22,23], gang violence [24], and suicide [25]. Worldwide, particularly in sub-Saharan Africa, multiple community mobilization models designed to reduce SV or DV have shown positive effects [26-28]. These community mobilization strategies are hypothesized to have effected reductions in SV and DV by targeting social norms around the acceptability of violence and gender expectations and strengthening community connection and trust. Given this related evidence, the potential for community mobilization to have an impact on SV and DV in the United States is strong, but evaluation research is needed.

Close to Home (C2H) is a community mobilization SV primary prevention program that mobilizes youth and adults to catalyze community-driven violence prevention through promoting protective social norms and environments. The C2H model was developed to address SV specifically based on the learnings from evidence-based SV and DV community mobilization models shown to be effective in sub-Saharan Africa [26-28]. First developed by grassroots community organizers for implementation in the community of Dorchester, Massachusetts, C2H was chosen in 2008 as a promising violence prevention strategy to be implemented in 3 Massachusetts communities in partnership with the Massachusetts Department of Public Health and in 10 communities in California in partnership with the California Department of Public Health (CDPH). From 2019‐2024, a total of 12 nongovernmental organizations (NGOs) across California received funding to implement C2H in their community, presenting a unique opportunity for evaluating the effect of community mobilization on SV and DV in the United States.

With funding from the Centers for Disease Control and Prevention (CDC) and in partnership with CDPH’s SV prevention program, we conducted a quasi-experimental, parallel group, cluster-matched control trial of C2H across 22 communities from 2020 to 2026. A priori, primary outcomes included experiences of SV and DV, and secondary outcomes of social norms change related to SV and DV and community connection. The evaluation was designed to assess effects at the individual, interpersonal, and community level by using a social network (SN) sampling design to assess diffusion of intervention effects across youth participants of the program and their SNs, as well as community-wide school-based data.

## Methods

### Ethical Considerations

Ethics approval was obtained from the University of California (UC), San Diego Human Research Protections Program (HRPP) under protocol #201920. Subsequently, UC Davis (1797611-2, reliance agreement 201920S) and UC Berkeley (SMART IRB 201920) approved to rely upon the UC San Diego HRPP approval. This study’s protocol was granted a waiver for parental consent for participation in the SN data collection, and informed consent or assent was obtained from all youth and young adult SN participants. Informed opt-out parental consent and youth assent were obtained for the California Healthy Kids Survey data collection. No later than 3 years after data collection is complete, we will put a completely deidentified data set on an appropriate data archive platform for sharing purposes. Participating pilot testers, egos, and nominated alters 1 and 2 provided assent for participation and were compensated either US $20 (ego and alter 1) or US $25 (alter 2) for participating in the survey and US $10 for each nominated peer who opted in to learn more about the survey. We obtained ethical approval with a waiver of parental consent from HRPP.

### Setting

**Figure 1.**
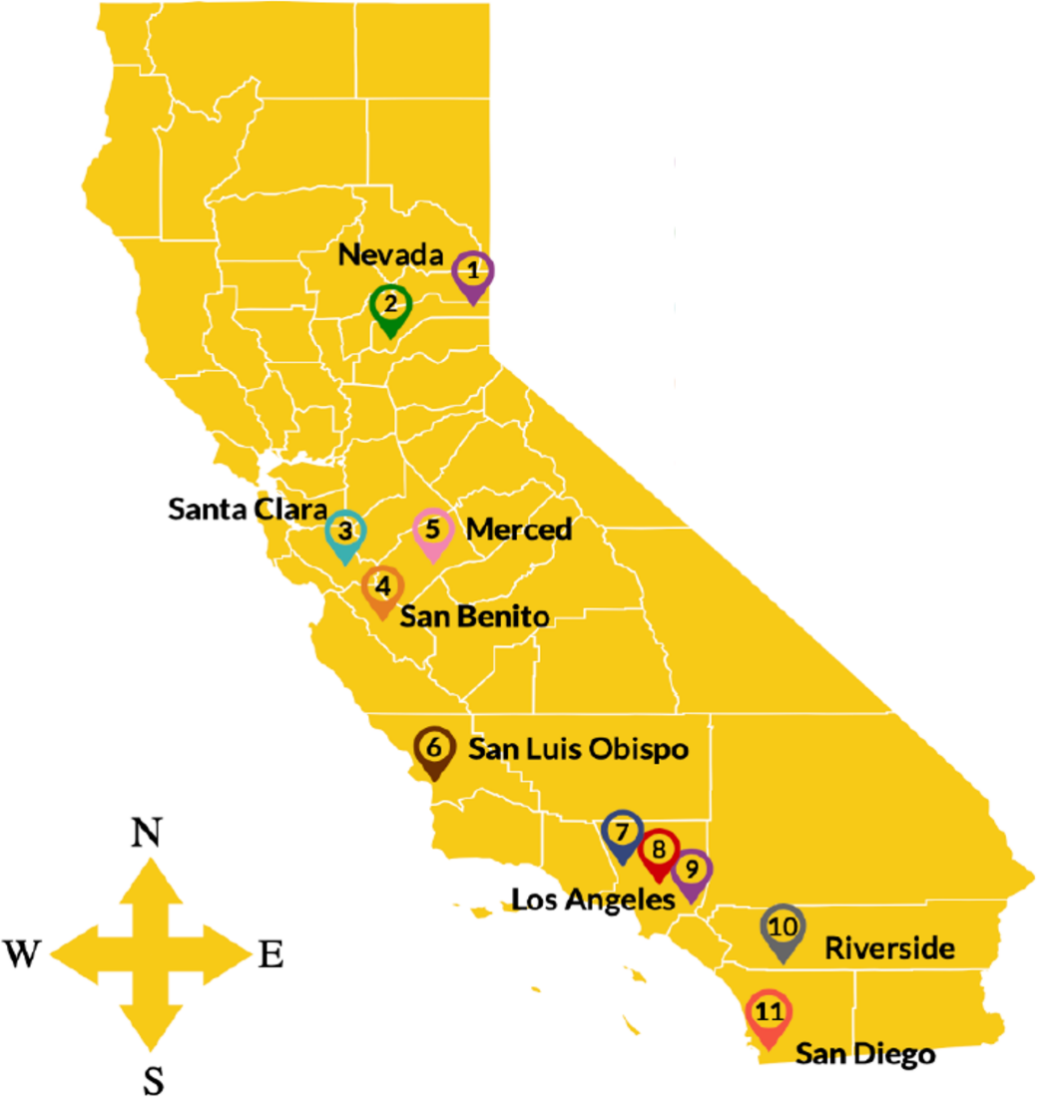
Map of Close to Home intervention sites.

California is a large and diverse state, with SV and DV prevalences that are similar to national prevalences [29,30]. Since 2010, with funding from CDC, CDPH has supported and monitored the statewide implementation of C2H as part of CDPH’s Rape Prevention and Education (RPE) Program and has developed extensive state-level training and technical assistance (TTA) infrastructure, as well as standardized programmatic and evaluation materials to support high-fidelity implementation of the C2H model. In 2018, CDPH’s RPE Program held an open request for funding proposals and selected 9 NGOs for funding to implement C2H over a 5-year grant period from 2019 to 2024. Funded organizations all prioritized populations that are disproportionately impacted by SV, including non-English speaking populations, rural communities, and gender and sexual minorities. A total of 8 of the 9 CDPH-funded sites agreed to participate in the evaluation, and 1 site was excluded from eligibility due to incomplete adherence to the C2H model. Between 2021 and 2022, a total of 3 additional implementation sites received other external grant funding to implement C2H, 2 of which were organizations funded by CDPH to implement C2H in different geographical communities, and agreed to participate in the evaluation. In total, 11 California communities implementing C2H projects were selected a priori as intervention sites for this evaluation (Figure 1).

### Intervention Sites

Each of the 11 C2H intervention sites defined the geography of the implementation community by a target zip code and a high school within the target zip code. Of the 11 implementation communities, 8 communities served predominantly non-English speaking populations, 8 communities served communities where the majority of the residents were marginalized racial or ethnic minorities, 3 communities served rural communities, 6 communities served communities with incomes below the federal poverty line, and 2 communities focused on serving gender and sexual minorities. Participating communities were located across northern, central, and southern regions of California (Figure 1). Each site began a new cycle of C2H implementation between mid-2021 and 2022, after baseline data collection.

### Intervention Description

The C2H model provides a framework and process for mobilizing community members to engage in community-led SV prevention. After recruiting teams of youth and adult community members and training them on root causes of SV and DV and skills for community organizing, the teams (henceforth, “the network”) move through four phases: assess, talk, build, and act. In the assess phase, the network uses a community mapping tool to determine the driving factors of SV in the community and to formulate learning questions that the network intends to answer. In the talk phase, the network engages additional community members in conversations about SV through small group community dialogues (called “kitchen table talks”), art and storytelling, and attending public meetings, including community coalition meetings and community meetings with local civic leaders. The purpose of these discussions is to break the silence around SV by sharing stories and experiences to form a shared, public analysis of SV and why it happens, to create energy for community change, and to expand the network by recruiting additional community members to join in a shared vision for change. In the build phase, the network forms project teams to develop plans, develop skills, and gather resources for designing community actions and campaigns to prevent SV based on the vision developed in the talk phase. In the act phase, the work of the previous phases culminates in network members leading community actions and campaigns designed by the network members to reduce SV. These public actions and campaigns were intended to attract even more community members to the network to grow its momentum toward community-level change. Cyclical in nature, the C2H process may then repeat with the expanded network of community members. Participants could withdraw from the program at any time.

**Figure 2.**
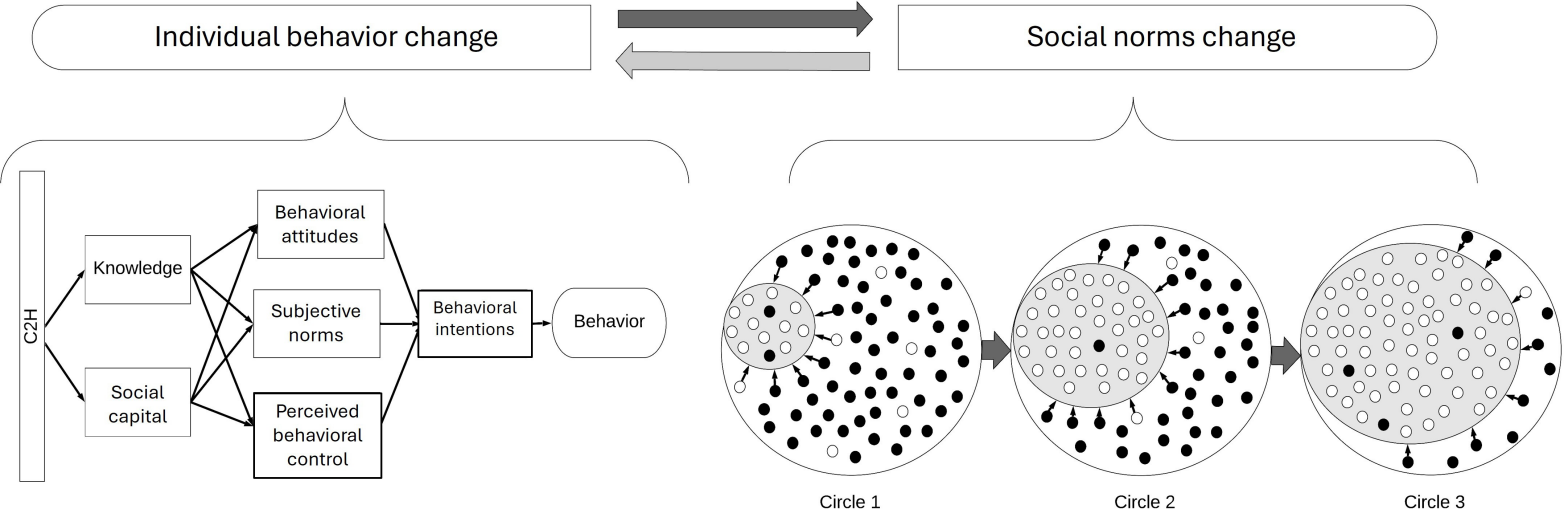
Close to Home theory of change. C2H: Close to Home.

C2H was hypothesized to create impact at the individual, interpersonal, and community levels based on the Theory of Bounded Normative Influence (BNI) [31,32], a theory of social norms change via SNs that is rooted in the principle of Diffusion of Innovation [33], and the Theory of Planned Behavior (TPB) [34]. Based on TPB, at the individual-level, C2H was hypothesized to increase knowledge about SV and DV and social capital, then shift behavioral attitudes, perceived social expectations regarding SV and DV (ie, subjective norms) within the C2H network, and perceived behavioral control. These changes, in turn, influence behavioral intentions and subsequently, behavior to be more protective against SV and DV. At the interpersonal and community levels, based on BNI, C2H was hypothesized to change SV and DV social norms and increase community connectivity to prevent SV through social diffusion, as illustrated in Figure 2. Within a given C2H network, a subset of the local community population, we expected that most individual network members would shift their individual-level beliefs and attitudes to be more protective against SV (based on TPB), despite community members outside of the network possibly holding other beliefs or attitudes (right side of Figure 2, circle 1, white dots). As the C2H community-led actions were implemented across the community, community members outside the network who are reached by these actions would increase their knowledge about SV and DV, shift their SV and DV–related attitudes to be more similar to those of the C2H network, and will develop authentic connections with members of the expanding C2H network, as a result of interacting with network members and their C2H actions. Those community members reached by the C2H actions would become an increasingly larger subset of the community population as more C2H actions are implemented. “Reached” community members would, in turn, influence the knowledge, attitudes, and perceived subjective norms of other community members they had preexisting relationships with to be more protective against SV and DV (circle 2) until the subpopulation holding these protective SV and DV attitudes, previously a minority group in the larger community, becomes a majority within the broader community (circle 3). Through the ongoing, active efforts of C2H participants to increase knowledge, shift attitudes, set new social expectations (ie, social norms) regarding SV and DV, and build connections across community networks, broader social norms change across the community was expected. Additional details on the C2H model can be found online [35].

### Study Design

#### Overview

To evaluate the C2H program, we implemented a quasi-experimental, parallel group evaluation using a cluster-matched control design with 24-month follow-up (ClinicalTrials.gov ID#: NCT05206994). Given that intervention sites were preselected before the evaluation, we used propensity score matching to identify 11 control sites, for a total of 22 communities enrolled in the study. An allocation ratio of 1:1 was used. Using an SN sampling design, data collection was conducted with youth program participants and two layers of their SN to assess the diffusion of the intervention effects to socially connected peers within each community. Additionally, school-based data collection of all high school students at the primary high school in each community was conducted via the California Healthy Kids Survey. This research evaluation was conducted in partnership with CDPH, VALORUS (the TTA provider), the 22 community-based organizations, a community research advisory board (RAB) made up of staff from the C2H-implementing NGOs, and a youth research advisory board (YRAB) made up of youth from the intervention and control communities.

#### Control Condition Sites

Given preselection of C2H sites, a natural experiment using control matching, rather than randomization, was the most rigorous design possible. Matching was conducted with the goal of nearing exchangeability [36]. A youth development program called 4-H was selected to be the control because it (1) recruits teams of prosocial youth interested in helping their community who are supported by adult volunteers, much like C2H, (2) operates in nearly every county across California, allowing for enough variability to optimize matching, and (3) was active during the same time as C2H implementation. The 4-H clubs engaged youth in prosocial learning projects (eg, agriculture, computer science, and art), with adult volunteers as allies to those efforts, yet had no programming related to SV or DV. More details about the 4-H program can be found online [37]. Participants could withdraw from the program at any time. Propensity score matching used a complete list of all local 4-H programs with at least 8 registered youth ages 14‐24 years and not located in a county with an agency implementing C2H or receiving CDPH funding for SV prevention. Propensity scores were assigned based on zip code–level characteristics relating to SV or DV: percent Black population, percent Hispanic population, percent completed bachelor’s or graduate school, percent population under the federal poverty line, criminal rape rate per 100,000 people, presence of a higher education institution, and percent rural population. The 4-H club with the highest propensity score matched to each of the C2H sites was screened by a member of the research team via a short phone call with the adult club facilitator. Screening was conducted for the following inclusion criteria: (1) at least 8 registered youth ages 14‐24 years; (2) stability of club over the past 6 months; (3) in a geographic zip code with no rape crisis center implementing C2H; (4) no violence prevention programming or education offered by the 4-H club; and (5) adult club facilitator willingness to engage in this study. Ultimately, in terms of propensity scores, 5 of C2H sites were matched with the best matched 4-H program, 4 with the second, third, or fifth best match, and 3 that proved difficult to match, which ended up with the 30th, 36th, and 45th best match (out of 69 clubs; see the Data Analysis section for details on analytical approach to address baseline differences). The 4-H clubs with higher propensity scores declined to participate or were ineligible due to an unstable club membership or leadership (discovered upon discussing with the club leader), concerns about parent perceptions of the topic of this study, or being unresponsive to recruitment efforts. Ultimately, the way self-selection played a role in the final control sample likely improved exchangeability between intervention and control communities in that all of the intervention C2H groups had stable club membership and leadership, had low parental concerns about the topic of this study, and had responsive leaders to recruitment efforts.

#### RAB Engagement

A provider RAB and a YRAB were recruited and engaged to inform the development of study protocols, survey instruments, study implementation, as well as ensure the feasibility and acceptability of this study. RAB and YRAB members are compensated for their time. The RAB is made up of key staff from CDPH Injury and Prevention Branch, TTA providers, including the C2H founder and staff from California’s SV coalition (ValorUS), and staff from the 11 C2H local programs. Initially, the RAB met bimonthly to deepen the conceptualization of the theory of change and mechanisms through which C2H was reducing SV and DV in their communities, to guide development of study protocols to ensure coordination and synchrony with C2H implementation, and to provide feedback on outcome and process data collection instruments. Since data collection began, the RAB met on a quarterly basis to provide feedback on implementation and to inform the refinement of study protocols. The YRAB, made up of 7‐10 youth participants of C2H or 4-H programs, engaged in parallel activities throughout the development of study protocols and instruments. The RAB and YRAB will be engaged in the interpretation of results and development of recommendations for future implementation of C2H.

#### Ethics

Research ethical and safety protocols for this study adhered to recommendations issued by the World Health Organization for research on violence against women [38]. The current study protocol and analysis plan are registered on ClinicalTrials.gov (NCT05206994).

#### Data Sources

Three data sources were used in this evaluation: (1) process data from program staff, (2) participant SN data, and (3) data from the California Healthy Kids Survey.

#### Process Data Collection

Process data were collected via monthly surveys with program staff and quarterly consultations with program staff and TTA providers to monitor intervention fidelity. For C2H programs, a phase-specific checklist that reflected the guidelines for implementation of each phase was used to measure fidelity, quality, and frequency of interaction with the target community, C2H participant engagement, and barriers and facilitators to implementation. For control programs, qualitative process data were collected with adult volunteer leaders every 6 months during the intervention period, which included participant-level program dosage estimates, any unanticipated exposure to violence prevention programming, and exogenous changes in the community and schools around SV that may have impacted knowledge, awareness, and/or behavior.

#### SN Primary Data Collection

##### Youth Recruitment

Youth program participants ages 14‐24 years were recruited to this study from each C2H site and the matched control site within 6 months of one another (henceforth, “egos”). Ego recruitment was initiated at a C2H site as soon as the site had recruited and established a youth team to begin a new cycle of C2H implementation, between October 2021 and May 2023. Due to major disruptions caused by the COVID-19 pandemic, C2H implementation began at different time points across sites; 7 sites started in the fall of 2021 and 4 sites started in the fall or winter of 2022‐2023.

To recruit youth, the cofirst author attended a regular C2H/4-H meeting (in-person or virtual), shared this study’s purpose, the SN recruitment process, the risks and benefits of participating, compensation, and, after all questions were answered, invited youth to participate in this study. Those who were interested in participating were sent a personalized link using a provided phone number or email address. Research staff provided tablet devices, device chargers, a Wi-Fi hotspot, and snacks to participants as needed. After this initial meeting, as new youth were recruited to the C2H/4-H groups, research staff continued recruitment as needed, providing virtual or in-person survey orientations as needed to invite all C2H/4-H members to participate and maximize ego recruitment.

Youth accessed the survey via a Qualtrics link, which began with an electronic informed assent or consent form, proceeded to eligibility screening, and concluded with the full survey instrument. Eligibility criteria included being between the ages of 14 and 24 years, currently living in California, being a current participant in a C2H or 4-H group (or for nominated alters, being nominated by a current participant), and providing informed consent or assent. Youth who declined to participate or were not eligible to participate were offered an alternative online activity to do while others were taking the survey to conceal participation. The survey took approximately 40 minutes to complete. At the end of the survey, youth were asked to provide their contact information so that they could receive online incentives and be recontacted for retention check-ins and for the 24-month follow-up survey. Participants received a US $20 online gift card within 24 hours of completing the survey. All survey participants were provided with an online list of resources (eg, SV, sexual harassment and DV advocacy, LGBTQ+ support, and mental health services).

To gather data on SN members (henceforth, “alters”) of the egos, the survey included SN name generator questions to prompt participants to identify up to 10 peers from their SN (ie, “alter 1 s”). Refined via pilot testing, the name generator question prompt was “of the people in your life who live, go to school, or work in or very close to [geographic location of the community site] and are between the ages of 14 and 24…” and the specific name generator questions were “Who do you consider a close friend (including any dating/romantic partners you have)?” “Who are the people that you participate in activities with and who you also consider a friend (activities could include things like sports, jobs, online or in-person meetings, after-school clubs, or other groups involving people around your age)?” and “Besides your close friends, who do you talk to about issues that are important to you?”

Participants were then shown within the survey a list of all unique individuals they nominated, and participants were asked to rank their level of closeness to each peer. For their six highest-ranked nominated peers, participants are asked to answer questions about how each listed peer would respond to scenarios relating to SV behaviors (ego-centric SN data) and provide the peer’s contact information (first name, first two initials of last name, phone number, or email) for recruitment to the SN sample. After participants submitted their peer nominations, the Qualtrics platform was programmed to automatically send survey invitations to the nominated peers via text message or email. At the same time, participants were prompted within the survey to personally inform their nominated peers about this study, with sample text message language provided to support their outreach. This personal outreach was encouraged to help reduce participants’ concerns about nominating peers and to “warm up” peers for the incoming communication from this study’s team. After the initial invitation was sent to nominated peers, they were reminded about the opportunity to participate after 2 days, and contact information was deleted after 14 days.

Once a nominated peer accessed the survey link (on their own device, in a location or time of their choice), they completed the same assent or consent form as egos, followed by a modified eligibility screening that included confirmation of being between the ages of 14 and 24 years, being new to the survey (ie, not having taken it before), and knowing the person that nominated them. Additionally, when a nominated peer accessed the survey link, a US $10 incentive was sent to their ego nominator in acknowledgment of their time and effort to recruit their peer. Once screened, these peers became “alter 1s,” received the same survey, the same opportunity to nominate up to 10 peers (yielding potential “alter 2s”), and the same request for providing their contact information and their nominated peer’s contact information as the egos. Upon completion of a survey by an alter 1, the alter 1 received a US $20 gift card within 24 hours. Alter 2 recruitment and participation mirrored that of alter 1 s except they were not asked to nominate peers or provide peer contact information. Alter 2 s received a US $25 gift card for taking the survey, and alter 1, who nominated them, received US $10 when they accessed the survey link. Alter 1 and alter 2 surveys included an embedded video created by the YRAB explaining this study and the risks and benefits of participation, providing similar information as is provided during the ego survey orientation.

##### Informed Consent and Assent

All study participants were notified of potential risks and risk mitigation strategies via informed assent (participants younger than 18 years) or consent (participants 18 years and older). Voluntary informed assent or consent was obtained from participants electronically via a digital signature before participants began the survey. Participants had the option to print the assent or consent document or receive a copy via email for their records. The informed assent or consent document contained written details about this study, risks and benefits to participants, contact information for participant concerns with this study, and the contact information of the principal investigator (PI) and approving ethics board (see Multimedia Appendix 1: informed consent and assent templates). The research staff who conducted the survey orientations verbally explained important details from the informed assent or consent form and gave the opportunity for attendees to ask questions at any point before, during, or after survey administration.

A waiver of parental permission for participants aged younger than 18 years was requested because the research involves no more than minimal risk to the participants, and the waiver does not adversely affect the rights and welfare of the participants. This waiver was approved by the UC San Diego ethics board (protocol #201920). While parental permission was waived due to the minimal risk for participants, parents of C2H and 4-H participants aged younger than 18 years were given the opportunity to refuse to permit their child to participate in this study to comply with research requirements of the California 4-H Youth Development Program and ensure identical recruitment processes for intervention and control youth. This option was provided only to participants (“egos”) in the intervention or control programs because alters were not subject to 4-H requirements. One week before the survey orientation and recruitment meeting, C2H and 4-H youth facilitators emailed parents of all those participants aged younger than 18 years a link to an online opt-out form allowing them to decline their child’s participation in the survey. Parental opt-out remained available until the day of the survey orientation.

##### Risk Mitigation

Given the topic of this study and this study’s population, risk mitigation was a top priority throughout implementation, including in this study’s design, data collection, data management, and data analysis phases. The greatest risk for study participants aged younger than 18 years included loss of confidentiality. To maintain confidentiality, all personally identifying information was immediately collected in a survey form separate from the survey responses and stored in a separate, password-protected file that was saved on a secure server through the UC. In this file, participant names were removed and replaced with a participant ID, and only two people from the research team had access to the contact information file. Participant contact information was maintained in this secure file for the purpose of recontact for follow-up data collection and was deleted as soon as study participation ended. This procedure was described to participants in the consent or assent form, in addition to an assurance that names and other identifying information would never appear in reports of this study’s results.

This study’s risk section of the consent or assent form also outlined risks related to the SN study design, making it clear to participants that if they elected to nominate peers, those peers would know that they had nominated them, but would not know that they had participated in this study.

All participant responses were protected by a certificate of confidentiality issued by the CDC. This information was shared with participants in this study’s risk section of the consent or assent form. Circumstances under which researchers would have to disclose participant information provided as part of this study were outlined in the same consent or assent form section so that participants could make an informed choice about what they chose to disclose. Circumstances in which the certificate would not apply included reporting child abuse, a danger to themselves or others, sexual contact between a minor and an adult, and if a participant requested that their information be shared. PI and HRPP contact information was provided to all participants, should any participant have concerns. No such disclosures occurred during data collection.

Concerning emotional risks related to the survey content on SV and DV, the following content warning was provided in this study’s risk section of the consent or assent forms:

*The survey includes questions on experiences of and exposure to sexual violence and dating violence. You may feel nervousness or discomfort while answering these questions. In case you feel any discomfort, you can decline to answer questions about your experiences with sexual violence and dating violence, or you can withdraw at any time by simply exiting the survey. Choosing not to answer any question or withdrawing from the survey will not result in any penalty.*

Additionally, similar content warnings were provided within the survey before participants viewed sensitive questions. Survey content warnings also reminded participants that they were free to skip questions or terminate their participation at any time without penalty.

All study participants were provided with resources related to the survey topic following study participation. Resources were provided to participants within the survey module, and included the RAINN (Rape, Abuse & Incest National Network) National Sexual Assault Hotline, a RAINN information page for LGBTQ+ survivors of SV, the Trevor Project helpline, and Forge Forward’s information page for transgender survivors of violence.

Study protocol breaches, breakdowns of the consent or assent process, violations of participant confidentiality, participant complaints, and any serious issues or adverse events were to be reported immediately to the PI, who was responsible for filing an incident report with the HRPP within 24 hours. Incident reports would have included a detailed account of the problem, the date of occurrence, the date the event came to the attention of the PI, the impact on the participant, and the corrective action taken. No such events occurred during the course of data collection. All risk mitigation protocols were approved by HRPP.

##### Recontacting and Retention for SN Data Collection

All baseline participants were contacted 6, 12, and 18 months after completion of their baseline survey by research staff, using the contact information they provided. Retention check-ins took 1‐3 minutes to complete and served to verify contact information and to remind participants of the 24-month follow-up survey. To build rapport and connection with participants in this study, retention check-ins were responded to individually by a research staff member in real-time; participants were invited to follow this study’s social media (@YouthConnectStudy) to receive posts related to this study and feel a sense of membership in this study; updates on milestones were shared; and participants were invited to join the study’s YRAB. The YRAB has helped guide these retention strategies and manages the social media account, creating and posting content about implementation of this study, about SV-related topics, and updates about the research, a strategy that has been successful in previous longitudinal studies of California youth [39].

##### Follow-Up SN Data Collection

Implementation of the four C2H phases required a minimum of 12 months. Follow-up data were collected 24-months postbaseline. Follow-up surveys were self-administered via an online link by the youth. We reached out to participants when 24 months had passed since their baseline participation via the provided contact information, updated via retention efforts, and reminded them weekly of the opportunity to participate in follow-up data collection to minimize loss to follow-up. Participants received a US $50 incentive for completing the follow-up survey. See Multimedia Appendix 2: Close to Home evaluation participant timeline.

##### SN Data Statistical Power

We powered the matched cluster evaluation trial to have .80 power to detect a reduction in 12-month incident SV (the primary outcome) at α<.05. We estimated we would have a follow-up sample of 791 youth study participants (baseline sample: n=953, 791/953, estimated 85% retention at follow-up) from the SN data collection across 22 study sites (11 C2H and 11 control; estimated 36 youth per cluster). We assumed that the average baseline 12-month incidence of the primary outcome would remain constant for the control group at follow-up (107/395, 27% past 12 months SV). The minimal detectable effect would therefore be a 32% (.27 × .32) reduction in SV (71/396, 18% for 12 months incidence at follow-up). Reducing SV by 32% is feasible, given that a previous rigorous evaluation of the shifting boundaries SV prevention model detected a 43% and 50% reduction in SV [40,41]. Furthermore, 37.5% (297/791) of the sample identified as a sexual and/or gender minority (ie, LGBTQ+), among whom 42.4% (126/297) report SV. Estimating the minimal detectable effect for this subsample (297/791 of follow-up participants, 37.5%, 14 LGBTQ+ youth per site), and a 42.5% baseline incidence for LGBTQ+ controls, this study was also powered to detect a 33.5% (0.335 × 0.425=0.14; 0.425-0.14=28% for 12 months incidence at follow-up, 83/297) reduction in LGBTQ+ SV experiences.

### California Healthy Kids Survey Data–Community-Level Youth Sample

#### Collecting and Accessing Data From the California Healthy Kids Survey

This study also used the California Healthy Kids Survey (CHKS) as a mechanism for collecting evaluation data. CHKS collects school-climate data from in-school youth attending public high schools across California. Researchers and Local Educational Agencies are able to develop and build custom data collection modules as part of the CHKS system. In summer 2021, the research team developed a custom module focused on SV prevention to be included in CHKS, which was made up of an abbreviated set of measures used in the SN data collection, and WestEd integrated it into their survey system. Before and during the 2021 to 2022 and 2022 to 2023 academic years (timing guided by SN data collection for each C2H or control group) and with guidance from WestEd, the research team conducted school recruitment by contacting school district leaders and school principals from schools in the targeted zip codes of study communities via email and telephone on multiple occasions with a description of the research and incentives for schools (US $300 gift card, fees covered). For schools or districts that responded, we conducted an informational call about the purpose of the module, the risks and benefits to the school, and implementation logistics, including that implementation of the module would be requested again in 2 years. We then coordinated with WestEd to ensure the opted-in schools received the module, and schools scheduled a date for implementation. Voluntary opt-out parental consent that included specific language about the SV module was obtained by providing informed consent to students’ parents via school parent portals or websites before implementation, per WestEd protocols. Schools implemented the CHKS core and optional SV modules during one class period with all 9th- and 11th-grade students, which took about 50 minutes. Informed assent and data were collected via an online survey that was administered by teachers and/or school staff with training and protocols provided by WestEd. Students received no incentives for participation. The survey was anonymous and did not collect identifying information from students. A data use agreement with the research team’s institutions, WestEd and the California Department of Education, guided the secure transfer of data from the relevant school years to the research team, and are stored on a protected and secure server.

#### Follow-Up CHKS Data Collection

All schools that participated in baseline CHKS data collection in 2021‐2022 or 2022‐2023 were recruited for 24-month follow-up CHKS data collection in the academic year that included the 24-month follow-up period for each specific study community. Similar recruitment methods to those used in baseline recruitment were used, as many of the administrators have changed over the two years. Implementation, informed consent, and data transfers under new data use agreements were also conducted in the same way as baseline.

#### CHKS Data Statistical Power

We estimated that we would have a sample of 6 C2H schools and 6 control sites at 24-month follow-up for CHKS administration, with an average of 500 students per school, which would provide data from an estimated 6000 students. We assumed control students would have the same past 12-month incidence of SV at follow-up as what has been observed to date in baseline, which would provide sufficient power to detect a 14.5% reduction in SV (reduced from 13.8%, 414/3000, to 11.4%, 343/3000) in the treatment group. It is reasonable that such a reduction in SV is possible, given previous studies [40,41]. We acknowledged that power calculations may vary depending on actual intercluster correlations and the number of schools.

### Survey Measures for SN and CHKS Data Collection Instruments

#### Overview

All primary and secondary outcome measures for this evaluation are summarized below, with additional details in the Multimedia Appendix 3: measures.

#### Pilot Testing

Instruments for the SN and CHKS data collection and the SN protocol were concurrently pilot tested twice (summer and fall 2021) to ensure feasibility and acceptability. A mix of youth from RPE programs, a 4-H state leadership group, and from high schools in this study’s site communities were recruited for pilot testing (n=24 pilot egos), oversampling LGBTQ+ youth in the second pilot. Recruited pilot testers (ie, pilot egos) took the full SN survey (which contains identical items as the CHKS instrument) online, participated in the SN recruitment process, and then provided feedback on the survey and SN recruitment process during a 60-min talk-back session. In the first pilot test, pilot participants provided feedback on an initial list of name generator questions for SN sampling to help with refinement and consolidation into the final three questions. In the second pilot test, the final name generator questions were tested, as well as the newly developed measures related to LGBTQ+ youth.

Additional key changes made based on pilot testing included adding recorded videos of youth explaining this study to the assent form for alters (because they do not receive an in-person orientation such as egos) and adding the option to return later to provide peers’ contact information. To make inviting peers to participate in a survey easier, we also added a text message template into the survey instructions for participants to use to notify their nominated peers about the survey.

### Primary Outcomes

#### Experiences of SV and DV

SV and DV behaviors were measured by assessing past 12-month and lifetime incidence of virtual or online sexual harassment, in-person sexual harassment and sexual assault, and physical, emotional, and sexual DV (Multimedia Appendix 3: measures). Participants were also asked to indicate if the person who did this to them or who they did this to was a dating or romantic partner. Two items were included for virtual or online sexual harassment; one modified from the Digital Dating Abuse Scale [42] and the other modified from the evaluation of Coaching Boys into Men (CBIM) Victimization Scale [16,40]. Two items were asked for in-person sexual harassment and SV; one modified from the Teen Health and Technology Survey conducted in 2010 to 2011 [41], and the other modified from the CBIM Victimization Scale [16,40]. Two DV items were included, also modified from the CBIM Victimization Scale [16,40]. Measurement of the use of SV by a youth followed the same structure, with inverse prompts and response options. For these items, participants indicate if the behavior was “in-person” or “online.” Items were modified from the CBIM intention to intervene and SV intervention behaviors scales [16,40].

#### LGBTQ+-Targeted Forms of SV and DV

A measure to assess experiences with forms of SV and DV targeting LGBTQ+ youth was developed by the research team. Survey participants who selected an LGBTQ+ sexual and/or gender identity received a module (via skip patterns) that contained items assessing forms of violence targeting LGBTQ+ youth, and those selecting an expansive gender identity (ie, any gender except cisgender boy or girl) received an additional set of items assessing forms of violence targeting gender expansive youth. Items included in these assessments were adapted from two existing scales developed for adults: the Identity Abuse Scale [43] and the Transgender-Specific Intimate Partner Violence Scale [44]. They were reviewed by both practice and research experts on this topic and pilot tested with LGBTQ+ youth.

#### Secondary Outcomes

Secondary outcomes were selected based on our theory of change, including constructs from the guiding theoretical models, TPB and BNI.

Behavioral attitudes related to SV were assessed via a modified version of the Illinois Rape Myth Acceptance Scale, adapted by the evaluation of Green Dot [45,46], consisting of 7 statements related to rape. Previous exploratory and confirmatory factor analyses have demonstrated both reliability (Cronbach α=.94) and construct validity for the Illinois Rape Myth Acceptance Scale in various US settings [45], and preliminary baseline analyses indicated evidence of strong reliability in our sample (Cronbach α=.93). Responses to items were summed and averaged to create a score.

Perceived behavioral control was assessed in the form of confidence in the ability to engage in active bystander behavior. Items for this construct were modified from the intentions to intervene and SV intervention behaviors scales from the evaluation of CBIM [16,40]. Participants were presented with eight SV behaviors and asked, “how confident are you that you could say or do something to stop a friend or another peer who was…” This scale demonstrated evidence of strong reliability in preliminary baseline analyses (Cronbach α=.93).

Behavioral intention to actively intervene to prevent SV was assessed using a modified version of the Intentions to Intervene Scale from the evaluation of CBIM [16,40]. In the modified version for this evaluation, participants were asked how they would react to witnessing four different SV behaviors. Response options include “I would laugh or go along with it,” “I wouldn’t say or do anything,” and “I would say or do something to let them know that I think acting like that is not okay.” Preliminary baseline data indicated evidence of reliability (Cronbach α=.85).

Bystander intervention behaviors were assessed using a modified version of the CBIM SV intervention behavior scale [16,40]. Participants were asked to indicate whether they had witnessed each of eight SV behaviors perpetrated by their peers in the past 12 months and how they responded. This scale demonstrated evidence of reliability in preliminary baseline analyses (Cronbach α=.83).

Social capital, aligned with the construct in the TPB, was assessed in terms of community connectedness and social cohesion because of the way the C2H model aims to build trust, relationships, and collaboration among community members as a mechanism for reducing SV. Community connectedness was measured using four modified items from the Hemingway Measure of Adolescent Connectedness (previously found to be internally reliable at Cronbach α=.73) [47,48]. Participants were asked to identify the community they felt most connected to, and then asked about their connection to this community using two modified items from the Sense of Belonging Scale [49]. Social cohesion was measured using two modified items from the Neighborhood Social Cohesion Scale by Sampson et al [50] and six modified items from the Brief Sense of Community Scale by Peterson et al [51]. These eight items asked about perceived social cohesion in the specific geographic location of the C2H or control community to which the participant was linked. Preliminary baseline data demonstrated reliability (Cronbach α=.88).

Subjective norms related to perceived acceptability of SV and related to perceived likelihood of community members intervening to prevent SV were assessed. These items were developed by the research team based on principles suggested by Cislaghi and Heise [52]. Subjective norms regarding acceptability of SV behaviors were measured via modification of four items of the CBIM Intentions to Intervene Scale [16,40], focused on participants’ self-selected community to which they feel most connected. In preliminary baseline analyses, these items showed evidence of reliability (Cronbach α=.89). Subjective norms regarding perceived likelihood of community members intervening to prevent SV were measured with three additional items that included reference to the geographic community. These items were developed using the Shared Concern Scale by Lippman et al [53] (originally developed for the issue of HIV, with an α of .85). In preliminary baseline analyses, these items showed evidence of reliability (Cronbach α=.92).

### Data Safety and Monitoring Plan

The PI oversaw all data collection procedures, data management systems, and analyses to ensure compliance with all regulatory data safety and monitoring plans, including ethics approval of research protocols, data quality assurance, and safe data storage. At the time of enrollment into this study, individuals were assigned a unique study identifier (a randomly-generated 17-character alphanumeric sequence). Research staff, under the supervision of the PI, maintained a list linking participants’ unique study identifiers with provided contact information (email address and cell phone number) stored as a separate electronic file on an encrypted server that only two members of this study’s team could access and was deleted after follow-up data collection. Data collected at each time point was linked via study identifiers only. SN data collected via Qualtrics was encrypted at all times, including during transit, and was backed up weekly during data collection, and was only accessed by research staff. CHKS data were securely transferred to the research team by WestEd. All computers used to upload, analyze, or store CHKS data did so via university-approved encrypted cloud services and were password-protected. The PI and research staff were responsible for immediately reporting any breaches of protocol, breakdowns in the consent process, violations of confidentiality of the data, complaints by participants, or any serious problems or adverse events to the PI, who was then responsible for filing an incident report with the HRPP within 24 hours. All research staff involved in this study with access to participant data completed Collaborative Institutional Training Initiative training on human participants research in accordance with university policies regarding the protection of human participants.

### Data Analysis

#### Analysis of SN Data

The primary outcomes, SV and DV, were analyzed as binary variables, constructed as lifetime and past 12-month indicators of experiencing that form of violence. Secondary outcomes, most of which are assessed as scales, were continuous variables based on mean scores across included items.

We used baseline SN data to understand the clustering of social norms and behaviors within this study’s population (aim 1). To do this, we have described the network characteristics of the sample and assessed how SV social norms and experiences of SV and DV are associated among nominator-nominee dyads. We used dyadic observations from the baseline data for each pair of socially connected youth (dyad; eg, ego and alter 1, and alter 1 and alter 2) to restructure the dataset. We calculated whether having a peer who adheres to a particular norm or behavior was predictive of corresponding responses from paired versus nonpaired individuals. This was done using logistic regression analysis (outcome: yes or no) using a generalized estimating equation to account for correlation among dyads. We assessed for effect modification of these relationships based on the types of relationships (eg, school or other peer, or family), the mode of communication, gender concordance, and racial concordance. This analysis provided us with pertinent insight into what norms, attitudes, and behaviors were clustered among socially connected people, and across what relationships, creating a map for understanding opportunities for SN-based intervention. This manuscript is in preparation for publication.

Our primary analysis for main effects (aim 2) will be designed to assess the effects of the intervention on the prevalence of past 12-month SV and DV (sexual assault, online and in-person SH, DV, and LGBTQ+-specific SV) at 24-month follow-up. Our secondary analysis for secondary outcomes (aim 2) will be designed to assess the effect of the intervention on SV attitudes, behavioral intentions to intervene to prevent SV, reported intervention behavior, perceived behavioral self-efficacy to prevent SV, social norms of the acceptability of SV, and community connection and cohesion. First, we will conduct descriptive analyses to explore differences at baseline between the intervention and control groups and factors associated with loss-to-follow-up to understand how these factors may contribute to possible bias in our results. Then, we will assess change over time and by group via adjusted hierarchical difference-in-differences regression analyses to account for unmeasured and measured baseline differences between intervention and control groups, controlling for sampling level (ego, alter 1, and alter 2), accounting for clustering by community, and confounders identified using a dialectical acyclic graph [54]. The difference-in-differences approach does not rely on the strength of the match between intervention and control groups, but rather accounts for baseline differences, offsetting the impact of the three poor intervention-control matches that exist in the sample. Furthermore, we will adjust for factors associated with treatment group assignment to further account for baseline differences. In sensitivity analyses, we will use modern difference-in-differences approaches, such as group weighting approaches proposed by Callaway and Sant’Anna [55], to account for recent developments in understanding potential violations of parallel trends. Subanalyses will be conducted to assess for interaction at each sampling level (ego, alter 1, and alter 2), by gender, LGBTQ+ identity, race or ethnicity, and triangulating with process data. Process data effect modification analyses will assess whether implementation quality, program dosage, and characteristics of program sites modify the observed effect of the intervention on primary and secondary outcomes. Sensitivity analyses will be conducted to assess the variability of effects related to potential violations of the parallel trend assumption, low response rates if they unexpectedly occur, and bias due to potential differences in intervention and control response rates. In addition to the intention-to-treat analysis described above, we will conduct an as-treated analysis based on individual-level dosage estimates from process data. Finally, we will conduct a sensitivity analysis to account for loss-to-follow-up using inverse probability of censoring weighting [56] based on baseline data, should significant baseline differences exist between those lost and those retained. All analyses will be complete case analyses.

Additionally, using SN data, we will test for social diffusion of intervention via SNs (aim 2) via multiple methods. To test the effect of social influence on attitudes, norms, and behaviors, we will use the dyadic dataset of nominator-nominee pairs to test for the effect of social contacts’ attitudes, norms, or behaviors at 24-month follow-up, controlling for both of their own attitudes, norms, or behaviors at baseline and demographics related to these outcomes at baseline. We will explore a variety of model specifications during our analyses and conduct diverse robustness checks given the quasi-experimental design. As we will know whether network nominations are reciprocal (ie, if ego A nominated first tier alter A, did first tier alter A nominate ego A in return?), we can evaluate the possibility of omitted variables or confounding events explaining the associations by examining how the type or direction of the social relationship between ego and alter affects the association between ego and alter (using a “network directionality” test) [57]. Additionally, we will also conduct a sensitivity analysis in which we estimate the bias in the association between ego and alter that might be caused by an omitted variable that is correlated with the prevalence of the outcome variable in both ego and alter [58]. This class of omitted variables includes those that explain friendship formation based on a trait (homophily) and confounding environmental factors that may affect the ego and alter relationships.

Network effects likely will not be uniform across subgroups. For this reason, we will conduct a series of subgroup analyses, looking at potential moderators of potential network effects based on what we learn about the SN characteristics from baseline data. By running a series of interaction models on our main effect analyses, we will consider whether diffusion differs depending on gender, LGBTQ+ identity, the strength of the connection, and other potential moderators such as age, ethnicity, experiences of violence, and program exposure.

#### Analyses of California Healthy Kids Survey Data

The CHKS is representative of in-school youth attending public schools within the target communities of the C2H and control programs (86 per every 100 youth, 86%, participates at study site schools). We estimate that we will have 6 intervention and 6 control schools who participate in both baseline and 24-month follow-up, each with 500 students on average. Difference-in-differences hierarchical models will be used to estimate the impact of the C2H program on primary and secondary outcomes, relative to control program communities, controlling for confounders and accounting for clustering at the school level. If statistical power allows, we will assess for modification due to gender, LGBTQ+ identity, race or ethnicity, implementation quality, and program site. Sensitivity analyses will be conducted to assess the variability of effects due to any potential violations of the parallel trend assumption and bias due to potential uneven inclusion across intervention and control schools.

## Results

This study was funded in September 2020 for three years and was given two additional years of funding through a competitive renewal (through September 2025). A no-cost extension was granted through September 2026. Institutional review board approval was granted in February 2021. Baseline survey data were collected on a rolling basis between October 2021 and June 2023 from 953 participants of the SN sample. Baseline data were analyzed in 2025, and results related to the SN structure are expected to be published in the fall of 2026. Follow-up survey data were collected from September 2023 to November 2025 from 746 participants. Main effect analysis will take place from summer to fall 2026. Main effect findings will be published in late 2026 or early 2027.

## Discussion

### Hypothesized Main Results

We hypothesize that this study will yield evidence that suggests the C2H model had modest but significant effects on reducing past 12-month prevalence of SV and DV, including sexual assault, in-person and online sexual harassment, and DV, among this sample of youth at 24-month follow-up. Additionally, we hypothesize that the C2H model will demonstrate effects on increasing community connection and improving social norms related to SV and DV, the secondary outcomes of this evaluation. We also hypothesize that these effects will diffuse across the youth participants’ SNs and school communities. We anticipate that associations between receipt of C2H and these positive outcomes will be strongest when C2H exposure was longer, when C2H implementation completed a full cycle of the program, and among Hispanic or Latino and LGBTQ+ youth. We expect to observe effect modification across these factors because we hypothesize that high-quality, prolonged exposure to the program will have stronger effects, and because the C2H intervention was intentionally implemented to be inclusive of minoritized populations, particularly toward these two demographic groups, given the size of these populations in California, strengthening the potential impact of the model on these populations.

There are limited evidence-based community-level prevention programs for SV or DV in the United States, with most targeting individual or interpersonal-level behavior change rather than community and societal-level change [18]. While not conducted in the United States and not focused on youth, other community mobilization models addressing SV or DV in other cultural settings have been shown to have positive effects for reducing SV and DV and its risk factors [25,26]. For example, the SHARE intervention evaluated in Rakai, Uganda, was demonstrated to reduce physical and sexual DV among adult women [26]. This evaluation study’s results will add to the literature on community mobilization for addressing SV and DV by providing evidence in the United States, a cultural context in which the salience of community is less pronounced, and among youth, a critical population for preventing first incidents of SV and DV.

### Strengths and Limitations

There are a variety of limitations to this study that should be considered with regard to interpreting results. First, random assignment of the intervention was not possible, given preselection by a funder of the intervention sites. Exchangeability between intervention and control groups was strengthened by propensity score matching of control communities and strategic selection of a comparable control program from which to recruit control participants, yet for three control sites, the quality of control group matching was limited. We will use multiple strategies to try to address limits to exchangeability in our results, including the use of a quasi-experimental analytical approach (difference-in-differences) that accounts for baseline differences and covariate adjustment to control for baseline differences between the intervention and control groups. Second, there are multiple ways in which selection bias may affect our results. For example, participants were not randomly selected and instead were eligible to be in the sample based on their choice to sign up for the program (C2H or 4-H). The types of youth who may volunteer to join such a group are likely systematically different from the youth population in California, limiting the generalizability of the findings. While findings may not be generalizable to the state, because this study is being conducted across 22 communities across all regions of the state, it does include a diverse sample of youth and communities, and likely will provide relevant findings for other diverse communities that might consider implementing C2H in the United States. Third, we may observe nonrandom loss to follow-up at the 24-month follow-up. While we have used extensive efforts to keep the sample of youth engaged and retained over this long follow-up period, we will also likely need to reduce the impact of this form of selection bias by using inverse probability of censoring weighting in our analyses. Finally, this study began during the COVID-19 pandemic, with baseline data collection and intervention initiation occurring between 2021 and 2023. While we expect the pandemic to have impacted control and intervention communities in similar ways, our ability to observe diffusion of intervention effects across SNs may be limited due to the way the pandemic may have caused disruptions to the stability of youth’s SNs between baseline and follow-up. Furthermore, implementation of the C2H model was likely disrupted by the “Great Resignation” that occurred between 2021 and 2022 in the aftermath of the COVID-19 pandemic, in which an unprecedented number of US workers quit their jobs, including prevention staff at C2H implementing organizations [59]. We would expect that the Great Resignation would have a greater impact on C2H implementation than 4-H implementation because adults facilitating C2H groups were primarily paid staff, and 4-H facilitators were more often long-term volunteers who often had children in the program. Despite these limitations in this study’s design and external factors that may have impacted implementation of the C2H program, this study uses a rigorous quasi-experimental design complemented with quasi-experimental analytic techniques to account for the real-world, practical challenges of evaluating community-level programs. Moreover, this study is strengthened by its SN sampling design and implementation in 22 diverse communities.

### Dissemination of Results

With these research results, scientific manuscripts focused on baseline, primary, and secondary outcome results will be prepared by the research team and reviewed by all investigators before journal submission, with the aim of publishing at least three manuscripts. Additionally, the RAB and YRAB will play a critical role in shaping and implementing the dissemination and translation plan for this study. The research team will develop practitioner-focused reports of evaluation findings (summary report, 1‐2 briefs) to share with relevant local, state, and national stakeholders, including C2H implementing organizations, California rape crisis centers, CDPH, federal health agencies, and the scientific community. Community and state partners will assist in the dissemination of results via their networks of practitioner audiences. Results will be shared at practitioner-focused and research conferences. Through this dissemination approach, we aim to maximize the impact of this study to generate knowledge on SV prevention and adolescent health in the United States.

Process data will inform the development of a translation manual to guide future replication and scale-up of C2H, if the model is shown to be effective. By triangulating CDPH guidance and TTA support materials and data of real-world implementation of C2H, this manual will identify essential elements of C2H and best practices for implementation in a wide range of diverse communities. The manual will be distributed via practitioner partners. The RAB will host a webinar at the end of this study to share these best practices in implementing and evaluating C2H in diverse communities. Through research publications and practitioner-focused publications, implementation manual, and presentations, results will be disseminated to a wide range of stakeholders to help inform community-level SV programming and adoption of the C2H model across the United States.

### Conclusions

Findings from this evaluation will contribute to the small body of evidence on community-level and social norms-focused SV prevention [18]. In doing so, these results will provide valuable evidence on whether the C2H community mobilization model is effective at reducing SV and DV and enhancing protective factors at the individual, interpersonal, and community level. Furthermore, the SN sampling design will enable further evidence on what characteristics of youth SNs are important for SV and DV risk and to understand the potential ripple effect of the C2H model. Future research on C2H that allows for randomization of the intervention assignment would be useful in deepening our understanding of the effectiveness of this model. Additionally, future rigorous evaluations of other community- and institutional-level SV and DV prevention models are needed to continue growing the evidence on what works to reduce SV and DV at a population level.

## Supplementary material

10.2196/81249 Multimedia Appendix 1 Informed consent and assent templates.

10.2196/81249 Multimedia Appendix 2 Close to Home evaluation participant timeline.

10.2196/81249 Multimedia Appendix 3 Measures data.

10.2196/81249 Checklist 1 SPIRIT checklist for evaluation of the Close to Home (C2H) program protocol.

10.2196/81249 Peer Review Report 1 Peer review report from the Centers for Disease Control and Prevention of the US Department of Health and Human Services.

10.2196/81249 Peer Review Report 2 Peer review report from the Centers for Disease Control and Prevention of the US Department of Health and Human Services

## Abbreviations

BNI: Theory of Bounded Normative Influence
C2H: Close to Home
CBIM: Coaching Boys into Men
CDC: Centers for Disease Control and Prevention
CDPH: California Department of Public Health
CHKS: California Health Kids Survey
DV: dating violence
HRPP: Human Research Protections Program
LGBTQ+: sexual and gender minority
NGO: nongovernmental organization
PI: principal investigator
RAB: research advisory board
RAINN: Rape, Abuse & Incest National Network
RPE: Rape Prevention and Education
SN: social network
SV: sexual violence
TPB: Theory of Planned Behavior
TTA: training and technical assistance
UC: University of California
YRAB: youth research advisory board

## References

[1] Basile KC, Smith SG, Breiding MJ, Black MC, Mahendra RR (2014). Sexual violence surveillance: uniform definitions and recommended data elements, version 2.0. https://stacks.cdc.gov/view/cdc/26326.

[2] Breiding MJ, Basile KC, Smith SG, Black MC, Mahendra RR (2015). Intimate partner violence surveillance: uniform definitions and recommended data elements, version 2.0. https://stacks.cdc.gov/view/cdc/31292.

[3] Henkhaus LE (2022). The lasting consequences of childhood sexual abuse on human capital and economic well-being. Health Econ.

[4] Jordan CE, Combs JL, Smith GT (2014). An exploration of sexual victimization and academic performance among college women. Trauma Violence Abuse.

[5] Pearson J, Wilkinson L (2017). Same-sex sexuality and educational attainment: the pathway to college. J Homosex.

[6] Petit MP, Blais M, Hébert M (2021). Prevalence, co-occurrence, and recurrence of teen dating violence across dimensions of sexual orientation: a longitudinal study using a representative sample of adolescents. Psychol Violence.

[7] Sterzing PR, Gartner RE, Goldbach JT, McGeough BL, Ratliff GA, Johnson KC (2019). Polyvictimization prevalence rates for sexual and gender minority adolescents: breaking down the silos of victimization research. Psychol Violence.

[8] Hawkins MAW, Layman HM, Ganson KT (2021). Adverse childhood events and cognitive function among young adults: prospective results from the national longitudinal study of adolescent to adult health. Child Abuse Negl.

[9] Exner-Cortens D, Eckenrode J, Rothman E (2013). Longitudinal associations between teen dating violence victimization and adverse health outcomes. Pediatrics.

[10] Manchikanti Gómez A (2011). Testing the cycle of violence hypothesis: child abuse and adolescent dating violence as predictors of intimate partner violence in young adulthood. Youth Soc.

[11] (2023). Explore youth risk behavior survey questions - United States. US Centers for Disease Control and Prevention.

[12] Foshee VA, Reyes LM, Agnew-Brune CB (2014). The effects of the evidence-based safe dates dating abuse prevention program on other youth violence outcomes. Prev Sci.

[13] Taylor BG, Stein ND, Mumford EA, Woods D (2013). Shifting boundaries: an experimental evaluation of a dating violence prevention program in middle schools. Prev Sci.

[14] Niolon PH, Vivolo-Kantor AM, Tracy AJ (2019). An RCT of dating matters: effects on teen dating violence and relationship behaviors. Am J Prev Med.

[15] DeGue S, Valle LA, Holt MK, Massetti GM, Matjasko JL, Tharp AT (2014). A systematic review of primary prevention strategies for sexual violence perpetration. Aggress Violent Behav.

[16] Miller E, Tancredi DJ, McCauley HL (2012). “Coaching boys into men”: a cluster-randomized controlled trial of a dating violence prevention program. J Adolesc Health.

[17] Basile KC, DeGue S, Jones K (2016). Sexual violence prevention resource for action: a compilation of the best available evidence. https://www.cdc.gov/violence-prevention/media/pdf/resources-for-action/SV-Prevention-Resource_508.pdf.

[18] DeGue S, Hipp TN, Herbst JH, Jeglic E, Calkins C (2016). Sexual Violence: Evidence Based Policy and Prevention.

[19] DeGue S, Holt MK, Massetti GM, Matjasko JL, Tharp AT, Valle LA (2012). Looking ahead toward community-level strategies to prevent sexual violence. J Womens Health (Larchmt).

[20] Kretzmann JP, McKnight JL (1993). Building communities from the inside out: a path toward finding and mobilizing a community’s assets. https://designcommunitywork.com/wordpress/wp-content/uploads/2023/03/GreenBookIntro-2018.pdf.

[21] Plastino K, Quinlan J, Todd J, Tevendale HD (2017). Stakeholder education and community mobilization garner support for sex education. J Adolesc Health.

[22] Lippman SA, Neilands TB, MacPhail C (2017). Community mobilization for HIV testing uptake: results from a community randomized trial of a theory-based intervention in rural South Africa. J Acquir Immune Defic Syndr.

[23] Lippman SA, Leddy AM, Neilands TB (2018). Village community mobilization is associated with reduced HIV incidence in young South African women participating in the HPTN 068 study cohort. J Int AIDS Soc.

[24] Allison KW, Edmonds T, Wilson K, Pope M, Farrell AD (2011). Connecting youth violence prevention, positive youth development, and community mobilization. Am J Community Psychol.

[25] Wexler L, Rataj S, Ivanich J (2019). Community mobilization for rural suicide prevention: process, learning and behavioral outcomes from Promoting Community Conversations About Research to End Suicide (PC CARES) in Northwest Alaska. Soc Sci Med.

[26] Abramsky T, Devries K, Kiss L (2014). Findings from the SASA! study: a cluster randomized controlled trial to assess the impact of a community mobilization intervention to prevent violence against women and reduce HIV risk in Kampala, Uganda. BMC Med.

[27] Jewkes R, Nduna M, Levin J (2008). Impact of stepping stones on incidence of HIV and HSV-2 and sexual behaviour in rural South Africa: cluster randomised controlled trial. BMJ.

[28] Wagman JA, Gray RH, Campbell JC (2015). Effectiveness of an integrated intimate partner violence and HIV prevention intervention in Rakai, Uganda: analysis of an intervention in an existing cluster randomised cohort. Lancet Glob Health.

[29] (2021). Youth risk behavior survey data. US Centers for Disease Control and Prevention.

[30] (2023). Youth risk behavior survey (YRBS) 2023 standard questionnaire item rationale. https://www.cdc.gov/yrbs/media/pdf/2023/2023_standard_yrbs_item_rationale.pdf.

[31] Kincaid DL (2004). From innovation to social norm: bounded normative influence. J Health Commun.

[32] Storey JD, Kaggwa EB (2009). The influence of changes in fertility related norms on contraceptive use in Egypt, 1995–2005. Population Rev.

[33] Valente TW (1996). Social network thresholds in the diffusion of innovations. Soc Networks.

[34] Ajzen I, Kuhl J, Beckmann J (1985). Action Control.

[35] Close to Home.

[36] Greenland S, Robins JM (2009). Identifiability, exchangeability and confounding revisited. Epidemiol Perspect Innov.

[37] University of california 4-h youth development program. University of California Agricultute and Natural Resources.

[38] Ellsberg M, Heise L (2005). Researching violence against women: practical guidelines for researchers and activists. https://iris.who.int/server/api/core/bitstreams/400cd30e-b0ed-44ff-88cd-7884fda16cf7/content.

[39] Thomas VL, Chavez M, Browne EN, Minnis AM (2020). Instagram as a tool for study engagement and community building among adolescents: a social media pilot study. Digit Health.

[40] Miller E, Jones KA, Ripper L, Paglisotti T, Mulbah P, Abebe KZ (2020). An athletic coach–delivered middle school gender violence prevention program. JAMA Pediatr.

[41] Mitchell KJ, Ybarra ML, Korchmaros JD (2014). Sexual harassment among adolescents of different sexual orientations and gender identities. Child Abuse Negl.

[42] Reed LA, Tolman RM, Ward LM (2017). Gender matters: experiences and consequences of digital dating abuse victimization in adolescent dating relationships. J Adolesc.

[43] Scheer JR, Woulfe JM, Goodman LA (2019). Psychometric validation of the identity abuse scale among LGBTQ individuals. J Community Psychol.

[44] Peitzmeier SM, Wirtz AL, Humes E, Hughto JMW, Cooney E, Reisner SL (2021). The transgender-specific intimate partner violence scale for research and practice: validation in a sample of transgender women. Soc Sci Med.

[45] Payne DL, Lonsway KA, Fitzgerald LF (1999). Rape myth acceptance: exploration of its structure and its measurement using the Illinois Rape Myth Acceptance Scale. J Res Pers.

[46] Coker AL, Cook-Craig PG, Williams CM (2011). Evaluation of Green Dot: an active bystander intervention to reduce sexual violence on college campuses. Violence Against Women.

[47] Karcher MJ, Davis C, Powell B (2002). The effects of developmental mentoring on connectedness and academic achievement. Sch Comm J.

[48] Karcher MJ (2001). The Hemingway: measure of adolescent connectedness--validation studies. https://files.eric.ed.gov/fulltext/ED477969.pdf.

[49] Fujiwara T, Kawachi I (2008). Social capital and health. A study of adult twins in the U.S. Am J Prev Med.

[50] Sampson RJ, Raudenbush SW, Earls F (1997). Neighborhoods and violent crime: a multilevel study of collective efficacy. Science.

[51] Peterson NA, Speer PW, Hughey J (2006). Measuring sense of community: a methodological interpretation of the factor structure debate. J Community Psychol.

[52] Cislaghi B, Heise L (2019). Using social norms theory for health promotion in low-income countries. Health Promot Int.

[53] Lippman SA, Neilands TB, Leslie HH (2016). Development, validation, and performance of a scale to measure community mobilization. Soc Sci Med.

[54] Sauer B, VanderWeele TJ, Velentgas P, Dreyer NA, Nourjah P (2013). Developing A Protocol for Observational Comparative Effectiveness Research: A User’s Guide.

[55] Callaway B, Sant’Anna PHC (2021). Difference-in-differences with multiple time periods. J Econom.

[56] Dong G, Mao L, Huang B (2020). The inverse-probability-of-censoring weighting (IPCW) adjusted win ratio statistic: an unbiased estimator in the presence of independent censoring. J Biopharm Stat.

[57] An W (2016). On the directionality test of peer effects in social networks. Sociol Methods Res.

[58] VanderWeele TJ (2011). Sensitivity analysis for contagion effects in social networks. Sociol Methods Res.

[59] (2024). How the COVID-19 pandemic prompted more people to change jobs. United States Census Bureau.

