# Supplementary material for: Evaluation of 24-Month Effects of the Close to Home Program on Youth Sexual and Dating Violence Across 22 Communities in California: Protocol for a Quasi-Experimental Cluster-Matched Control Trial
Source: JMIR Res Protoc. 2026 Jul 15;15:e81249. doi: 10.2196/81249 (PMC13372292; doi:10.2196/81249)
Supplement: Multimedia Appendix 1 — Informed consent and assent templates. [file resprot-v15-e81249-s001.pdf]

University of California, San Diego
Youth Assent

Project title: Evaluation of the Close to Home Program in California
Principal Investigator: Dr. Jay Silverman, Professor, University of California, San Diego
Phone: (858) 246-0066; **Email:** jgsilverman@health.ucsd.edu

You are being invited to join a study to understand the effectiveness of an intervention in reducing sexual/dating violence and related outcomes in California. The information from the survey will help researchers learn more about how to best reduce sexual violence and dating violence and promote the safety and well-being for all in the community.

If you agree to participate in this study, you will be asked to complete an online survey twice- once right now and again in 2 years. The survey will take you approximately 45 minutes to complete.

At the end of the survey, you will also be asked to recommend up to 10 peers from your social circle, like friends, classmates, neighbors, etc., to participate in the study. We will ask you to send them a text message inviting them to participate in the study and we will also ask for their contact information so that we may send an automated message to them with a link to the survey. As such, the peers you nominate will know that you nominated them for the study, but they will not be informed if you have participated in the study, nor will you find out if they decide to participate in the study.

You will receive $20 for completing the survey, and $10 for each peer that you nominate to take the survey who opts-in to learning more about the survey, and $30 for taking the survey in two years. This incentive will be delivered to your email through a website called Tango Card. If you agree to participating in the study, Tango Card will have access to your email address and will keep it on file for service-related purposes only.

The following list summarizes your rights as a study participant.

- Participating in this research is voluntary- whether or not you join is your decision. You can discuss your decision with others (such as family and friends).
- You can withdraw at any time by simply exiting the survey and informing the research staff. You can also say yes but change your mind later.
- Choosing not to participate or withdrawing will not result in any penalty. For example, your decision will not affect your relationship with UC San Diego or any community-based organization, including any benefits you may normally get.
- You can say no even if the person inviting you is your friend, relative, or colleague. They will not be told whether or not you agree to participate.
- You may ask questions or mention concerns to the researchers before, during, or after participating in the research.
- All your answers will be completely confidential, meaning your name will not be linked to the answers you mark. Your information will be stored as securely as possible.

Protocol #201920 | v3 | Approved: Feb 01, 2022 12:00 AM MST | Expires: Feb 14, 2023

- This research is protected by a Certificate of Confidentiality. Researchers with this Certificate may not disclose or use information or documents that may identify you in any federal, state, or local civil, criminal, administrative, legislative, or other action, suit, or proceeding, or be used as evidence unless you have consented for this use.

Additional, detailed information about this research is provided below.

Why you have been asked to participate, how you were selected, and what is the approximate number of participants in the study?

You were selected to participate in this study because you are between the ages 14-24 years and are a member of a youth group working to improve the community. There will be approximately 2648 youth and young adults ages 14-24 years old who participate in this study.

What will happen to you in this study?

In addition to the information at the beginning of this form, here are some additional details about what will happen to you if you agree to be in this study,

- You will be asked to answer some questions in an online survey about your knowledge and attitudes related to sexual violence and dating violence, connection with the community, willingness and ability to prevent sexual violence and dating violence, and whether you have experienced or committed sexual violence, dating violence, and sexual harassment. You can decline to answer any question. Your responses to these surveys will not be shared with anyone. The researchers will be the only people to know the information about you and the answers you give to the online survey questions. Your responses are protected by a federal Certificate of Confidentiality, which means they cannot be used as evidence in any legal/court proceedings.
- At the end of the survey, you will also be asked to nominate up to 10 peers from your social circle, like friends, classmates, neighbors, etc., to participate in the study. We will ask you to send them a text message inviting them to the study and we will also ask for their contact information so we may send an automated message to them with a link to the survey. This will help your peers to be less likely to consider the automated message from the research team as "spam". These peers will know that you nominated them but they will not be informed if you have participated in the study yourself, nor will you be informed if they decide to participate in the study.
- The information you provide on your peers, including their first name, first two letters of their last name, and phone number or email, will be used by the secure, encrypted data collection platform to generate a message and unique link to recruit each of your nominated peer. Neither staff working for the data collection platform, nor the UCD research team will have access to the name and phone number you provide for each of your nominated peers. Only 2 members of the UCSD research team will have access to these forms of identifiable information for the sole purpose of generating a message and unique link to recruit nominated peers. This information will be deleted from the data management platform after 14 days and not used for any other purposes.
- After this survey, you will receive a text message every six months to update your contact information and in 2 years, you will be asked to take this survey again.

How much time will study participation take?

Protocol #201920 | v3 | Approved: Feb 01, 2022 12:00 AM MST | Expires: Feb 14, 2023

This online survey will take about 45 minutes to complete. The survey you will take in 2 years will be the same length. There is a total time commitment of approximately one hour and 30 minutes over the course of 2 years.

What risks are associated with this study?

Participation in this study may involve some added risks or discomforts. These include the following:

1. The researchers are the only people who are supposed to know information about you and the answers you give to our questions, but there is some possibility that information about you and your answers might be made known to other people. To help make sure this doesn't happen, after we receive your data, we will immediately remove your personally identifying information, like your name or phone number, and put these in a separate, secure file. We will not store your information or answers in a way that can be connected with your name or other identifying information, like a phone number. Instead, your answers will be given an ID number. Personal information (collected for purposes of re-contact only, separately and securely stored, and deleted as soon as your participation has ended) will be immediately de-linked from survey responses and will be kept on a secure server in a password protected file, accessed only by the PI and Research Project Manager at UCSD. Your name or other identifying information will never appear in any reports of the study results. De-identified data will be shared with UC Davis as part of this research study. Research records may be reviewed by the UC San Diego or UC Davis Institutional Review Boards.

2. The survey includes questions on experiences of and exposure to sexual violence and dating violence. You may feel nervousness or discomfort while answering these questions. In case you feel any discomfort, you can decline to answer questions about your experiences with sexual violence and dating violence, or you can withdraw at any time by simply exiting the survey. Choosing not to answer any question or withdrawing from the survey will not result in any penalty.

3. We will not tell anyone what you tell us without your permission unless there is something that could be dangerous to you or someone else. This research is covered by a Certificate of Confidentiality from the Centers for Disease Control and Prevention (CDC). Researchers with this Certificate may not disclose or use information or documents that may identify you in any federal, state, or local civil, criminal, administrative, legislative, or other action, suit, or proceeding, or be used as evidence unless you have consented for this use. For example, if you disclose involvement in any potentially illegal activity, such as violence, in this study, that information cannot be used against you or any third party in any of the above type of legal proceedings. Information or documents protected by this Certificate cannot be disclosed to anyone else who is not connected with the research *unless*:
    a. You choose to report that you are in danger.
        i. If you choose to tell a member of the study team that an adult is or has been hurting you and you are under age 18, we have to report this to the authorities who are responsible for protecting children so they can make sure you are safe. We do not ask the age of any person who has been violent toward you and we do not ask the age of any persons you report being violent toward;
        ii. Regardless of your age, if you choose to tell us that you are a danger to yourself, we also will need to report that to people who can help you. Again, we do not ask questions about this on the survey;
    b. You are under age 18 and you choose to report that you have had or are having sexual contact with someone age 18 or older. In this case, we have to report this to authorities who are

Protocol #201920 | v3 | Approved: Feb 01, 2022 12:00 AM MST | Expires: Feb 14, 2023

responsible for protecting children so they can make sure you are safe. The survey does not include questions on whether or not you have had or are having sexual contact with someone age 18 or older.

c. You are age 18 or older and you choose to report that you have had or are having sexual contact with someone under age 18. In this case, we have to report this to authorities who are responsible for protecting children so they can make sure the person under age 18 is safe. The survey does not include questions on whether or not you have had or are having sexual contact with someone under age 18.
d. You choose to report that someone else is in imminent danger. If you tell us that you or someone else has intention of seriously harming someone else, we have to report this to authorities who can help improve their safety. The survey does not include questions on whether someone you know is in imminent danger.
e. You request for UCSD to share your information.

Your data will be used for scientific research, as allowed by federal regulations protecting research subjects, but will never be used in any way that identifies who you are.

4. We will ask you to nominate up to 10 peers from your social circle for participation in this study. We will reach out to your peers using an automated message with a link to the same survey. However, before we reach out to them, we will ask you to send your peers a text message informing them about the study. This will help us in making sure that your peers don’t consider the automated message from the research team as “spam”. However, this will also mean that your peers will know that you nominated them which may make you feel some nervousness or discomfort. Your peers, though, will not be told whether or not you participated in the study, nor will you be told if they decide to participate in the study. Neither UC Davis nor staff working for the data collection platform will have access to their information. Only 2 members of the UCSD research team will have access to this information (first name, first two letters of last name, email or phone number) used for the sole purpose of contacting your peers to invite them to participate in the study and will be deleted after 14 days.

Because this is a research study, there may also be some unknown risks that are currently unforeseeable. You will be informed of any significant new risks that are identified.

The information collected as part of the research will not be used or distributed for future research studies.

What are the alternatives to participating in this study?
The alternative to participation in this study is to refuse to participate in this study. Declining to participate in this study will not negatively affect you in any way. Your membership or the role in a community youth group affiliated with this study will not be affected in any way if you decline to participate.

What benefits can be reasonably expected?

Protocol #201920 | v3 | Approved: Feb 01, 2022 12:00 AM MST | Expires: Feb 14, 2023

There will be no direct benefit to you for participating in this study. The researchers and community organizations, however, may learn more about how to best reduce sexual violence and dating violence and promote safety and well-being of all in the community. You will receive information about free or low-cost local support services after completion of the survey.

What happens if you change your mind about participating?
Participation in this research is entirely voluntary. If you decide that you no longer wish to continue in this study, you can withdraw by exiting the survey at any time and informing the research staff.

Can you be withdrawn from the study without your assent?
You may be withdrawn from the study if you do not follow the instructions given for completing the survey.

Will you be compensated for participating in this study?
In compensation for your time, you will receive $20 in the form of an electronic gift card for participating in this research. You will also receive $10 in the form of an electronic gift card for each of your nominated peers who opts-in to learning more about the study (up to $100); you will not be told whether or not they decide to participate in the study. You will also receive $30 in the form of an electronic gift card for participating in the follow-up survey which will be conducted in 2 years.

The UC San Diego research team is working with Tango Card to distribute e-gift cards to study participants. If you agree to participate in this study, the research team will share your email with Tango Card. Tango Card will use your email only on these occasions: 1) to send you your gift card when you finish the survey and 2) to send you gift cards when the peers you nominated opt-in to learning more about the study. Tango Card uses your email to fulfill Reward redemption requests, to perform customer service or contact you (if necessary) and to provide aggregated and anonymous reporting to the UC San Diego research team.

If you desire to not receive Rewards through Tango Card (i.e., to "opt out" of future Rewards), you must request this from the research team at UC San Diego using the technical support contact information at the top of the survey. Tango Card has a contractual obligation to customers to fulfill Rewards once ordered.

Tango Card uses personally Identifiable Information (PII) for internal and Service-related purposes only. Tango Card does not rent or sell PII to third parties. To read Tango Card's full Reward Recipient Privacy Notice, you can go to this link.

If you have data subject rights requests, questions, suggestions, or a complaint about the Tango Card Privacy Policy, please email Tango Card at gdpr@tangocard.com.

Are there any costs associated with participating in this study?
There will be no cost to you for participating in this study.

Who can you call if you have questions?

Protocol #201920 | v3 | Approved: Feb 01, 2022 12:00 AM MST | Expires: Feb 14, 2023

If you have questions about this project or if you have a research-related problem, you may contact Dr. Silverman (jgsilverman@health.ucsd.edu) or his research team at 858-246-0066.

You may also call the UC San Diego Human Research Protections Program Office at 858-246-HRPP (858-246-4777) to inquire about your rights as a research subject or to report research-related problems.

By clicking "You agree" below you are indicating that you are at least 14 years old, have read this consent form, and agree to participate in this research study. Please print a copy of this page for your records.

You Agree

You Do Not Agree

Protocol #201920 | v3 | Approved: Feb 01, 2022 12:00 AM MST | Expires: Feb 14, 2023

University of California, San Diego
Youth Assent-Alter 1

Project title: Evaluation of the Close to Home Program in California
Principal Investigator: Dr. Jay Silverman, Professor, University of California, San Diego
Phone: (858) 246-0066; **Email:** jgsilverman@health.ucsd.edu

You are being invited to join a study to understand the effectiveness of an intervention in reducing sexual/dating violence and related outcomes in California. The information from the survey will help researchers learn more about how to best reduce sexual violence and dating violence and promote safety and well-being for all in the community.

If you agree to participate in this study, you will be asked to complete an online survey twice- once right now and again in 2 years. The survey will take you approximately 45 minutes to complete.

At the end of the survey, you will also be asked to recommend up to 10 peers from your social circle, like friends, classmates, neighbors, etc., to participate in the study. We will ask you to send them a text message inviting them to participate in the study and we will also ask for their contact information so that we may send an automated message to them with a link to the survey. As such, the peers you nominate will know that you nominated them for the study, but they will not be informed if you have participated in the study, nor will you find out if they decide to participate in the study.

You will receive $20 for completing the survey, and $10 for each peer that you nominate to take the survey who opts-in to learning more about the survey, and $30 for taking the survey in two years. This incentive will be delivered to your email through a website called Tango Card. If you agree to participating in the study, Tango Card will have access to your email address and will keep it on file for service-related purposes only.

The following list summarizes your rights as a study participant.

- Participating in this research is voluntary- whether or not you join is your decision. You can discuss your decision with others (such as family and friends).
- You can withdraw at any time by simply exiting the survey and informing the research staff. You can also say yes but change your mind later.
- Choosing not to participate or withdrawing will not result in any penalty. For example, your decision will not affect your relationship with UC San Diego or any community-based organization, including any benefits you may normally get.
- You can say no even if the person inviting you is your friend, relative, or colleague. They will not be told whether or not you agree to participate.
- You may ask questions or mention concerns to the researchers before, during, or after participating in the research.
- All your answers will be completely confidential, meaning your name will not be linked to the answers you mark. Your information will be stored as securely as possible.
- This research is protected by a Certificate of Confidentiality. Researchers with this Certificate may not disclose or use information or documents that may identify you in any federal, state, or local

Protocol #201920 | v3 | Approved: Feb 01, 2022 12:00 AM MST | Expires: Feb 14, 2023

civil, criminal, administrative, legislative, or other action, suit, or proceeding, or be used as evidence unless you have consented for this use.

Additional, detailed information about this research is provided below.

Why you have been asked to participate, how you were selected, and what is the approximate number of participants in the study?

You were selected because you were nominated by a peer to participate in this study, and you are between the ages of 14-24 years. There will be approximately 2648 youth and young adults ages 14-24 years old who participate in this study.

What will happen to you in this study?
In addition to the information at the beginning of this form, here are some additional details about what will happen to you if you agree to be in this study,

- You will be asked to answer some questions in an online survey about your knowledge and attitudes related to sexual violence and dating violence, connection with the community, willingness and ability to prevent sexual violence and dating violence, and whether you have experienced or committed sexual violence, dating violence, and sexual harassment. You can decline to answer any question. Your responses to these surveys will not be shared with anyone. The researchers will be the only people to know the information about you and the answers you give to the online survey questions. Your responses are protected by a federal Certificate of Confidentiality, which means they cannot be used as evidence in any legal/court proceedings.
- At the end of the survey, you will also be asked to nominate up to 10 peers from your social circle, like friends, classmates, neighbors, etc., to participate in the study. We will ask you to send them a text message inviting them to the study and we will also ask for their contact information so we may send an automated message to them with a link to the survey. This will help your peers to be less likely to consider the automated message from the research team as "spam". These peers will know that you nominated them but they will not be informed if you have participated in the study yourself, nor will you be informed if they decide to participate in the study.
- The information you provide on your peers, including their first name, first two letters of their last name, and phone number or email, will be used by the secure, encrypted data collection platform to generate a text message and unique link to recruit each of your nominated peer. Neither staff working for the data collection platform, nor the UCD research team will have access to the name and phone number you provide for each of your nominated peers. Only 2 members of the UCSD research team will have access to these forms of identifiable information for the sole purpose of generating a message and unique link to recruit nominated peers. This information will be deleted from the data management platform after 14 days and not used for any other purposes.
- After this survey, you will receive a text message every six months to update your contact information and in 2 years, you will be asked to take this survey again.

How much time will study participation take?
This online survey will take about 45 minutes to complete. The survey you will take in 2 years will be the same length. There is a total time commitment of approximately one hour and 30 minutes over the course of 2 years.

Protocol #201920 | v3 | Approved: Feb 01, 2022 12:00 AM MST | Expires: Feb 14, 2023

What risks are associated with this study?

Participation in this study may involve some added risks or discomforts. These include the following:

1. The researchers are the only people who are supposed to know information about you and the answers you give to our questions, but there is some possibility that information about you and your answers might be made known to other people. To help make sure this doesn't happen, after we receive your data, we will immediately remove your personally identifying information, like your name or phone number, and put these in a separate, secure file. We will not store your information or answers in a way that can be connected with your name or other identifying information, like a phone number. Instead, your answers will be given an ID number. Personal information (collected for purposes of re-contact only, separately and securely stored, and deleted as soon as your participation has ended) will be immediately de-linked from survey responses and will be kept on a secure server in a password protected file, accessed only by the PI and Research Project Manager at UCSD. Your name or other identifying information will never appear in any reports of the study results. De-identified data will be shared with UC Davis as part of this research study. Research records may be reviewed by the UC San Diego or UC Davis Institutional Review Boards.

2. The survey includes questions on experiences of and exposure to sexual violence and dating violence. You may feel nervousness or discomfort while answering these questions. In case you feel any discomfort, you can decline to answer questions about your experiences with sexual violence and dating violence, or you can withdraw at any time by simply exiting the survey. Choosing not to answer any question or withdrawing from the survey will not result in any penalty.

3. We will not tell anyone what you tell us without your permission unless there is something that could be dangerous to you or someone else. This research is covered by a Certificate of Confidentiality from the Centers for Disease Control and Prevention (CDC). Researchers with this Certificate may not disclose or use information or documents that may identify you in any federal, state, or local civil, criminal, administrative, legislative, or other action, suit, or proceeding, or be used as evidence unless you have consented for this use. For example, if you disclose involvement in any potentially illegal activity, such as violence, in this study, that information cannot be used against you or any third party in any of the above type of legal proceedings. Information or documents protected by this Certificate cannot be disclosed to anyone else who is not connected with the research *unless*:
    a. You choose to report that you are in danger.
        i. If you choose to tell a member of the study team that an adult is or has been hurting you and you are under age 18, we have to report this to the authorities who are responsible for protecting children so they can make sure you are safe. We do not ask the age of any person who has been violence toward you and we do not ask the age of any persons you report being violent toward;
        ii. Regardless of your age, if you choose to tell us that you are a danger to yourself, we also will need to report that to people who can help you. Again, we do not ask questions about this on the survey;
    b. You are under age 18 and you choose to report that you have had or are having sexual contact with someone age 18 or older. In this case, we have to report this to authorities who are responsible for protecting children so they can make sure you are safe. The survey does not include questions on whether or not you have had or are having sexual contact with someone age 18 or older.
    c. You are age 18 or older and you choose to report that you have had or are having sexual

Protocol #201920 | v3 | Approved: Feb 01, 2022 12:00 AM MST | Expires: Feb 14, 2023

contact with someone under age 18. In this case, we have to report this to authorities who are responsible for protecting children so they can make sure the person under age 18 is safe. The survey does not include questions on whether or not you have had or are having sexual contact with someone under age 18.

d. You choose to report that someone else is in imminent danger. If you tell us that you or someone else has intention of seriously harming someone else, we have to report this to authorities who can help improve their safety. The survey does not include questions on whether someone you know is in imminent danger.
e. You request for UCSD to share your information.

Your data will be used for scientific research, as allowed by federal regulations protecting research subjects, but will never be used in any way that identifies who you are.

4. We will ask you to nominate up to 10 peers from your social circle for participation in this study. We will reach out to your peers using an automated message with a link to the same survey. However, before we reach out to them, we will ask you to send your peers a text message informing them about the study. This will help us in making sure that your peers don't consider the automated message from the research team as "spam". However, this will also mean that your peers will know that you nominated them which may make you feel some nervousness or discomfort. Your peers, though, will not be told whether or not you participated in the study, nor will you be told if they decide to participate in the study. Neither UC Davis nor staff working for the data collection platform will have access to their information (first name, first two letters of last name, email or phone number) used for the sole purpose of contacting your peers to invite them to participate in the study and will be deleted after 14 days.

Because this is a research study, there may also be some unknown risks that are currently unforeseeable. You will be informed of any significant new risks that are identified.

The information collected as part of the research will not be used or distributed for future research studies.

What are the alternatives to participating in this study?

The alternative to participation in this study is to refuse to participate in this study. Declining to participate in this study will not negatively affect you in any way. Your membership or the role in a community youth group affiliated with this study will not be affected in any way if you decline to participate.

What benefits can be reasonably expected?

There will be no direct benefit to you for participating in this study. The researchers and community organizations, however, may learn more about how to best reduce sexual violence and dating violence and promote safety and well-being of all in the community. You will receive information about free or low-cost local support services after completion of the survey.

What happens if you change your mind about participating?

Participation in this research is entirely voluntary. If you decide that you no longer wish to continue in this study, you can withdraw by exiting the survey at any time and informing the research staff.

Can you be withdrawn from the study without your assent?

You may be withdrawn from the study if you do not follow the instructions given to you for completing the survey.

Will you be compensated for participating in this study?

In compensation for your time, you will receive $20 in the form of an electronic gift card for participating in this research. You will also receive $10 in the form of an electronic gift card for each of your nominated peers who opts-in to learning more about the study (up to $100); you will not be told whether or not they decide to participate in the study. You will also receive $30 in the form of an electronic gift card for participating in the follow-up survey which will be conducted in 2 years.

The UC San Diego research team is working with Tango Card to distribute e-gift cards to study participants. If you agree to participate in this study, the research team will share your email with Tango Card. Tango Card will use your email only on these occasions: 1) to send you your gift card-when you finish the survey and 2) to send you gift cards when the peers you nominated opt-in to learning more about the study. Tango Card uses your email to fulfill Reward redemption requests, to perform customer service or contact you (if necessary) and to provide aggregated and anonymous reporting to the UC San Diego research team.

If you desire to not receive Rewards through Tango Card (i.e., to "opt out" of future Rewards), you must request this from the research team at UC San Diego using the technical support contact information at the top of the survey. Tango Card has a contractual obligation to customers to fulfill Rewards once ordered.

Tango Card uses personally Identifiable Information (PII) for internal and Service-related purposes only. Tango Card does not rent or sell PII to third parties. To read Tango Card's full Reward Recipient Privacy Notice, you can go to this link.

If you have data subject rights requests, questions, suggestions, or a complaint about the Tango Card Privacy Policy, please email Tango Card at gdpr@tangocard.com.

Are there any costs associated with participating in this study?
There will be no cost to you for participating in this study.

Who can you call if you have questions?

If you have questions about this project or if you have a research-related problem, you may contact Dr. Silverman (jgsilverman@health.ucsd.edu) or his research team at 858-246-0066.

Protocol #201920 | v3 | Approved: Feb 01, 2022 12:00 AM MST | Expires: Feb 14, 2023

You may also call the UC San Diego Human Research Protections Program Office at 858-246-HRPP (858-246-4777) to inquire about your rights as a research subject or to report research-related problems.

By clicking “You agree” below you are indicating that you are at least 14 years old, have read this consent form, and agree to participate in this research study. Please print a copy of this page for your records.

You Agree	You Do Not Agree

University of California, San Diego
Youth Assent- Alter 2

Project title: Evaluation of the Close to Home Program in California
Principal Investigator: Dr. Jay Silverman, Professor, University of California, San Diego
Phone: (858) 246-0066l; **Email:** jgsilverman@health.ucsd.edu

You are being invited to join a study to understand the effectiveness of an intervention in reducing sexual/dating violence and related outcomes in California. The information from the survey will help researchers learn more about how to best reduce sexual violence and dating violence and promote safety and well-being for all in the community.

If you agree to participate in this study, you will be asked to complete an online survey twice- once right now and again in 2 years. The survey will take you approximately 45 minutes to complete.

You will receive $25 for completing the survey, and $30 for taking the survey in two years. This incentive will be delivered to your email through a website called Tango Card. If you agree to participating in the study, Tango Card will have access to your email address and will keep it on file for-service-related purposes only.

The following list summarizes your rights as a study participant.

- Participating in this research is voluntary- whether or not you join is your decision. You can discuss your decision with others (such as family and friends).
- You can withdraw at any time by simply exiting the survey and informing the research staff. You can also say yes but change your mind later.
- Choosing not to participate or withdrawing will not result in any penalty. For example, your decision will not affect your relationship with UC San Diego or any community-based organization, including any benefits you may normally get.
- You can say no even if the person inviting you is your friend, relative, or colleague. They will not be told whether or not you agree to participate.
- You may ask questions or mention concerns to the researchers before, during or after participating in the research.
- All your answers will be completely confidential, meaning your name will not be linked to the answers you mark. Your information will be stored as securely as possible.
- This research is protected by a Certificate of Confidentiality. Researchers with this Certificate may not disclose or use information or documents that may identify you in any federal, state, or local civil, criminal, administrative, legislative, or other action, suit, or proceeding, or be used as evidence unless you have consented for this use.

Additional, detailed information about this research is provided below.

Why you have been asked to participate, how you were selected, and what is the approximate number of participants in the study?

Protocol #201920 | v3 | Approved: Feb 01, 2022 12:00 AM MST | Expires: Feb 14, 2023

You were selected because you were nominated by a peer to participate in this study, and you are between the ages of 14-24 years. There will be approximately 2648 youth and young adults ages 14-24 years old who participate in this study.

What will happen to you in this study?
In addition to the information at the beginning of this form, here are some additional details about what will happen to you if you agree to be in this study,

- You will be asked to answer some questions in an online survey about your knowledge and attitudes related to sexual violence and dating violence, connection with the community, willingness and ability to prevent sexual violence and dating violence, and whether you have experienced or committed sexual violence, dating violence, and sexual harassment. You can decline to answer any question. Your responses to these surveys will not be shared with anyone. The researchers will be the only people to know the information about you and the answers you give to the online survey questions. Your responses are protected by a federal Certificate of Confidentiality, which means they cannot be used as evidence in any legal/court proceedings.
- After this survey, you will receive a text message every six months to update your contact information and in 2 years, you will be asked to take this survey again.

How much time will study participation take?
This online survey will take about 45 minutes to complete. The survey you will take in 2 years will be the same length. There is a total time commitment of approximately one hour and 30 minutes over the course of 2 years.

What risks are associated with this study?
Participation in this study may involve some added risks or discomforts. These include the following:

1. The researchers are the only people who are supposed to know information about you and the answers you give to our questions, but there is some possibility that information about you and your answers might be made known to other people. To help make sure this doesn't happen, after we receive your data, we will immediately remove your personally identifying information, like your name or phone number, and put these in a separate, secure file. We will not store your information or answers in a way that can be connected with your name or other identifying information, like a phone number. Instead, your answers will be given an ID number. Personal information (collected for purposes of re-contact only, separately and securely stored, and deleted as soon as your participation has ended) will be immediately de-linked from survey responses and will be kept on a secure server in a password protected file, accessed only by the PI and Research Project Manager at UCSD. Your name or other identifying information will never appear in any reports of the study results. De-identified data will be shared with UC Davis as part of this research study. Research records may be reviewed by the UC San Diego or UC Davis Institutional Review Boards.

2. The survey includes questions on experiences of and exposure to sexual violence and dating violence. You may feel nervousness or discomfort while answering these questions. In case you feel any discomfort, you can decline to answer questions about your experiences with sexual violence and dating violence, or you can withdraw at any time by simply exiting the survey. Choosing not to answer any question or withdrawing from the survey will not result in any penalty.

Protocol #201920 | v3 | Approved: Feb 01, 2022 12:00 AM MST | Expires: Feb 14, 2023

3. We will not tell anyone what you tell us without your permission unless there is something that could be dangerous to you or someone else. This research is covered by a Certificate of Confidentiality from the Centers for Disease Control and Prevention (CDC). Researchers with this Certificate may not disclose or use information or documents that may identify you in any federal, state, or local civil, criminal, administrative, legislative, or other action, suit, or proceeding, or be used as evidence unless you have consented for this use. For example, if you disclose involvement in any potentially illegal activity, such as violence, in this study, that information cannot be used against you or any third party in any of the above type of legal proceedings. Information or documents protected by this Certificate cannot be disclosed to anyone else who is not connected with the research *unless*:
    a. You choose to report that you are in danger.
        i. If you choose to tell a member of the study team that an adult is or has been hurting you and you are under age 18, we have to report this to the authorities who are responsible for protecting children so they can make sure you are safe. We do not ask the age of any person who has been violent toward you and we do not ask the age of any persons you report being violent toward;
        ii. Regardless of your age, if you choose to tell us that you are a danger to yourself, we also will need to report that to people who can help you. Again, we do not ask questions about this on the survey;
    b. You are under age 18 and you choose to report that you have had or are having sexual contact with someone age 18 or older. In this case, we have to report this to authorities who are responsible for protecting children so they can make sure you are safe. The survey does not include questions on whether or not you have had or are having sexual contact with someone age 18 or older.
    c. You are age 18 or older and you choose to report that you have had or are having sexual contact with someone under age 18. In this case, we have to report this to authorities who are responsible for protecting children so they can make sure the person under age 18 is safe. The survey does not include questions on whether or not you have had or are having sexual contact with someone under age 18.
    d. You choose to report that someone else is in imminent danger. If you tell us that you or someone else has intention of seriously harming someone else, we have to report this to authorities who can help improve their safety. The survey does not include questions on whether someone you know is in imminent danger.
    e. You request for UCSD to share your information.

Your data will be used for scientific research, as allowed by federal regulations protecting research subjects, but will never be used in any way that identifies who you are.

Because this is a research study, there may also be some unknown risks that are currently unforeseeable. You will be informed of any significant new risks that are identified.

The information collected as part of the research will not be used or distributed for future research studies.

What are the alternatives to participating in this study?

Protocol #201920 | v3 | Approved: Feb 01, 2022 12:00 AM MST | Expires: Feb 14, 2023

The alternative to participation in this study is to refuse to participate in this study. Declining to participate in this study will not negatively affect you in any way. Your membership or the role in a community youth group affiliated with this study will not be affected in any way if you decline to participate.

What benefits can be reasonably expected?

There will be no direct benefit to you for participating in this study. The researchers and community organizations, however, may learn more about how to best reduce sexual violence and dating violence and promote safety and well-being of all in the community. You will receive information about free or low-cost local support services after completion of the survey.

What happens if you change your mind about participating?
Participation in this research is entirely voluntary. If you decide that you no longer wish to continue in this study, you can withdraw by exiting the survey at any time and informing the research staff.

Can you be withdrawn from the study without your assent?

You may be withdrawn from the study if you do not follow the instructions given for completing the survey.

Will you be compensated for participating in this study?

In compensation for your time, you will receive $25 in the form of an electronic gift card for participating in this research. You will also receive $30 in the form of an electronic gift card for participating in the follow-up survey which will be conducted in 2 years.

The UC San Diego research team is working with Tango Card to distribute e-gift cards to study participants. If you agree to participate in this study, the research team will share your email with Tango Card. Tango Card will use your email only on these occasions: 1) to send you your gift card when you finish the survey and 2) to send you gift cards when the peers you nominated opt-in to learning more about the study. Tango Card uses your email to fulfill Reward redemption requests, to perform customer service or contact you (if necessary) and to provide aggregated and anonymous reporting to the UC San Diego research team.

If you desire to not receive Rewards through Tango Card (i.e., to “opt out” of future Rewards), you must request this from the research team at UC San Diego using the technical support contact information at the top of the survey. Tango Card has a contractual obligation to customers to fulfill Rewards once ordered.

Tango Card uses personally Identifiable Information (PII) for internal and Service-related purposes only. Tango Card does not rent or sell PII to third parties. To read Tango Card’s full Reward Recipient Privacy Notice, you can go to this link.

If you have data subject rights requests, questions, suggestions, or a complaint about the Tango Card Privacy Policy, please email Tango Card at gdpr@tangocard.com.

Protocol #201920 | v3 | Approved: Feb 01, 2022 12:00 AM MST | Expires: Feb 14, 2023

Are there any costs associated with participating in this study?
There will be no cost to you for participating in this study.

Who can you call if you have questions?

If you have questions about this project or if you have a research-related problem, you may contact Dr. Silverman (jgsilverman@health.ucsd.edu) or his research team at 858-246-0066.

You may also call the UC San Diego Human Research Protections Program Office at 858-246-HRPP (858-246-4777) to inquire about your rights as a research subject or to report research-related problems.

By clicking "You agree" below you are indicating that you are at least 14 years old, have read this consent form, and agree to participate in this research study. Please print a copy of this page for your records.

You Agree

You Do Not Agree

UNIVERSITY OF CALIFORNIA | PARENT LETTER

Project Title: Close to Home Program Evaluation

Principal Investigator: Dr. Jay Silverman, Professor, University of California San Diego
Phone: (858) 246-0066; Email: jgsilverman@health.ucsd.edu

California 4-H Contact: Ms. Lynn Schmitt-McQuitty, Statewide 4-H Director
Phone: (530) 750-1334; Email: lschmittmcquitty@ucanr.edu

Local 4-H Facilitator: [Name]; Phone: [Phone number]; Email: [Email address]

What is the purpose of this study?

The purpose of this research study is to understand the effectiveness of a community-based program to reduce and change the acceptability of sexual violence and dating violence and related outcomes in California. The study will help researchers learn more about how to best reduce sexual violence and dating violence and promote the safety and well-being of all in the community.

What is the purpose of this form?

This letter gives you the information you will need to help you decide whether you want your child to be in the study or not. Please read the form carefully. You will only sign and return this form if you **do not agree** to give your child permission to participate in the study.

Your child is being invited to participate in the study because they have participated in 4-H programming. You can contact the researcher to explain anything that you don't understand. You may ask any questions about the research, the possible risks and benefits, your child's rights as a study participant, and anything else that is not clear by contacting the Principal Investigator at the contact information provided above. When all of your questions have been answered, you can decide if you want your child to be in this study or not.

Why is my child being invited to take part in this study?

Your child is a part of 4-H club that has been selected for the study. The 4-H club serves as a comparison program to a sexual/dating violence prevention program in California. Youth ages 14-24 years in selected 4-H clubs in California are asked to participate in this study.

What will happen during this study and how long will it take?

- If your child agrees to be in this study, they will be asked to fill out an online survey, which will take approximately 45 minutes. Your child will be recontacted two years from now and invited to take the same survey.
- The first survey will be completed online during a 4-H meeting. The second survey will be completed solely online.
- The survey will ask your child questions about knowledge and attitudes related to sexual/dating violence, connection with the community, willingness and confidence to prevent sexual/dating violence among peers, and whether they have experienced or committed sexual violence, dating violence, and sexual harassment.

- Your child can choose to decline to answer any question. Their responses will be stored separately from any information that identifies them so no one outside the UC San Diego research team can link them together. Their identity is protected from ever being used in any local, state, or federal legal proceedings, or for any other purposes, related to these data.
- During the survey, your child will also be asked to nominate up to 10 peers whom they feel close to (for example, close friends from school or from outside of school) who are between the ages of 14-24 to take the same survey. Your child will be sent a text message from UC San Diego that they can forward to their peers explaining that they are nominating them to participate in the study. Your child's friends will not know that your child took the survey.

What are the risks of this study?

The survey includes questions on experiences of and exposure to sexual violence and dating violence. Your child may feel nervousness or discomfort while answering these questions. In case your child feels any discomfort, they can decline to answer any questions about their own experiences with sexual and/or dating violence, and they can withdraw at any time by simply exiting the survey. Choosing not to answer any question or withdrawing from the survey will not result in any penalty.

We will not tell anyone what your child reports on the survey unless they report something that could be dangerous to them or someone else. This research is covered by a Certificate of Confidentiality from the Centers for Disease Control and Prevention. The researchers are the only people who are supposed to know information about your child and the answers your child gives to our questions, but there is a possibility that they may share answers with others, or another child may disclose that your child participated in the research.

We will ask your child to nominate up to 10 peers from their social circle to take the same survey. We will reach out to your child's peers using an automated text message with a link to the same survey. However, before we reach out to them, we will ask your child to send their peers a text message informing them about the study. This text message will read as follows and is all that we are asking your child to send:

Hello <NAME>,

Researchers at the University of California, San Diego are conducting a study to understand how to prevent sexual violence in California. I have nominated you to take the survey for this study.

- *The survey takes 45 minutes.*
- *The survey includes some questions about your experiences with sexual and/or dating violence.*
- ***You will receive a $20 e-gift card for completing the survey, plus $10 for each peer you nominate who opts-in to learning more about the survey (up to $100 in e-gift cards).***
- *Your identity and responses will be kept completely confidential.*
- *Participating in this survey will contribute to understanding the way sexual violence can be prevented in California.*

You will also receive an automated text from the researchers at UC San Diego with more information about the survey and a link to take the survey. You are free to participate or decline to participate in the survey. If you decline, there is no penalty.

This will help us in making sure that their peers don't consider the automated text message from the research team as "spam". However, this will also mean that their peers will know that your child nominated them, which may make them feel some nervousness or discomfort. Your child's peers, though, will not be told whether or not your child participated in the study, nor will your child be told if their peers decide to participate in the study. Again, your child can choose to not complete this section or withdraw from the study at any time without penalty.

Because this is a research study, there may also be some unknown risks that are currently unforeseeable. Your child will be informed of any significant new risks that are identified.

What are the limitations to Certificate of Confidentiality?

Information or documents protected by this Certificate cannot be disclosed to anyone else who is not connected with the research *unless*:

1. Your child chooses to report that they are in danger.
    i. Although the survey does not include questions on whether or not they are currently being hurt, if your child chooses to tell a member of the study team that an adult is or has been hurting them and they are under age 18, we have to report this to the authorities who are responsible for protecting children so they can make sure your child is safe;
    ii. Regardless of your child's age, if your child chooses to tell us that they are a danger to themselves, we also will need to report that to people who can help them. Again, we do not ask questions about this on the survey;
2. Your child is under age 18 and they choose to report to the research team that they have had or are having sexual contact with someone age 18 or older. In this case, we have report this to authorities who are responsible for protecting children so they can make sure they are safe. The survey does not ask your child to provide this information.
3. Your child chooses to report to the research team that someone else is in imminent danger. If your child tells us that they or someone else has intention of seriously harming someone else, we have to report this to authorities who can help ensure their safety. The survey does not ask your child to provide this information.

Your child's data will be used for scientific research, as allowed by federal regulations protecting research subjects, but will never be used in any way that identifies who they are.

What are the benefits of this study?

There are no direct benefits to your child participating in this study. The researchers and community organizations, however, may learn more about how to best reduce sexual violence and dating violence and promote safety and well-being of all in the community. Your child will receive information about free or low-cost local support services after completion of the survey.

Will your child be paid for participating?

If they participate in the online survey, they will be sent a $20 e-gift card upon finishing the survey and will be sent additional $10 e-gift cards for each peer they nominate who opts-in to learning about the survey (up to an additional $100 in e-gift cards, up to $120 in total). Your child will be recontacted two years from now and invited to take the same survey. They will receive $30 for completing the second survey.

The gift cards are distributed through your child's email address by Tango Card. Tango Card keeps emails on file for internal and service-related purposes only. Tango Card does not sell Personally Identifiable Information to third parties. You can read Tango Card's Reward Recipient Privacy Notice here.

There will be no costs to you or your child for taking part in this study.

Who will see the information that your child provides?

The information your child provides through the surveys will be kept confidential to the extent permitted by federal and state law. All names and any other information that might identify your child will be removed immediately after your child completes their participation in the study. All data collected through this survey will be stored on password-protected computers and drives, and any printed data will be stored in a locked cabinet, in a locked office, and only the investigator will have access to the data in its entirety. If results of this project are published, your child's identity will not be made public. Other UC San Diego and UC Davis staff will have access to data after potentially identifiable characteristics have been removed, or data as reported in aggregate.

Identifiable information (first name and first two initials of last name, email, and phone number and peers' first name, first two initials of last name, and email or phone number) will be handled as confidentially as possible. However, individuals from UC San Diego and UC Davis who oversee research may access data during audits or other monitoring activities. Research team members are mandated reporters and may learn of information that requires reporting. The identifiable information provided by your child will be stored in a secure, encrypted data collection platform in order to distribute gift cards, generate a text message and unique link to recruit nominated peers, and to recontact your child regarding follow-up data collection. Neither staff working for the data collection platform nor the UCD research team will have access to the names and phone numbers. Only 2 members of the UCSD research team will have access to these forms of identifiable information for the sole purposes of distributing gift cards, generating a text message and unique link to recruit nominated peers, and recontacting your child regarding follow-up data collection. The identifiable information your child provides about themselves will be kept securely until follow-up data collection is complete (2 years after they first take the survey) and then deleted. The identifiable information your child provides about peers will be deleted after 14 days from the data management platform and not used for any other purposes.

Does your child have a choice to be in this study?

Your child's participation in this study is completely voluntary. Your child will not be treated differently if you or they decide to decline participation. Your decision will not result in any loss or benefits to which you or your child are otherwise entitled. Your child can stop at any time during the study without penalty. Your child is free to skip any question they do not wish to answer. You are free to not allow your child to participate in this study without adversely affecting your or your child's relationship with the investigator, University of California, or 4-H Youth Development.

What If I have questions?

If you have questions about this study or the information in this form, please contact Dr. Jay Silverman, jgsilverman@health.ucsd.edu, or his research team at 858-246-0066; or the local 4-H youth program facilitator (name and contact information at the top of this form). If you have questions about your rights or your child's rights as a research participant or you would like to report a concern or complaint about this study, please contact the UC Davis IRB staff at (916) 703-9158 or email to hsirbeducation@ucdavis.edu

How do I refuse permission for my child to participate?

If you **do not agree** to give your child permission to participate in the study, please fill the following **opt-out** form.

Study Opt-Out Form

Please indicate below if you **do not agree** to give your child permission to participate in this survey by filling in your name and signature, the name of your child, and the town/city of your child's 4-H club. If you do not want your child to participate in this study, please inform them of that decision.

Thank you for your time, and please do not hesitate to contact the researchers with any questions.

I, (print your name)_________________________________, **am refusing** to give permission for my child, (print child's name)_____________________________, who participates in a 4-H club located in (print town or city)___________________, to participate in the research described in this form.

____ ________________

Parent Signature (only if **refusing child participation**) Date

UNIVERSITY OF CALIFORNIA | PARENT LETTER

Project Title: Close to Home Program Evaluation

Principal Investigator: Dr. Jay Silverman, Professor, University of California San Diego
Phone: (858) 246-0066; Email: jgsilverman@health.ucsd.edu

Close to Home Contact: [Name]; Phone: [Phone number]; Email: [Email address]

What is the purpose of this study?

The purpose of this research study is to understand the effectiveness of a community-based program to reduce and change the acceptability of sexual violence and dating violence and related outcomes in California. The study will help researchers learn more about how to best reduce sexual violence and dating violence and promote the safety and well-being of all in the community.

What is the purpose of this form?

This letter gives you the information you will need to help you decide whether you want your child to be in the study or not. Please read the form carefully. You will only sign and return this form if you **do not agree** to give your child permission to participate in the study.

Your child is being invited to participate in the study because they have participated in Close to Home programming. You can contact the researcher to explain anything that you don't understand. You may ask any questions about the research, the possible risks and benefits, your child's rights as a study participant, and anything else that is not clear by contacting the Principal Investigator at the contact information provided above. When all of your questions have been answered, you can decide if you want your child to be in this study or not.

Why is my child being invited to take part in this study?

Your child is a part of a Close to Home club that has been selected for the study. Youth ages 14-24 years in selected Close to Home clubs in California are asked to participate in this study.

What will happen during this study and how long will it take?

- If your child agrees to be in this study, they will be asked to fill out an online survey, which will take approximately 45 minutes. Your child will be recontacted two years from now and invited to take the same survey.
- The first survey will be completed online during a Close to Home Meeting. The second survey will be completed solely online.
- The survey will ask your child questions about knowledge and attitudes related to sexual/dating violence, connection with the community, willingness and confidence to prevent sexual/dating violence among peers, and whether they have experienced or committed sexual violence, dating violence, and sexual harassment.
- Your child can choose to decline to answer any question. Their responses will be stored separately from any information that identifies them so no one outside the UC San Diego

research team can link them together. Their identity is protected from ever being used in any local, state, or federal legal proceedings, or for any other purposes, related to these data.

- During the survey, your child will also be asked to nominate up to 10 peers whom they feel close to (for example, close friends from school or from outside of school) who are between the ages of 14-24 to take the same survey. Your child will be sent a text message from UC San Diego that they can forward to their peers explaining that they are nominating them to participate in the study. Your child's friends will not know that your child took the survey.

Please read through the information below for more details about what the research entails:

What are the risks of this study?

The survey includes questions on experiences of and exposure to sexual violence and dating violence. Your child may feel nervousness or discomfort while answering these questions. In case your child feels any discomfort, they can decline to answer any questions about their own experiences with sexual and/or dating violence, and they can withdraw at any time by simply exiting the survey. Choosing not to answer any question or withdrawing from the survey will not result in any penalty.

We will not tell anyone what your child reports on the survey unless they report something that could be dangerous to them or someone else. This research is covered by a **Certificate of Confidentiality** from the Centers for Disease Control and Prevention. The researchers are the only people who are supposed to know information about your child and the answers your child gives to our questions, but there is a possibility that they may share answers with others, or another child may disclose that your child participated in the research.

We will ask your child to nominate up to 10 peers from their social circle to take the same survey. We will reach out to your child's peers using an automated text message with a link to the same survey. However, before we reach out to them, we will ask your child to send their peers a text message informing them about the study. This text message will read as follows and is all that we are asking your child to send:

Hello <NAME>,

Researchers at the University of California, San Diego are conducting a study to understand how to prevent sexual violence in California. I have nominated you to take the survey for this study.

- *The survey takes 45 minutes.*
- *The survey includes some questions about your experiences with sexual and/or dating violence.*
- ***You will receive a $20 e-gift card for completing the survey, plus $10 for each peer you nominate who opts-in to learning more about the survey (up to $100 in e-gift cards).***
- *Your identity and responses will be kept completely confidential.*
- *Participating in this survey will contribute to understanding the way sexual violence can be prevented in California.*

You will also receive an automated text from the researchers at UC San Diego with more information about the survey and a link to take the survey. You are free to participate or decline to participate in the survey. If you decline, there is no penalty.

This will help us in making sure that their peers don't consider the automated text message from the research team as "spam". However, this will also mean that their peers will know that your child nominated them, which may make them feel some nervousness or discomfort. Your child's peers, though,

will not be told whether or not your child participated in the study, nor will your child be told if their peers decide to participate in the study. Again, your child can choose to not complete this section or withdraw from the study at any time without penalty.

Because this is a research study, there may also be some unknown risks that are currently unforeseeable. Your child will be informed of any significant new risks that are identified.

What are the limitations to Certificate of Confidentiality?

Information or documents protected by this Certificate cannot be disclosed to anyone else who is not connected with the research *unless*:

1. Your child chooses to report that they are in danger.
    i. Although the survey does not include questions on whether or not they are currently being hurt, if your child chooses to tell a member of the study team that an adult is or has been hurting them and they are under age 18, we have to report this to the authorities who are responsible for protecting children so they can make sure your child is safe;
    ii. Regardless of your child's age, if your child chooses to tell us that they are a danger to themselves, we also will need to report that to people who can help them. Again, we do not ask questions about this on the survey;
2. Your child is under age 18 and they choose to report to the research team that they have had or are having sexual contact with someone age 18 or older. In this case, we have to report this to authorities who are responsible for protecting children so they can make sure your child is safe. The survey does not ask your child to provide this information.
3. Your child chooses to report to the research team that someone else is in imminent danger. If your child tells us that they or someone else has the intention of seriously harming someone else, we have to report this to authorities who can help ensure their safety. The survey does not ask your child to provide this information.

Your child's data will be used for scientific research, as allowed by federal regulations protecting research subjects, but will never be used in any way that identifies who they are.

What are the benefits of this study?

There are no direct benefits to your child participating in this study. The researchers and community organizations, however, may learn more about how to best reduce sexual violence and dating violence and promote safety and well-being of all in the community. Your child will receive information about free or low-cost local support services after completion of the survey.

Will your child be paid for participating?

If they participate in the online survey, they will be sent a $20 e-gift card upon finishing the survey and will be sent additional $10 e-gift cards for each peer they nominate who opts-in to learning about the survey (up to an additional $100 in e-gift cards, up to $120 in total. Your child will be recontacted two years from now and invited to take the same survey. They will receive $30 for completing the second survey.

The gift cards are distributed through your child's email address by Tango Card. Tango Card keeps emails on file for internal and service-related purposes only. Tango Card does not sell Personally

Identifiable Information to third parties. You can read Tango Card's Reward Recipient Privacy Notice here.

There will be no costs to you or your child for taking part in this study.

Who will see the information that your child provides?

The information your child provides through this survey will be kept confidential to the extent permitted by federal and state law. All names and any other information that might identify your child will be removed immediately after your child completes their participation in the study. All data collected through this survey will be stored on password-protected computers and drives, and any printed data will be stored in a locked cabinet, in a locked office, and only the investigator will have access to the data in its entirety. If the results of this project are published, your child's identity will not be made public. Other UC San Diego and UC Davis staff will have access to data after potentially identifiable characteristics have been removed, or data as reported in aggregate.

This identifiable information (first name and first two initials of last name, email, and phone number and peer's first name, first two initials of last name, and email or phone number) will be handled as confidentially as possible. However, individuals from UC San Diego and UC Davis who oversee research may access data during audits or other monitoring activities. Research team members are mandated reporters and may learn of information that requires reporting. The identifiable information provided by your child will be stored in a secure, encrypted data collection platform in order to distribute gift cards, generate a text message and unique link to recruit nominated peers, and to recontact your child regarding follow-up data collection. Neither staff working for the data collection platform, nor the UCD research team will have access to the names and phone numbers. Only 2 members of the UCSD research team will have access to these forms of identifiable information for the sole purposes of distributing gift cards, generating a text message and unique link to recruit nominated peers, and recontacting your child regarding follow-up data collection. The identifiable information your child provides about themselves will be kept securely until follow-up data collection is complete (2 years after they first take the survey) and then deleted. The identifiable information your child provides about peers will be deleted after 14 days from the data management platform and not used for any other purposes.

Does your child have a choice to be in this study?

Your child's participation in this study is completely voluntary. Your child will not be treated differently if you or they decide to decline participation. Your decision will not result in any loss or benefits to which you or your child are otherwise entitled. Your child can stop at any time during the study without penalty. Your child is free to skip any question they do not wish to answer. You are free to not allow your child to participate in this study without adversely affecting your or your child's relationship with the investigator, University of California, or Close to Home.

What If I have questions?

If you have questions about the study, you can contact the principal investigator: Dr. Jay Silverman, jgsilverman@health.ucsd.edu, or his research team at (858) 246-0066; or the Close to Home program facilitator (name and contact information at the top of this form). If you have questions about your rights or your child's rights as a research participant or you would like to report a concern or complaint about this study, please contact the UC San Diego Human Research Protections Program Office at 858-246-HRPP (858-246-4777)

How do I refuse permission for my child to participate?

If you **do not agree** to give your child permission to participate in the study, please fill the following **opt-out** form.

Study Opt-Out Form

Please indicate below if you **do not agree** to give your child permission to participate in this survey by filling in your name and signature, the name of your child, and the town/city of your child's Close to Home club. If you do not want your child to participate in this study, please inform them of that decision.

Thank you for your time, and please do not hesitate to contact the researchers with any questions.

I, (print your name)________________________________, **am refusing** to give permission for my child, (print child's name)____________________________, who participates in a Close to Home club located in (print town or city)___________________, to participate in the research described in this form.

____ ____________________

Parent Signature (only if **refusing child participation**) Date
