# Supplementary material for: Evaluation of 24-Month Effects of the Close to Home Program on Youth Sexual and Dating Violence Across 22 Communities in California: Protocol for a Quasi-Experimental Cluster-Matched Control Trial
Source: JMIR Res Protoc. 2026 Jul 15;15:e81249. doi: 10.2196/81249 (PMC13372292; doi:10.2196/81249)
Supplement: Multimedia Appendix 2 — Close to Home evaluation participant timeline. [file resprot-v15-e81249-s002.pdf]

Close to Home Evaluation Participant Timeline: Schedule of enrollment, interventions, and assessments.[a]

	TRIAL PERIOD					
	Enrollment		Retention Check-Ins			Close-out
TIMEPOINT[b]	$-t_i$ to 0	0	*6m*	*12m*	*18m*	*24m*
ENROLLMENT:						
Eligibility screen	X					
Informed consent/assent	X					
Control Selection & Assignment	X					
EXPOSURE:						
Intervention		X	→	→	X	
Control Program		X	→	→	X	
ASSESSMENTS:						
Baseline Social Network Survey		X				
Follow-up Social Network Survey						X
Baseline California Healthy Kids Survey		X				
Follow-up California Healthy Kids Survey						X

Citation: Chan A-W, Boutron I, Hopewell S, Moher D, Schulz KF, et al. SPIRIT 2025 statement: updated guideline for protocols of randomised trials. BMJ 2025;389:e081477. https://dx.doi.org/10.1136/bmj-2024-081477

© 2025 Chan A-W et al. This is an Open Access article distributed under the terms of the Creative Commons Attribution License (https://creativecommons.org/licenses/by/4.0/), which permits unrestricted use, distribution, and reproduction in any medium, provided the original work is properly cited.
