# Supplementary material for: Evaluation of 24-Month Effects of the Close to Home Program on Youth Sexual and Dating Violence Across 22 Communities in California: Protocol for a Quasi-Experimental Cluster-Matched Control Trial
Source: JMIR Res Protoc. 2026 Jul 15;15:e81249. doi: 10.2196/81249 (PMC13372292; doi:10.2196/81249)
Supplement: Multimedia Appendix 3 — Measures data. [file resprot-v15-e81249-s003.pdf]

MEASURES

Contents

I. Demographics & violence prevention experience
II. E.3.1. Behavioral attitudes related to SV
III. E.3.2. Behavioral intentions
IV. E.3.3. Self-efficacy to prevent SV
V. E.3.4. Participation in SV prevention
VI. E.3.5. Perceived behavioral control
VII. E.3.6. Sexual violence behaviors
VIII. E.3.7. Social capital
IX. E.3.8. Social norms
X. E.3.9. Name generator and egocentric items

**Demographics & violence prevention experience**<br>***2 items included in custom CHKS module***			
**Sources:** *UCSD research team, California Healthy Kids Survey, US Transgender Survey 2015; Validation of identity abuse scale among LGBTQ individuals*<br>Scheer J, Woulfe JM, Goodman LA. Psychometric validation of the identity abuse scale among LGBTQ individuals. Community Psychology. 2018.			
**Item #**	**Item**	**Response Options**	**Skip pattern**
1	What is your gender identity? (It's okay if this is different than your sex or gender assigned at birth.) (Check all that apply)<br>***Included in custom CHKS module***	☐ Man/Boy- cisgender (you identify as a man/boy and your sex/gender assigned at birth was the same)<br>☐ Woman/Girl- cisgender (you identify as a woman/girl and your sex/gender assigned at birth was the same)<br>☐ Man/Boy- transgender (you identify as a man/boy and your sex/gender assigned at birth was different)<br>☐ Woman/Girl- transgender (you identify as a woman/girl and your sex/gender assigned at birth was different)<br>☐ Non-binary (you do not identify as a man or a woman)<br>☐ Gender fluid (your gender identity changes and fluctuates over time)<br>☐ Gender non-conforming/genderqueer (you do not conform to gender norms/your gender exists outside of cisgender frameworks of gender)<br>☐ Agender (you do not identify with the experience or idea of gender)<br>☐ Exploring/questioning (you haven't labeled your gender identity)<br>☐ Prefer to self-describe ______<br>☐ Prefer not to answer	
2	What is your sexual orientation? (Check all that apply)<br>*By "sexual orientation" we mean your sexual and emotional attraction to another person.*<br>***Included in custom CHKS module***	☐ Straight (you are a man attracted to women, or a woman attracted to men)<br>☐ Lesbian or Gay (you are attracted to people of the same gender or sex)<br>☐ Bisexual (you are attracted to people of the same gender or sex and other genders and sexes)	

		☐ Pansexual (you are attracted to people of all genders and sexes)<br>☐ Asexual (you experience no or limited sexual attraction)<br>☐ Demisexual (you are attracted to people who you have a strong emotional connection to)<br>☐ Queer/LGBQ+ (umbrella term meaning not straight. LGBQ+ means lesbian, gay, bisexual, queer +)<br>☐ Exploring/Questioning (you haven't labeled your sexual orientation)<br>☐ Prefer to self-describe: _______<br>☐ Prefer not to answer	
3	What is your race or ethnicity? (Check all that apply)	☐ American Indian or Alaska Native<br>☐ Asian or Asian American<br>☐ Black or African American<br>☐ Hispanic or Latino/a/x<br>☐ Native Hawaiian or Pacific Islander<br>☐ White<br>☐ Other<br>☐ Prefer not to answer	
4	What grade in school are you in now (or educational status if you completed high school)?	☐ 6th grade<br>☐ 7th grade<br>☐ 8th grade<br>☐ 9th grade<br>☐ 10th grade<br>☐ 11th grade<br>☐ 12th grade<br>☐ Graduated from high school/passed GED exam<br>☐ Working toward GED<br>☐ Did not finish high school and not working toward GED<br>☐ Some trade or technical school<br>☐ Some college or university<br>☐ Completed college or university degree<br>☐ Prefer not to answer	

	Are you currently in homeschool instead of attending a public or private school for classes? *Being homeschooled <u>does NOT</u> include people who typically attend a public or private school in-person but now attend virtual classes because of Covid-19 restrictions.*	☐ Yes, I am currently homeschooled (even when Covid-19 restrictions are over)<br>☐ No, but I used to be homeschooled for all or part of my education (before Covid-19 restrictions started)<br>☐ No, I have never been homeschooled<br>☐ Prefer not to answer	Conditional logic: show if currently in grades 6-12 (from previous question)
	Were you ever in homeschool instead of attending a public or private school for classes when you were in grades 1-12? *Being homeschooled <u>does NOT</u> include people who typically attend a public or private school in-person but attended virtual classes because of Covid-19 restrictions.*	☐ Yes, I used to be homeschooled for all or part of my education (before Covid-19 restrictions started)<br>☐ No, I have never been homeschooled<br>☐ Prefer not to answer	Conditional logic: show if currently educational status is any of these: (from education question)<br>☐ Graduated from high school/passed GED exam<br>☐ Working toward GED<br>☐ Did not finish high school and not working toward GED<br>☐ Some trade or technical school<br>☐ Some college or university<br>☐ Completed college or university degree
5	Did you receive an in-person or virtual/online introduction to be a part of this study?	☐ In-person<br>☐ Virtual/online<br>☐ Not sure<br>☐ Prefer not to answer	Conditional logic: Only show to egos

6	In the past year, have you learned about preventing dating violence and/or sexual violence from someone in your community or school? (Check all that apply)	☐ Yes, in a small group at school or in my community<br>☐ Yes, as part of my school classes<br>☐ Yes, at a school-wide event<br>☐ Yes, at a community event<br>☐ Yes, from a friend<br>☐ Yes, from a family member<br>☐ Yes, from a poster or flyer<br>☐ Yes, from some other source<br>☐ No<br>☐ Prefer not to answer	Conditional logic: show if selected any 'Yes' for item 6.<br><br>How long have you been in (or were you in) this group?<br>☐ 0-3 months<br>☐ 3-6 months<br>☐ 6 months-1 year<br>☐ 1-2 years<br>☐ 2-3 years<br>☐ More than 3 years<br>☐ Prefer not to answer
7	In the past year, have you been a part of an effort to organize and bring together community members to work on an important issue in the community? (Check all that apply)	☐ Yes, related to preventing sexual violence and/or dating violence<br>☐ Yes, related to a social justice movement (for example, Black Lives Matter, #MeToo, Stop Asian Hate, etc.)<br>☐ Yes, related to COVID-19 prevention<br>☐ Yes, related to mental health<br>☐ Yes, related to some other community problem (Please list the other community problem: __________)<br>☐ No<br>☐ Prefer not to answer	Conditional logic: show if selected any 'Yes' for item 7.<br><br>If so, how long have you been in (or were you in) these effort/these efforts<br>☐ 0-3 months<br>☐ 3-6 months<br>☐ 6 months-1 year<br>☐ 1-2 years<br>☐ 2-3 years<br>☐ More than 3 years<br>☐ Prefer not to answer

E.3.1. Behavioral attitudes related to SV

Included in custom CHKS module

Included in YouGov survey module

Source: *Illinois Rape Myth Scale (IRMA) adapted for Evaluation of Green Dot (modified for this evaluation)*
Coker, A. L., Cook-Craig, P. G., Williams, C. M., Fisher, B. S., Clear, E. R., Garcia, L. S., & Hegge, L. M. (2011). Evaluation of Green Dot: An active bystander intervention to reduce sexual violence on college campuses. Violence Against Women, 17, 777–796.

The next section and several later sections contain statements about sexual and/or dating violence. We recognize that these questions can be activating. Please take your time and step away if you need to. If at any point you do not feel comfortable continuing, that is okay. You can choose to stop taking the survey at any time by exiting the browser. If you exit the browser but then decide later that you want to finish the survey, you can use the same link to open it back up and finish it within 7 days. You can also choose not to answer any question by selecting "Prefer not to answer".
The following statements discuss cisgender boys/men and girls/women. We acknowledge that these statements are relevant to other genders as well.

**Please tell us the extent to which you agree or disagree with this statement:**			
9	Rape accusations are often used by girls as a way of getting back at guys.	☐ Strongly disagree<br>☐ Disagree<br>☐ Somewhat disagree<br>☐ Somewhat agree<br>☐ Agree<br>☐ Strongly agree<br>☐ Prefer not to answer	
10	It is usually only girls who dress suggestively that are raped.		
11	Girls tend to exaggerate how much rape affects them.		
12	A lot of girls lead a guy on, and then they claim rape.		
13	Girls that 'tease' guys deserve anything that might happen to them.		
14	When girls are raped, it is often because the way they said 'no' was unclear.		
15	A girl who dresses in skimpy clothes is asking for a guy to force her to have sex.		
**E.3.2. Behavioral intentions**<br>***Included in YouGov survey module***			
**Source:** *Coaching Boys into Men (CBIM)- Intentions to intervene scale (modified for this evaluation)*<br>Miller, E., Jones, K. A., Ripper, L., Paglisotti, T., Mulbah, P., & Abebe, K. Z. (2020). An athletic coach–delivered middle school gender violence prevention program: A cluster randomized clinical trial. *JAMA pediatrics*, *174*(3), 241-249.<br>Miller, E., Tancredi, D. J., McCauley, H. L., Decker, M. R., Virata, M. C. D., Anderson, H. A., ... & Silverman, J. G. (2012). "Coaching boys into men": A cluster-randomized controlled trial of a dating violence prevention program. *Journal of adolescent health*, *51*(5), 431-438.			
The following statements focus on cisgender boys or men doing a behavior. We acknowledge that other genders also do these behaviors, and that cisgender boys and men can also be the target of these behaviors.			
**How would you react if you saw or heard a boy or man you know…**			
16	Telling sexual jokes that disrespect women and girls?	☐ I would laugh or go along with it<br>☐ I wouldn't say or do anything<br>☐ I would say or do something to let them know that I think acting like that is not okay<br>☐ Prefer not to answer	
17	Telling sexual jokes that disrespect LGBTQ+ people?		
18	Showing other people either sexual messages from a romantic partner or showing them naked/sexual pictures of a romantic partner?		
19	Doing unwelcome or uninvited sexual things towards a person (or group of people), such as "cat-calling", whistling, making sexual gestures, or touching?		
**E.3.3. Self-efficacy to prevent SV**			

**Source:** *Community Violence Prevention Activation Measure (CV-PAM): Confidence and Knowledge to Take Action (modified for this evaluation)*<br>Dinizulu, S. M., Suarez, L. M., Simpson, D., Abdul-Adil, J., & Jacobson, K. C. (2020). Psychometric properties of the Community Violence-Prevention Activation Measure (CV-PAM): Evaluating provider activation toward community violence prevention. *Journal of community psychology*, *48*(2), 545-561.			
20	I am confident in my ability to take actions to prevent sexual or dating violence.	☐ Strongly disagree<br>☐ Disagree<br>☐ Somewhat disagree<br>☐ Somewhat agree<br>☐ Agree<br>☐ Strongly agree<br>☐ Prefer not to answer	
21	I know ways to prevent sexual or dating violence.		
22	I know how to talk with others about ways to prevent sexual or dating violence.		
**E.3.4. Participation in SV prevention**			
**Source:** *California Healthy Kids survey - Community Health Module (modified for this evaluation)*			
23	How many times in the past 12 months did you participate in a program or action to create positive change around sexual and/or dating violence prevention?	☐ Never in the past year<br>☐ Once or twice in the past year<br>☐ Three to five times in the past year<br>☐ Six to ten times in the past year<br>☐ More than ten times in the past year<br>☐ Prefer not to answer	
**Source:** *Bystander Intention to Help a Stranger Scale (modified for this evaluation)*<br>Banyard, V. L., Moynihan, M. M., Cares, A. C., & Warner, R. (2014). How do we know if it works? Measuring outcomes in bystander-focused abuse prevention on campuses. Psychology of Violence, 4(1), 101.			
24	I talked with people about sexual violence or dating violence.	Check all that apply (excluding "I didn't do this with anyone")<br>☐ I didn't do this with anyone in the past 12 months<br>☐ I did this with my close friends or family in the past 12 months<br>☐ I did this with people I know, but who are not my close friends or family, in the past 12 months<br>☐ I did this with people I don't know in the past 12 months<br>☐ I did this with people I know from online in the past 12 months<br>☐ Prefer not to answer	Conditional logic: show if answered any 'I did this…'<br><br>How did you talk with people about sexual violence and/or dating violence? (Check all that apply):<br>☐ In-person<br>☐ Via text<br>☐ Via phone call<br>☐ Via video call (for example, Facetime, Zoom, etc.)<br>☐ Online<br>☐ Other<br>☐ Prefer not to answer
25	I talked with people about abusive relationships and the warning signs of an abusive relationship.		
26	I shared information about where or how people can get help for sexual violence and/or dating violence (For example, local organizations or hotlines).		

E.3.5. Perceived behavioral control

Source: *CBIM Intentions to intervene scale & SV intervention behaviors scale (modified for this evaluation)*
Miller, E., Jones, K. A., Ripper, L., Paglisotti, T., Mulbah, P., & Abebe, K. Z. (2020). An athletic coach–delivered middle school gender violence prevention program: A cluster randomized clinical trial. *JAMA pediatrics*, *174*(3), 241-249.
Miller, E., Tancredi, D. J., McCauley, H. L., Decker, M. R., Virata, M. C. D., Anderson, H. A., ... & Silverman, J. G. (2012). "Coaching boys into men": A cluster-randomized controlled trial of a dating violence prevention program. *Journal of adolescent health*, *51*(5), 431-438.

How confident are you that you could say or do something to stop a friend or another peer who was…

27	Telling sexual jokes that disrespect women and girls?	☐ Not confident at all<br>☐ Not confident<br>☐ Somewhat confident<br>☐ Confident<br>☐ Very confident<br>☐ Not applicable, I approve of this behavior and would not try to stop it<br>☐ Prefer not to answer	
28	Telling sexual jokes that disrespect LGBTQ+ people?		
29	Doing unwelcome or uninvited sexual things toward a person (or group of people) such as catcalling, whistling, making sexual gestures, or touching?		
30	Showing other people either sexual messages from a romantic partner or showing them naked/sexual pictures of a romantic partner?		
31	Making rude or disrespectful comments about someone's body, clothing, or make-up?		
32	Spreading rumors about someone's sexual reputation, like saying "they're easy to get with"?		
33	Fighting with a dating/romantic partner to the point where they're starting to cuss at or threaten them		
34	Shoving, grabbing, or otherwise physically hurting a dating/romantic partner?		

E.3.6. Sexual violence behaviors

E.3.6.1. Bystander behavior

Source: *Coaching Boys into Men Evaluations – SV intervention behavior scale (modified for this evaluation)*
Miller, E., Jones, K. A., Ripper, L., Paglisotti, T., Mulbah, P., & Abebe, K. Z. (2020). An athletic coach–delivered middle school gender violence prevention program: A cluster randomized clinical trial. *JAMA pediatrics*, *174*(3), 241-249.
Miller, E., Tancredi, D. J., McCauley, H. L., Decker, M. R., Virata, M. C. D., Anderson, H. A., ... & Silverman, J. G. (2012). "Coaching boys into men": A cluster-randomized controlled trial of a dating violence prevention program. *Journal of adolescent health*, *51*(5), 431-438.

In the past 12 months, have you seen or heard someone around your age…

35	Telling sexual jokes that disrespect women and girls?	☐ Yes, I saw or heard someone around my age do this in the past 3 months	Conditional logic: show if *any YES*<br>☐ Woman or Girl?
36	Telling sexual jokes that disrespect LGBTQ+ people?		

37	Doing unwelcome or uninvited sexual things toward a person (or group of people) such as catcalling, whistling, making sexual gestures, or touching?	☐ Yes, I saw or heard someone around my age do this in the past 12 months, but not in the past 3 months<br>☐ No, I did not see or hear someone around my age do this in the past 12 months<br>☐ Prefer not to answer	☐ Man or Boy?<br>☐ Someone who is non-binary or another gender?<br>☐ Prefer not to answer<br><br>Please check all that apply:<br>☐ I didn't say or do anything<br>☐ I laughed or went along with it<br>☐ I told the person in public that acting like that was not okay<br>☐ I told the person in private that acting like that was not okay<br>☐ I talked to an adult about it later<br>☐ I talked to a friend about it later<br>☐ Prefer not to answer
38	Showing other people either sexual messages from a romantic partner or showing them naked/sexual pictures of a romantic partner?		
39	Making rude or disrespectful comments about someone's body, clothing, or make-up?		
40	Spreading rumors about someone's sexual reputation, like saying "they're easy to get with"?		
41	Fighting with a dating/romantic partner to the point where they're starting to cuss at or threaten them?		
42	Shoving, grabbing, or otherwise physically hurting a dating/romantic partner?		

E.3.6.2. SV victimization/perpetration

The next section contains specific questions regarding your own experiences with sexual violence and dating violence. As a reminder, the information you share with us is confidential and any identifying information will not be shared without your consent. We recognize that these questions are personal and can be activating. Please take your time and step away if you need to. If at any point you do not feel comfortable continuing, that is okay. You can choose not to answer the question by selecting "prefer not to answer" or to stop taking the survey at any time by exiting the browser. If you exit the browser but then decide you want to finish the survey, you can use the same link to open it back up and finish it within 7 days.

In the next set of questions, we ask about dating and romantic partners. When we use the term "dating/romantic partner" in this survey we mean a boyfriend, girlfriend, or someone you consider to be "more than just a friend" (i.e., someone you've gone out with or had a sexual relationship with, or a "friend with benefits").

Virtual/online sexual harassment

Included in CHKS module

Source: *Digital dating abuse scale & CBIM Victimization scale (both modified for this evaluation)*

Reed, L. A., Tolman, R. M., & Ward, L. M. (2017). Gender matters: Experiences and consequences of digital dating abuse victimization in adolescent dating relationships. *Journal of adolescence*, *59*, 79-89.

Miller, E., Jones, K. A., Ripper, L., Paglisotti, T., Mulbah, P., & Abebe, K. Z. (2020). An athletic coach–delivered middle school gender violence prevention program: A cluster randomized clinical trial. *JAMA pediatrics*, *174*(3), 241-249.

In the past 12 months, did the following ever happen to you online, over text or through social media?

43	Someone pressured you to send a sexual or naked photo of yourself to them.	☐ Yes, this happened in the past 12 months<br>☐ Yes, this happened but not in the past 12 months<br>☐ No, this has never happened<br>☐ Prefer not to answer	Conditional logic: show if selected any 'Yes' for item 43-44.<br>The person who did this was/is (Check all that apply):<br>☐ A dating/romantic partner<br>☐ Someone else<br>☐ Prefer not to answer
44	Someone pressured you to have sex or do something sexual when you didn't want to.		
**In-person sexual harassment/sexual violence**<br>***Included in custom CHKS module**			
**Source:** *Teen health and technology survey 2010/11 & CBIM Victimization scale (both modified for this evaluation)*<br>Mitchell, K. J., Ybarra, M. L., & Korchmaros, J. D. (2014). Sexual harassment among adolescents of different sexual orientations and gender identities. *Child abuse & neglect*, *38*(2), 280-295.<br>Miller, E., Jones, K. A., Ripper, L., Paglisotti, T., Mulbah, P., & Abebe, K. Z. (2020). An athletic coach–delivered middle school gender violence prevention program: A cluster randomized clinical trial. *JAMA pediatrics*, *174*(3), 241-249.			
**In the past 12 months, did the following ever happen to you in-person?**			
45	Someone touched, grabbed, or pinched you or grabbed your clothing in a sexual way when you didn't want them to.	☐ Yes, this happened in the past 12 months<br>☐ Yes, this happened but not in the past 12 months<br>☐ No, this has never happened<br>☐ Prefer not to answer	Conditional logic: show if selected any 'Yes' for item 45-46.<br><br>The person who did this was/is (Check all that apply):<br>☐ A dating/romantic partner<br>☐ Someone else<br>☐ Prefer not to answer
46	Someone pressured you or made you have sex or do something sexual when you didn't want to.		
**E.3.6.2.a. LGBTQ+ specific SV/SH victimization**<br>***6 items included in custom CHKS module**			
**Source:** *Identity abuse scale (modified for this evaluation)*<br>Scheer J, Woulfe JM, Goodman LA. Psychometric validation of the identity abuse scale among LGBTQ individuals. Community Psychology. 2018.			
47a	Someone threatened to tell people at school, your family, or others about your sexual orientation or gender identity<br>***Included in custom CHKS module**	☐ Yes, this happened in the past 12 months<br>☐ Yes, this happened but not in the past 12 months<br>☐ No, this has never happened<br>☐ Prefer not to answer	Receive 47a-e if the participant selected any gender other than cisgender options OR selected any sexual identity other than straight.
47b	Someone forced you to show physical or sexual affection in public even though you didn't want to		

47c	Someone forced you to participate in particular sexual behaviors to prove your gender or sexual orientation was 'real'<br>***Included in custom CHKS module***		Conditional logic: show if selected any 'Yes' for item 47a-e.<br><br>The person who did this was/is (Check all that apply):<br>☐ A dating/romantic partner<br>☐ Someone else<br>☐ Prefer not to answer
47d	Someone told you that you don't deserve to be treated well or respected because of your sexual orientation or gender identity		
47e	Someone called you disrespectful or offensive names that have to do with your LGBTQ+ identity<br>***Included in custom CHKS module***		
**Source:** *Transgender-specific intimate partner violence (T-IPV) scale (modified for this evaluation)*<br>Peitzmeier SM, Wirtz AL, Humes E. et al. The transgender-specific intimate partner violence scale for research and practice: Validation in a sample of transgender women. Social Science & Medicine. 2021. 291: 114495.			
48a	Someone forced or pressured you into doing something that did not agree with your gender identity, such as pressuring you to use a bathroom that did not align with your gender identity, or pressuring you to change your gender presentation<br>***Included in custom CHKS module***	☐ Yes, this happened in the past 12 months<br>☐ Yes, this happened but not in the past 12 months<br>☐ No, this has never happened<br>☐ Prefer not to answer	Receive 48a-d if participant selected any other gender other than cisgender.<br><br>Conditional logic: show if selected any 'Yes' for item 48a-d.<br><br>The person who did this was/is (Check all that apply):<br>☐ A dating/romantic partner<br>☐ Someone else<br>☐ Prefer not to answer
48b	Someone pressured you to stay in a relationship by telling you that you would never find someone else who "would want to date or be with someone like you"		
48c	Someone hid or destroyed your hormones, prosthetics, chest binders, clothing, etc., or other gender affirming items related to your gender transition or presentation<br>***Included in custom CHKS module***		
48d	Someone tried to touch you, or did touch you, to try to 'figure out' your gender<br>***Included in custom CHKS module***		
**Dating violence victimization**<br>***Included in custom CHKS module***			
**Source:** *CBIM Victimization scale (modified for this evaluation)*<br>Miller, E., Jones, K. A., Ripper, L., Paglisotti, T., Mulbah, P., & Abebe, K. Z. (2020). An athletic coach–delivered middle school gender violence prevention program: A cluster randomized clinical trial. *JAMA pediatrics*, *174*(3), 241-249.			

Miller, E., Tancredi, D. J., McCauley, H. L., Decker, M. R., Virata, M. C. D., Anderson, H. A., ... & Silverman, J. G. (2012). “Coaching boys into men”: A cluster-randomized controlled trial of a dating violence prevention program. *Journal of adolescent health*, *51*(5), 431-438.			
**In the past 12 months, did a dating/romantic partner do any of the following to you?**			
50	Told you not to talk to certain people, or told you who you could and couldn’t hang out with	☐ Yes, this happened in the past 12 months<br>☐ Yes, this happened but not in the past 12 months<br>☐ No, this has never happened<br>☐ Prefer not to answer	
**E.3.6.2.a. LGBTQ+ specific SV/SH victimization**<br>***1 item included in custom CHKS module***			
**Source:** *Identity abuse scale & Transgender-specific intimate partner violence (T-IPV) scale (modified for this evaluation)*<br>Scheer J, Woulfe JM, Goodman LA. Psychometric validation of the identity abuse scale among LGBTQ individuals. Community Psychology. 2018.<br>Peitzmeier SM, Wirtz AL, Humes E. et al. The transgender-specific intimate partner violence scale for research and practice: Validation in a sample of transgender women. Social Science & Medicine. 2021. 291: 114495.			
51a	Prevented you from seeking support from other people within the LGBTQ+ community<br>***Included in custom CHKS module***	☐ Yes, this happened in the past 12 months<br>☐ Yes, this happened but not in the past 12 months<br>☐ No, this has never happened<br>☐ Prefer not to answer	Receive this question if the participant selected any gender other than cisgender options OR selected any sexual identity other than straight.
51b	Intentionally and repeatedly used gendered language that did not align with your gender identity	☐ Yes, this happened in the past 12 months<br>☐ Yes, this happened but not in the past 12 months<br>☐ No, this has never happened<br>☐ Prefer not to answer	Receive this question if the participant selected any other gender other than cisgender.
**Virtual/online sexual harassment perpetration**<br>***1 item included in custom CHKS module***			
**Source:** *Digital dating abuse scale (modified for this evaluation)*<br>Reed, L. A., Tolman, R. M., & Ward, L. M. (2017). Gender matters: Experiences and consequences of digital dating abuse victimization in adolescent dating relationships. *Journal of adolescence*, *59*, 79-89. *(modified)*			
**The next set of questions are about behaviors you may have done. As is true with all other questions in this survey, your responses to the following questions are secure, confidential, and will not be shared with anyone. The information you provide cannot be used in any legal proceedings, including civil or criminal proceedings, without your voluntary permission.**<br><br>**In the past 12 months did YOU:**			
52	Ask someone online, through a text, or through social media to send a sexual or naked photo	☐ Yes, I did this in the past 12 months<br>☐ Yes, I have done this but not in the past 12 months	Conditional logic: show if selected any ‘Yes’.

	of themselves after they said they didn't want to do that?<br>_*Included in custom CHKS module_	☐ No, I have never done this<br>☐ Prefer not to answer	I did this to (Check all that apply):<br>☐ A dating/romantic partner<br>☐ Someone else<br>☐ Prefer not to answer
53	Ask someone online, through a text, or through social media to have sex or do something sexual after they said they didn't want to do that?		
**In-person sexual harassment perpetration**<br>_*Included in custom CHKS module_			
**Source:** *Teen Health and Technology survey 2010/11 (modified for this evaluation)*<br>Mitchell, K. J., Ybarra, M. L., & Korchmaros, J. D. (2014). Sexual harassment among adolescents of different sexual orientations and gender identities. *Child abuse & neglect*, *38*(2), 280-295.			
**In the past 12 months, did YOU:**			
54	Touch, grab, or pinch someone or grab their clothing in a sexual way after they said they didn't want you to do that?	☐ Yes, I did this in the past 12 months<br>☐ Yes, I have done this but not in the past 12 months<br>☐ No, I have never done this<br>☐ Prefer not to answer	Conditional logic: show if selected any 'Yes'.<br><br>I did this to: Check all that apply.<br>☐ A dating/romantic partner<br>☐ Someone else<br>☐ Prefer not to answer
**SV perpetration**<br>_*3 items included in custom CHKS module_			
**Source:** *CBIM Intention to interview & SV intervention behaviors scales (modified for this evaluation)*<br>Miller, E., Jones, K. A., Ripper, L., Paglisotti, T., Mulbah, P., & Abebe, K. Z. (2020). An athletic coach–delivered middle school gender violence prevention program: A cluster randomized clinical trial. *JAMA pediatrics*, *174*(3), 241-249.<br>Miller, E., Tancredi, D. J., McCauley, H. L., Decker, M. R., Virata, M. C. D., Anderson, H. A., ... & Silverman, J. G. (2012). "Coaching boys into men": A cluster-randomized controlled trial of a dating violence prevention program. *Journal of adolescent health*, *51*(5), 431-438.			
**In the past 12 months, did YOU do any of the following in-person or online:**			
55	Spread rumors about someone's sexual reputation, like telling people they're 'easy to get with'?	☐ Yes, I did this in the past 12 months<br>☐ Yes, I have done this but not in the past 12 months<br>☐ No, I have never done this<br>☐ Prefer not to answer	Conditional logic: show if selected any 'Yes'.<br><br>I did this to (Check all that apply):<br>☐ A dating/romantic partner<br>☐ Someone else<br>☐ Prefer not to answer<br>I did this (Check all that apply)
56	Show friends pictures of someone you know naked or doing something sexual?<br>_*Included in custom CHKS module_		
57	Convince someone to have sex or do something sexual, after they said they didn't want to do that?<br>_*Included in custom CHKS module_		

<table>
<tr><td>58</td><td>Make someone have sex or do something sexual when they didn’t want to?
*Included in custom CHKS module</td><td></td><td>☐ In-person
☐ Online
☐ Prefer not to answer</td></tr>
<tr><td colspan="4">Dating violence perpetration
*2 items included in custom CHKS module</td></tr>
<tr><td colspan="4">Source: Coaching Boys into Men Evaluations (modified for this evaluation)
Miller, E., Jones, K. A., Ripper, L., Paglisotti, T., Mulbah, P., & Abebe, K. Z. (2020). An athletic coach–delivered middle school gender violence prevention program: A cluster randomized clinical trial. JAMA pediatrics, 174(3), 241-249.
Miller, E., Tancredi, D. J., McCauley, H. L., Decker, M. R., Virata, M. C. D., Anderson, H. A., ... & Silverman, J. G. (2012). “Coaching boys into men”: A cluster-randomized controlled trial of a dating violence prevention program. Journal of adolescent health, 51(5), 431-438.</td></tr>
<tr><td colspan="4">In the past 12 months, did YOU do any of the following to a dating/romantic partner?</td></tr>
<tr><td>59</td><td>Physically hurt them (like shoving, grabbing, slapping, punching, choking), or threatened to hurt them
*Included in custom CHKS module</td><td rowspan="4">☐ Yes, I did this in the past 12 months
☐ Yes, I have done this but not in the past 12 months
☐ No, I have never done this
☐ Prefer not to answer</td><td rowspan="4"></td></tr>
<tr><td>60</td><td>Yelled at them or destroyed something that belonged to them</td></tr>
<tr><td>61</td><td>Called them names, like ‘ugly’ or ‘stupid’</td></tr>
<tr><td>62</td><td>Told them not to talk to certain people or told them who they could and couldn’t hang out with
*Included in custom CHKS module</td></tr>
<tr><td colspan="4">E.3.7. Social capital</td></tr>
<tr><td colspan="4">E.3.7.1. Social capital – community connectedness</td></tr>
<tr><td colspan="4">Source: Community connectedness scale: peers subscale (modified for this evaluation)
Karcher, M. J., Davis III, C., & Powell, B. (2002). The effects of developmental mentoring on connectedness and academic achievement. School Community Journal, 12(2), 35.
Karcher, M. J. (2001). The Hemingway: Measure of Adolescent Connectedness--Validation Studies.</td></tr>
<tr><td>63</td><td>I feel like I belong in my Close to Home/4-H group.</td><td rowspan="4">☐ Strongly disagree
☐ Disagree
☐ Somewhat disagree
☐ Somewhat agree
☐ Agree
☐ Strongly agree
☐ Prefer not to answer</td><td rowspan="4"></td></tr>
<tr><td>64</td><td>I like working with the people in my Close to Home/4-H group.</td></tr>
<tr><td>65</td><td>I get along well with the people in my Close to Home/4-H group.</td></tr>
<tr><td>66</td><td>I have a say about what goes on in my Close to Home/4-H group.</td></tr>
</table>

<table>
<tr><td colspan="4">Community identification – Close to Home (only participants in C2H group see 67-68)</td></tr>
<tr><td colspan="4">Source: UCSD research team</td></tr>
<tr><td>67</td><td>What groups of peers (meaning people around your age) do you feel close and connected to (not including your Close to Home group)? (Check all that apply)</td><td rowspan="2">☐ peers at school
☐ peers from a religious community
☐ peers from an online community
☐ peers from your ethnic community
☐ peers from your neighborhood
☐ peers from where you work
☐ peers on a sports team
☐ peers within your family
☐ peers from another community or group
☐ I don’t feel close or connected to any community or group of peers
☐ Prefer not to answer</td><td>Conditional logic:
If answered “I don’t feel close or connected to any community or group of peers” then skip #68 and #71-72.

If answered “I don’t feel close or connected to any community or group of peers” OR “Prefer not to answer” autofill “peers at school” for #71-72 and for #73-76</td></tr>
<tr><td>68</td><td>Which single group of peers do you feel closest and most connected to (not including your Close to Home group)?</td><td>Conditional logic:
If answered “Prefer not to answer” autofill “peers at school” for #71-72 and for #73-76</td></tr>
<tr><td colspan="4">Community identification – Non-Close to Home (only participants not in C2H group see 69-70)</td></tr>
<tr><td colspan="4">Source: UCSD research team</td></tr>
<tr><td>69</td><td>What groups of peers (meaning people around your age) do you feel close and connected to? (Check all that apply)</td><td rowspan="2">☐ peers at school
☐ peers from a religious community
☐ peers from an online community
☐ peers from your ethnic community
☐ peers from your neighborhood
☐ peers from where you work
☐ peers on a sports team
☐ peers within your family
☐ peers from another community or group
☐ I don’t feel close or connected to any community or group of peers
☐ Prefer not to answer</td><td>Conditional logic:
If answered “I don’t feel close to any community or group of peers” then skip #70-72

If answered “I don’t feel close or connected to any community or group of peers” OR “Prefer not to answer” autofill “peers at school” for #71-72 and for #73-76</td></tr>
<tr><td>70</td><td>Which single group of peers do you feel closest and most connected to?</td><td>Conditional logic:
If answered “Prefer not to answer” autofill “peers at school” for #71-72 and for #73-76</td></tr>
<tr><td colspan="4">Source: Sense of belonging scale (modified for this evaluation)
Fujiwara, T., & Kawachi, I. (2008). Social capital and health: a study of adult twins in the US. American journal of preventive medicine, 35(2), 139-144.</td></tr>
</table>

<table>
<tr><td colspan="4">Thinking of your [community of peers you feel closest (answer to #67/69) OR [peers at school] if answered "Prefer not to Answer" for #67/69], please tell us the extent to which you agree or disagree with the following statements:</td></tr>
<tr><td>71</td><td>I feel close to an adult in this community who is not my parent.</td><td rowspan="2">☐ Strongly disagree
☐ Disagree
☐ Somewhat disagree
☐ Somewhat agree
☐ Agree
☐ Strongly agree
☐ There are no adults in this community
☐ Prefer not to answer</td><td rowspan="2"></td></tr>
<tr><td>72</td><td>This community is a source of comfort.</td></tr>
<tr><td colspan="4">E.3.8. Social norms</td></tr>
<tr><td colspan="4">E.3.8.1. Social norms related to perceived acceptability of SV (injunctive norms)
*Included in custom CHKS module
*Included in YouGov survey module</td></tr>
<tr><td colspan="4">Source: CBIM Intentions to intervene scale (modified for this evaluation)
Miller, E., Jones, K. A., Ripper, L., Paglisotti, T., Mulbah, P., & Abebe, K. Z. (2020). An athletic coach–delivered middle school gender violence prevention program: A cluster randomized clinical trial. JAMA pediatrics, 174(3), 241-249.
Miller, E., Tancredi, D. J., McCauley, H. L., Decker, M. R., Virata, M. C. D., Anderson, H. A., ... & Silverman, J. G. (2012). "Coaching boys into men": A cluster-randomized controlled trial of a dating violence prevention program. Journal of adolescent health, 51(5), 431-438.</td></tr>
<tr><td colspan="4">These next questions focus on cisgender boys or men doing a behavior. We acknowledge that other genders also do these behaviors and that cisgender boys and men can also be the target of these behaviors.
If your [community of peers you feel closest to (answer to #67/69) OR [peers at school] saw or heard a boy or man they know…</td></tr>
<tr><td>73</td><td>Telling sexual jokes that disrespect women and girls</td><td rowspan="4">Your [community of peers you feel closest to (answer to #67/69) OR [peers at school] would be…
☐ Very unlikely to do or say something
☐ Unlikely to do or say something
☐ Likely to do or say something
☐ Very likely to do or say something
☐ Not sure
☐ Prefer not to answer</td><td rowspan="4"></td></tr>
<tr><td>74</td><td>Telling sexual jokes that disrespect LGBTQ+ people</td></tr>
<tr><td>75</td><td>Doing unwelcome or uninvited sexual things toward a person (or group of people), such as cat-calling, whistling, making sexual gestures, or touching</td></tr>
<tr><td>76</td><td>Showing other people either sexual messages from a romantic partner or showing them naked/sexual pictures of a romantic partner</td></tr>
<tr><td colspan="4">Defining Geographic Community</td></tr>
<tr><td colspan="4">Source: UCSD research team</td></tr>
<tr><td colspan="4">Now, we would like for you to think of [Insert geographic location of C2H/control program] as a community.</td></tr>
</table>

<table>
<tr><td>77</td><td>Do you live, go to school in, or work in or close to [Insert geographic location of C2H/control program]?</td><td>☐ Yes, I live there or very close to there
☐ Yes, I go to school there or very close to there
☐ Yes, I live and go to school there or very close to there
☐ No, I do not live or go to school there or very close to there
☐ Prefer not to answer</td><td>Conditional logic:
If select “No, I do not live or go to school there or very close to there” OR ‘Prefer not to answer’, skip 77-87</td></tr>
<tr><td colspan="4">E.3.7.2. Social capital – social cohesion</td></tr>
<tr><td colspan="4">Source: Neighborhood social cohesion scale (modified for this evaluation)
Sampson, R. J., Raudenbush, S. W., & Earls, F. (1997). Neighborhoods and violent crime: A multilevel study of collective efficacy. science, 277(5328), 918-924.</td></tr>
<tr><td colspan="4">Please tell us the extent to which you agree or disagree with the following statements about the [insert geographic location of C2H/control program] community.</td></tr>
<tr><td>78</td><td>People you know in the [geographic location] community are willing to help their neighbors.</td><td rowspan="2">☐ Strongly disagree
☐ Disagree
☐ Somewhat disagree
☐ Somewhat agree
☐ Agree
☐ Strongly agree
☐ I don’t know
☐ Prefer not to answer</td><td rowspan="2"></td></tr>
<tr><td>79</td><td>People you know in the [geographic location] community can be trusted.</td></tr>
<tr><td colspan="4">Source: Brief sense of community scale (modified for this evaluation)
Peterson, N.A., Speer, P.W., & Hughey, J. (2006). Measuring sense of community: A methodological interpretation of the factor structure debate. Journal of Community Psychology, 34, 453–469.</td></tr>
<tr><td>80</td><td>The [geographic location] community helps me fulfill my needs.</td><td rowspan="6">☐ Strongly disagree
☐ Disagree
☐ Somewhat disagree
☐ Somewhat agree
☐ Agree
☐ Strongly agree
☐ Prefer not to answer</td><td rowspan="6"></td></tr>
<tr><td>81</td><td>I feel like a member of the [geographic location] community.</td></tr>
<tr><td>82</td><td>I belong in the [geographic location] community.</td></tr>
<tr><td>83</td><td>I have a say about what goes on in the [geographic location] community.</td></tr>
<tr><td>84</td><td>People in the [geographic location] community are able to influence each other.</td></tr>
<tr><td>85</td><td>I have a good bond with others in the [geographic location] community.</td></tr>
<tr><td colspan="4">E.3.8.2. Social norms related to perceived likelihood of community members intervening to prevent SV (descriptive norms)
*Included in custom CHKS module</td></tr>
</table>

**Source:** *Shared concern scale (modified for this evaluation)*<br>Lippman, S. A., Neilands, T. B., Leslie, H. H., Maman, S., MacPhail, C., Twine, R., ... & Pettifor, A. (2016). Development, validation, and performance of a scale to measure community mobilization. *Social Science & Medicine*, *157*, 127-137.			
86	People you know in the [geographic location] community work together to reduce sexual violence and/or dating violence.	☐ Strongly disagree<br>☐ Disagree<br>☐ Somewhat disagree<br>☐ Somewhat agree<br>☐ Agree<br>☐ Strongly agree<br>☐ I don't know<br>☐ Prefer not to answer	
87	People you know in the [geographic location] community talk to each other about how to reduce sexual violence and/or dating violence.		
88	People you know in the [geographic location] community volunteer to help reduce sexual violence and/or dating violence.		

**E.3.9. Name generator and egocentric items**			
**Source:** *UCSD research team*			
**Of the people in your life who live, go to school, or work in or very close to [geographic location] and are between the ages of 14 and 24…**			
Repeat series (10 times)	Who do you consider a close friend?	Please write in their first name and the first two letters of their last name here.<br><br>If you cannot name anyone, leave the text box blank and continue on to the next question.<br><br>*If you have more than one friend with the same first name and first two letters of their last name, please use nicknames to differentiate between individuals.*	Do you have any other **close friends** who live, go to school, or work in or very close to [geographic location] and are between the ages of 14 and 24?
	Who are the people that you participate in activities with and who you also consider a friend (activities could include things like sports, jobs, online or in-person meetings, after-school clubs, or other groups involving people around your age)?		
	Besides your close friends, who do you talk to about issues that are important to you? (For example, the environment, immigration, politics, sexual violence prevention, etc.)		
**Please rank the following people by dragging and dropping their names in order of how close you feel to them – the person at the top being the person that you are closest to and the person at the bottom being the person that you are least close to.**<br>*By "close" we mean the level at which you trust, care for, and feel comfortable talking about personal matters with a person.*			
Repeat series	How old is [alter]?<br>(You can estimate if you're not sure)	☐ Drop down with ages<br>☐ Prefer not to answer	
	Is [alter] a member of any of these communities?<br>(Please check all that apply)	☐ Your school<br>☐ Your religious community	

		☐ Your online community<br>☐ Your ethnic community<br>☐ Your neighborhood<br>☐ Your work<br>☐ Your sports team<br>☐ Your family<br>☐ Part of another community that you are in<br>☐ Prefer not to answer	
	In the last 6 months, have you talked to [Alter] about sexual violence, sexual harassment, preventing these behaviors, or your work around these topics (if you are involved in this type of work)	☐ Yes<br>☐ No<br>☐ Not Sure<br>☐ This does not apply to me<br>☐ Prefer not to answer	
**Thinking about the past 6 months, please indicate how often you interacted with [alter] in each of the following ways:**			
Repeat series	In person	☐ Every day/nearly every day<br>☐ At least once a week<br>☐ At least once a month<br>☐ Less than once a month<br>☐ Never<br>☐ Prefer not to answer	
	Text		
	Social media (for example, TikTok, Instagram, Snapchat, etc.)		
	Phone calls		
	Video calls (for example, Facetime, Zoom, etc.)		
**If [alter] saw or heard a boy or man they know…**			
Repeat series	Telling sexual jokes that disrespect women and girls	[Alter] would be…<br>☐ Very unlikely to do or say something<br>☐ Unlikely to do or say something<br>☐ Likely to do or say something<br>☐ Very likely to do or say something<br>☐ Not sure<br>☐ Prefer not to answer	
	Telling sexual jokes that disrespect LGBTQ+ people		
	Doing unwelcome or uninvited sexual things towards a person (or group of people), such as "cat-calling", whistling, making sexual gestures, or touching		
	Showing other people either sexual messages from a romantic partner or showing them naked/sexual pictures of a romantic partner		
