# Supplementary material for: Evaluation of 24-Month Effects of the Close to Home Program on Youth Sexual and Dating Violence Across 22 Communities in California: Protocol for a Quasi-Experimental Cluster-Matched Control Trial
Source: JMIR Res Protoc. 2026 Jul 15;15:e81249. doi: 10.2196/81249 (PMC13372292; doi:10.2196/81249)
Supplement: Peer Review Report 1 — Peer review report from the Centers for Disease Control and Prevention of the US Department of Health and Human Services. [file resprot-v15-e81249-s005.pdf]

SUMMARY STATEMENT

PROGRAM CONTACT: (Privileged Communication) *Release Date:* 07/31/2020
Revised Date:

Application Number: 1 U01 CE003201-01

Principal Investigator

SILVERMAN, JAY G

Applicant Organization: UNIVERSITY OF CALIFORNIA, SAN DIEGO

Review Group: ZCE1 KFJ (02)
National Center for Injury Prevention Special Emphasis Panel
CE20-001 - Evaluating Practice-based Programs, Policies, and Practices from CDC's Rape Prevention Education Program

Meeting Date: 04/29/2020 *RFA/PA:* CE20-001
Council: MAY 2020
Requested Start: 09/01/2020

Project Title: Evaluation of the Close to Home Program in California: Assessing the impact of community mobilization to prevent sexual violence at the individual, social network, and community levels
SRG Action: Impact Score:20

Human Subjects: 30-Human subjects involved - Certified, no SRG concerns
Animal Subjects: 10-No live vertebrate animals involved for competing appl.
Gender: 1A-Both genders, scientifically acceptable
Minority: 1A-Minorities and non-minorities, scientifically acceptable
Age: 6A-Children and Adults, scientifically acceptable
Clinical Research - not NIH-defined Phase III Trial

Project Year	Direct Costs Requested	Estimated Total Cost
1	237,968	375,824
2	237,342	374,835
3	237,342	374,835
4	356,013	562,252
5	356,012	562,251
TOTAL	1,424,677	2,249,997

1U01CE003201-01 Silverman, Jay

RESUME AND SUMMARY OF DISCUSSION: This application, by a seasoned Principal Investigator (PI), proposes to conduct a cluster-matched control trial to rigorously evaluate the Close to Home (C2H) model across 14 diverse communities in California through collection and analyses of social network, social media, and community-wide school-based data in order to provide evidence on the effectiveness of a community-level approach to prevent sexual violence (SV). The specific aims are to: 1) develop and implement a cluster-matched control design in partnership with the Research Advisory Board (RAB), including collecting baseline social network and social media data across 14 diverse California communities; 2) evaluate the effects of the C2H model on social norms and community connectivity regarding SV, and SV incidence through longitudinal analyses of baseline and 24-month follow-up social network data; and 3) extend Aim 2 findings through analyses of community-level effects on SV social norms and behavior based on multiple waves of community-level school-based data and geocoded social media data. Reviewers determined this proposal to have high potential for public health impact.

With regard to significance, the proposed project is designed to meet the goals of the Centers for Disease Control and Prevention's (CDC's) Notice of Funding Opportunity (NOFO) in that the intervention to be evaluated is a community mobilization model that engages community members across multiple sectors and is designed to create deep social norms changes and enhance protective community environments. The proposed project's design to evaluate the last two phases of C2H that are scheduled to begin in December 2021 clearly grounds the project in prevention at the community-level. The application does a very nice job of explaining all the elements of C2H, which makes it easy to see how this fits the call of the NOFO. Rather than being a school-based program, C2H is a community-led intervention that touches residents of all ages and backgrounds where they live, work, and learn. The prevention staff will recruit youth from schools, community organizations, through adults in the young people's lives as network support resources. The C2H program is implemented such that teens and adults work together to assess SV in their community. In addition, they engage in a discussion process in which they have deeper conversations about SV. They then build strategies to make social norms change, and then act on and implement the strategies to determine what will work best. The C2H program has been widely used in communities throughout California. This truly is a community-led, community-generated organic program in which community members define what SV looks like for them in their community. This is discussed in the literature as something that should happen, but oftentimes it does not actually occur. Instead, researchers often come in from the outside and impose what that should look like for the community. The proposed study is quite significant in that it is well-designed to capture the experience of communities that are disproportionately impacted by SV, particularly racial and sexual minority communities.

In terms of the investigators, the strengths are very notable, and the division of labor is clearly specified. The team is housed at the University of California, San Diego (UCSD) where they are associated with Center on Gender Equity and Health (GEH) that has relevant experience conducting this type of evaluation research. The researchers are very well-established, and the programs are already being implemented in the study communities. The investigators have worked together previously. Two individuals from the funded Rape Prevention and Education (RPE), the Director and epidemiologist, are included on the team as investigators. This speaks to the notion that there will be a very strong collaboration between the research team at UCSD and the RPE-funded organization that will be implementing C2H. Additionally, access is demonstrated to social networks. Given that social network analysis (SNA) is such a broad field, inclusion of team members with different experience in this area is beneficial. One individual has more expertise in the social media aspect of SNA, while another has expertise in the more traditional self-report survey. This is important since both elements

are proposed in this application. Also, a strong Community Advisory Board (CAB) has been established.

Particularly innovative about the proposed study is that while C2H has been implemented for some time and California has invested heavily in it, there has not been a rigorous evaluation of the program. The application is highly innovative in its use of SNA, which is a novel way to evaluate community-based interventions as primary prevention approaches. Having a strong social network component can help to understand how community-based intervention strategies diffuse across individuals within that community, as well as how programs and policies can shift. This seems quite innovative for the field of violence prevention. It is somewhat novel that social media will be used to try to tap into Twitter accounts to measure and assess community-level social norms around SV. One potential benefit with Twitter is that it is a more open platform that could provide interesting data about whether users are posting about particular topics they are learning about, such as consent and SV, and if or how people are engaging with others who are not in their immediate circle. The approach is interesting in terms of tapping into people's social networks to determine whether the changes will diffuse across different levels (teens, peer groups, schools). Integrating youth and adults as network supports is very important for the proposed study. Twitter is used in the approach in two ways. Individuals who are included in the survey studies will be asked for their Twitter names, and the investigators will go into Twitter using specialized software to download participants' entire profiles. The goal is to get a sense of each person's online Twitter presence in terms of their tweets, hashtags, and networks to determine whether the intervention is having an effect on what an individual post on Twitter. Also, a much more general approach will be utilized in which geocoded Twitter data will be examined, such that every time someone posts on Twitter in specific areas, this can be pinpointed. Standardized measures will be used. The methodology suggests that a diverse variety of participants will be recruited. The clustered nature of the data will be accounted for appropriately. A fairly straightforward differences-in-differences (DID) test is proposed to evaluate the intervention and a sufficient power analysis is provided. The scientific environment is excellent. Very strong letters of support (LOS) are provided that clearly demonstrate solid buy-in and reflect that C2H is being implemented.

Weaknesses also were identified. While the use of Twitter to tap into social norms around SV is innovative, it also is potentially problematic in that it is not clear how much Twitter is going to reflect social norms around SV if large numbers of people are not using Twitter in these marginalized communities. Moreover, teenagers typically do not use Twitter that often. About 35% to 40% of teens 15 to 18 years of age are on Twitter, which is not a majority. Instead, teens are using different social media platforms that are more private for their network of friends. Twitter also is limited in terms of demographics. However, the Twitter limitations appear to be accounted for with the more traditional SNA of having the individuals in the surveys nominate their peers. Regarding the approach, insufficient details are provided about the social context and matched group design. More discussion would have been beneficial on the types of individuals who would be recruited for that network, given that different terminology is clearly needed for different types of individuals within the network. It is not clear whether this will capture the diversity of experiences with SV. There is some concern about the ability to maintain participants over time. While a retention strategy is described, this still may be challenging since this is going to be a long-term process. It is not clear whether an incentive of $30 will be sufficient to entice people back over such a long period of time. Also, access to technology is not sufficiently addressed in that there appears to be an assumption that all participants will have a phone, internet, and computer access. However, this may not be the case in diverse populations. It is extremely important to be mindful of the digital divide in this country among a diverse population, particularly for those who are not digital natives or English speakers. In addition, there is still limited internet access in many rural areas. Although the narrative states that all of the community in which the study will take place have internet access in public places, that does not necessarily mean that people can get to

those public places. Although the team clearly has extensive experience in SNA and data analysis, it would have been beneficial to see more information on the type of analyses proposed and how the data will be utilized. While DID is used a lot in evaluation work, it is not clear why there are no plans to do a lot more with these amazing multi-level longitudinal data.

Protection of human subjects is acceptable. Inclusion is acceptable in terms of gender, minority groups, and age. Animal welfare, biohazards, and dual use research are not applicable. An acceptable resource sharing plan/data management plan (DMP) is provided. Overall, this is an outstanding application that is extremely strong with negligible weaknesses.

DESCRIPTION (provided by applicant): Two approaches promoted in the current RFP and the CDC's STOP SV Strategies guidance are 1) Promoting Social Norms that Protect Against Violence, and 2) Creating Protective Environments. The current proposal to evaluate Close to Home (C2H), a community mobilization SV prevention model, incorporates both approaches within a rigorous and innovative research design. Community mobilization to prevent SV, including the C2H model, is designed to create deep social norms change and enhance protective community environments, yet has rarely been evaluated. We propose to rigorously evaluate the C2H model via a cluster-matched control trial across 14 diverse communities in California via collection and analyses of social network, social media and community-wide school-based data in order to provide much-needed evidence on the effectiveness of this community-level approach to prevent SV. California is uniquely poised to conduct this research given a) the number of communities implementing C2H, b) CDPH's 10-year commitment to building capacity to support local implementation of this model, and c) CDPH's ongoing partnership on RPE evaluation with University of California San Diego's Center on Gender Equity and Health, a center with the highest levels of expertise in evaluation of sexual violence prevention programs, social network science, and assessing norms across social networks and social media. We propose a highly-innovative, multi-level evaluation of C2H involving the following aims: 1) Develop and implement a cluster-matched control design in partnership with the Research Advisory Board, including collecting baseline social network and social media data across 14 diverse California communities; 2) Evaluate effects of the C2H model on social norms and community connectivity regarding SV, and SV incidence via longitudinal analyses of baseline and 24-month follow-up social network data; and 3) Extend Aim 2 findings via analyses of community-level effects on SV social norms and behavior based on multiple waves of community-level school-based data and geocoded social media data. The proposed research will fill a critical evidence gap in SV prevention by evaluating effects of C2H, a promising community mobilization SV prevention model, on community-level social norms, community connectivity (a key element of protective environments), and SV incidence in accordance with CDC's STOP SV guidance. Proposed longitudinal social network analyses will also provide the first evidence regarding diffusion of changes in SV social norms and behaviors. These contributions will accelerate prevention of sexual violence by equipping local, state and national stakeholders with the rigorous and generalizable evidence needed to inform implementation of community-level sexual violence prevention programming.

PUBLIC HEALTH RELEVANCE: Community mobilization to prevent SV, including the C2H model, is designed to create deep social norms change and enhance protective community environments, two approaches specified in the current RFP and the CDC's STOP SV Strategies guidance, yet community mobilization for SV prevention has rarely been evaluated. We propose to rigorously evaluate the C2H model via a multi-level cluster-matched control trial across 14 diverse communities in California via collection and analyses of social network, social media and community-level school-based data in order to provide much-needed evidence on the effectiveness of this community-level approach to prevent SV, including reducing harmful social norms and creating protective environments. The proposed highly-innovative research will fill a critical evidence gap in SV prevention by evaluating effects of a promising

community mobilization SV prevention model on community-level social norms and incidence of multiple forms of SV (sexual assault, in-person and online sexual harassment, stalking), in accordance with CDC's STOP SV guidance.

CRITIQUE 1

Significance:	2
Investigator(s):	1
Innovation:	2
Approach:	2
Environment:	1

Overall Impact:
Strengths

- Research indicates that shifting community-level social norms so that violence becomes socially unacceptable and increasing community cohesion to reduce social barriers and enhance social support to prevent violence can significantly reduce sexual violence (SV). Community mobilization is designed to accomplish these goals.
- The principal investigators have proposed a well-design, rigorous evaluation of the effectiveness of community mobilization to prevent SV, in the form of the Close to Home (C2H) Program. There are numerous strengths of this application.
- C2H is truly a community-level and community-led primary prevention program, which makes it innovative. Too often the voices and perspectives of the target populations have been missing from the research. There is a particular need to include the experiences of diverse populations. The efforts to measure both SV perpetration and victimization in four forms (sexual assault, in-person and online sexual harassment), and stalking also makes the application innovative.
- The investigators have strong support from the California Department of Public Health (CDPH) and seven community-based Rape Prevention and Education (RPE) programs have already initiated implementation of C2H across seven diverse California communities.
- The investigators have a strong, well-established research team that is prepared to conduct the research.

Weaknesses

- No weaknesses are noted.

1. Significance
Strengths

- Analyses of these data will provide evidence at the individual, interpersonal and community level of the effectiveness of this community mobilization approach to reduce SV incidence.
- The application aligns very well to two approaches promoted in the current NOFO and the CDC's STOP SV Strategies: (1) Promoting Social Norms that Protect Against Violence and (2) Creating Protective Environments.
- The proposed project does an excellent job of addressing the outer layers of the ecological model (e.g., making community-level change in social norms that contribute to SV). At the same time, the proposed project will investigate individual and interpersonal-level factors.
- The project is well-designed to capture the experiences of communities that are disproportionately burdened by SV, such as racial/ethnic minorities and sexual and gender minorities. The investigators have a clear plan for gaining access to these communities.

Weaknesses

- No weaknesses are noted.

2. Investigator(s)
Strengths
- The investigators at the University of California, San Diego campus and the Center on Gender Equity and Health have decades of relevant experience and are clearly leaders in their field.
- The investigators have established strong working relationships with rape prevention and education programs (e.g., nine are implementing the Close to Home Program and seven are committed to participating in the proposed research).
- The Principal Investigator has published more than 200 peer-reviewed manuscripts on gender-based violence.
- The other investigators have extensive experience in areas that are vital to the success of the proposed project, including understanding social networks (James Fowler), social norms (Holly Shakaya), and gender-based violence in international populations (Sabrina Boyce)

Weaknesses
- No weaknesses are noted.

3. Innovation
Strengths
- In order to prevent SV, there is a need for research that rigorously investigates risk and protective factors at multiple ecological levels. The investigators have submitted an application that has a clear plan to conduct this type of research. In particular, they have a plan to precisely assess the diffusion of social norms across social networks via social network mapping and analysis.
- The investigators have proposed to use standardize measures, such as the Illinois Rape Myth Acceptance Scale. However, by conducting research that is community-led, the investigators can also tap into important differences in diverse populations. For example, perceptions of SV may vary across social class, racial groups, sexual orientation, and gender identity. Detecting these differences would be a significant contribution to the field.
- The proposal has great promise for advancing the field by focusing on primary prevention and focusing on ways to change community-norms around SV.
- The investigators have clearly articulated how they propose to use the Theory of Change (Theory of Bounded Normative Influence).
- The investigators have described an appropriate evaluation strategy.
- The Component A activities appear to be balanced and well-described.

Weaknesses
- No weaknesses are noted.

4. Approach
Strengths
- The investigators have described a clear methodology to rigorously evaluate the C2H model via a collection and analyses of social network, school-based and social media data.
- The proposed research clearly focuses on Promoting Social Norms that Protect Against Violence and Creates Protective Environments.
- The investigators have described an evaluation plan that includes valid and reliable measures, such as the Illinois Rape Myth Acceptance Scale.

- The investigators have a plan and have demonstrated the ability to recruit and retain an adequate study population.
- The timeline appears to be feasible and sufficiently realistic to complete Component A activities within a 3-year project period and completing Component B activities within a 2-year project period.

Weaknesses

- No weaknesses are noted.

5. Environment

Strengths

- There is strong support for this application. In particular, the CDPH has a ten-year commitment to building capacity to support local implementation of the C2H model. In addition, the CDPH has an ongoing partnership on RPE evaluation with the University of California San Diego's Center on Gender Equity and Health and the investigators. Further, the investigators have identified a Research Advisory Board.
- Based on the scientific environment, it appears that the investigators have a high probability of completing the proposed research.
- The description of the duties, percentage-of-time commitments, and responsibilities of all project personnel includes clear lines of authority and supervisory capacity.

Weaknesses

- No weaknesses are noted.

Protections for Human Subjects
Acceptable Risks and/or Adequate Protection

Data and Safety Monitoring Plan (Applicable for Clinical Trials Only)
Not Applicable

Inclusion of Women, Minorites, and Age (Applicable Only for Human Subjects Research)
G1A=Includes both genders, acceptable
M1A=Includes minorities and non-minorities, acceptable
C6A=Includes children and adults (no older adults), acceptable

Vertebrate Animals
Not Applicable (No Vertebrate Animals)

Biohazards
Not Applicable (No Biohazards)

Dual Use Research of Concern
Not Applicable

Budget and Period of Support
Recommend as Requested

Resource Sharing Plans
Acceptable

Data Management Plan (DMP)
Acceptable

CRITIQUE 2

Significance:	3
Investigator(s):	1
Innovation:	3
Approach:	3
Environment:	1

Overall Impact:
Strengths
- The administrative infrastructure and available resources are strong and well delineated in the proposal.
- There are ties to University of California San Diego's Center on Gender Equity and Health in the proposed research.
- The potential sample is diverse socially and economically.
- Sampling is proposed of a wide variety of communities in California (14 sites, 7 of which are high burden).
- A plausible approach and extensive evaluation strategy are described in the proposal.

Weaknesses
- Retention of the sample is potentially problematic.

1. Significance:
Strengths
- The project directly addresses multiple communities across California.
- Social network data will be collected by participants, allowing for an expanded connection concerning what a "community" means.

Weaknesses
- The sample relies on participants solicited via MTurk, which is a controversial tool of data collection.
- The proposed research includes only students in California.
- The guiding theoretical approach could be better defined.

2. Investigator(s):
Strengths
- The institutional support is good.
- The proposed research is based on an established intervention.
- The research team is diverse geographically and demonstrate a diversity of experience.
- The team has extensive experience with clinical trials and other interventions.

Weaknesses
- No weaknesses are noted.

3. Innovation:
Strengths
- The focus on a potentially wide diversity of responses and participant experiences is a strength.
- Over-time data on the effectiveness is useful for the evaluation of the intervention.

Weaknesses
- The participants are drawn from only those who have internet access and opportunities for privacy.

4. Approach:
Strengths
- The proposed research includes building a Research Advisory Board.
- The staffing pattern is developed.
- The proposed measures and schedule are well defined.

Weaknesses
- The retention of participants is questionable.

5. Environment:
Strengths
- There is a detailed description of the administrative organization for the project.
- The resources and institutional support are clear.
- The letters of support are strong.

Weaknesses
- No weaknesses are noted.

Protections for Human Subjects
Acceptable Risks and/or Adequate Protections
- While risks appear to be minor, there might be an elevated risk associated with participation in surveys about behavior that is by definition potentially harmful especially with those 10-14 years of age.

Data and Safety Monitoring Plan (Applicable for Clinical Trials Only)
Not Applicable

Inclusion of Women, Minorites, and Age (Applicable Only for Human Subjects Research)
G1A=Includes both genders, acceptable
M1A=Includes minorities and non-minorities, acceptable
C6A=Includes children and adults (no older adults), acceptable
- The objectives of the initiative could not be achieved without children including full gender and race/ethnicity representation

Vertebrate Animals
Not Applicable (No Vertebrate Animals)

Biohazards
Not Applicable (No Biohazards)

Dual Use Research of Concern
Not Applicable

Budget and Period of Support
Recommend as Requested

Resource Sharing Plans
Acceptable

Data Management Plan
Acceptable

CRITIQUE 3

Significance:	1
Investigator(s):	1
Innovation:	1
Approach:	2
Environment:	1

Overall Impact
Strengths

- This application, from a strong and organizationally diverse team of investigators, proposes to evaluate the California Department of Public Health's (RPE-funded) Close to Home program, a community-level intervention designed to reduce sexual violence. The proposal is responsive to two of the three strategies listed in the NOFO: Promote Social Norms that Protect Against Violence and Create Protective Environments.
- Close to Home is one of the few approaches to SV prevention that targets social norms and social structures, but it has not yet been evaluated. The project would assess three different socio-ecological levels (individual, interpersonal, and community).
- The research design elements described in the approach are generally in line with the stated project objectives. All of the survey instruments proposed are supported by evidence attesting they are valid and reliable measures of their respective constructs, including SV perpetration and victimization.
- Inclusion of the CDPH RPE director and another central CDPH staff member should support collaborative project leadership and direction.

Weaknesses

- It is not clear if the participant retention strategies are rigorous and incentive-laden enough to achieve the retention targets.
- Details on the data analyses were a bit sparse. For example, it is not entirely clear how the matched cluster evaluation data (Component A) will be analyzed. The power analysis suggests it will be some type of clustered analysis comparing perpetration rates, which is good, but no other details are provided on how this crucial analysis will be conducted. Some components of the social network analyses are also unclear.

1. Significance
Strengths

- This application proposes to evaluate the California Department of Public Health's (RPE-funded) Close to Home program, a community-level intervention designed to reduce sexual violence.
- The proposal is responsive to two of the three strategies listed in the NOFO: Promote Social Norms that Protect Against Violence and Create Protective Environments.
- The background and current implementation of Close to Home is well-described.
- Close to Home is currently being targeted to populations that experience a greater burden of SV, and the proposed project will evaluate those implementations.

Weaknesses

- No weaknesses are noted.

2. Investigator(s)
Strengths

- Dr. Silverman has a well-established track record of successfully completing federally funded projects aimed at understanding and reducing SV, including several evaluation studies.

- Drs. Shakya and Fowler are experts on social network analysis and will provide that needed expertise to collect and analyze the data social network data from various sources.
- Ms. Boyce has experience leading collaborations between UCSD and RPE-funded organizations.
- Ms. Bertoldo, who is the RPE Director, and Ms. White, who is an epidemiologist, are both with the CDPH Injury and Violence Prevention Branch. Their inclusion as investigators on the project should ensure strong communication across research and implantation activities.

Weaknesses

- No weaknesses are noted.

3. Innovation

Strengths

- Close to Home is one of the few approaches to SV prevention that targets social norms and social structures, but it has not yet been evaluated.
- The project would assess three different socio-ecological levels (individual, interpersonal, and community).
- The proposal includes a social network analysis approach to understand diffusion of information across networks, which is quite novel for this area and would move the field forward both substantively and methodologically.

Weaknesses

- Although the social network data collection approach (via Twitter) is innovative, the relevance of those data may be largely restricted to certain demographics because, even among active users, most rarely post on the platform.

4. Approach

Strengths

- The research design elements described in the approach are generally in line with the stated project objectives. Social network data will be collected in multiple ways across the project, peer nomination, self-reported Twitter ID, and general Twitter API access. This will provide a tremendous amount of additional data on peer influences and both interpersonal and community diffusion of intervention information.
- All of the survey instruments proposed are supported by evidence attesting they are valid and reliable measures of their respective constructs, including SV perpetration and victimization.
- Both components of proposed study seem feasible given the respective timelines.

Weaknesses

- It is not clear if the participant retention rates needed to support the proposed analyses can be reached given the proposed retention plan.
- Details on the data analyses were a bit sparse. For example, it is not entirely clear how the matched cluster evaluation data (Component A) will be analyzed. The power analysis suggests it will be some type of clustered analysis comparing perpetration rates, which is good, but no other details are provided on how this crucial analysis will be conducted.
- It is not clear why simple difference-of-difference tests are proposed in cases where there will be four repeated assessments within individuals and a large overall sample.

5. Environment

Strengths

- UCSD and CDPH are supportive environments for this work, given the investigators past accomplishments and the strong letters of support.
- Inclusion of the CDPH RPE director and another central CDPH staff member should support collaborative project leadership and direction.

- The division of labor is articulated within the research strategy.

Weaknesses

- No weaknesses are noted.

Protections for Human Subjects
Acceptable Risks and/or Adequate Protection

Data and Safety Monitoring Plan (Applicable for Clinical Trials Only)
Not Applicable

Inclusion of Women, Minorites, and Age (Applicable Only for Human Subjects Research)
G1A=Includes both genders, acceptable
M1A=Includes minorities and non-minorities, acceptable
C6A=Includes children and adults (no older adults), acceptable

Vertebrate Animals
Not Applicable (No Vertebrate Animals)

Biohazards
Not Applicable (No Biohazards)

Dual Use Research of Concern
Not Applicable

Budget and Period of Support
Recommend as Requested

Resource Sharing Plans
Acceptable

Data Management Plan (DMP)
Acceptable

THE FOLLOWING SECTIONS WERE PREPARED BY THE SCIENTIFIC REVIEW OFFICER TO SUMMARIZE THE OUTCOME OF DISCUSSIONS OF THE REVIEW COMMITTEE, OR REVIEWERS' WRITTEN CRITIQUES, ON THE FOLLOWING ISSUES:

PROTECTION OF HUMAN SUBJECTS: ACCEPTABLE

INCLUSION OF WOMEN PLAN: ACCEPTABLE
Includes both genders and is scientifically acceptable.

INCLUSION OF MINORITIES PLAN: ACCEPTABLE
Includes minorities and non-minorities and is scientifically acceptable.

INCLUSION OF AGE PLAN: ACCEPTABLE
Includes children and adults (no older adults) and is scientifically acceptable.

COMMITTEE BUDGET RECOMMENDATIONS: The budget was recommended as requested.

COMMITTEE RESOURCE SHARING/DATA MANAGEMENT PLAN RECOMMENDATIONS:
The resource sharing/data management plans are acceptable.

MEETING ROSTER

National Center for Injury Prevention Special Emphasis Panel
NATIONAL CENTER FOR INJURY PREVENTION AND CONTROL
CE20-001 - Evaluating Practice-based Programs, Policies, and Practices from CDC's Rape Prevention Education Program
ZCE1 KFJ (02)
04/29/2020 - 04/30/2020

CHAIRPERSON(S)

JENNINGS, WESLEY, PHD
CHAIR & PROFESSOR
UNIVERSITY OF MISSISSIPPI
UNIVERSITY, MS 38677

MEMBERS

CARES, ALISON C, PHD
ASSOCIATE PROFESSOR OF SOCIOLOGY
UNIVERSITY OF CENTRAL FLORIDA
ORLANDO, FL 32816

DE LA RUE, LISA, PHD
ASSISTANT PROFESSOR
UNIVERSITY OF SAN FRANCISCO
SAN FRANCISCO, CA 94117

ECKHARDT, CHRISTOPHER I, PHD
PROFESSOR & DIRECTOR OF CLINICAL TRAINING
DEPARTMENT OF PSYCHOLOGICAL SCIENCES
PURDUE UNIVERSITY
WEST LAFAYETTE, IN 47907

HOLTZMAN, MELLISA K, PHD
PROFESSOR
DEPARTMENT OF SOCIOLOGY
BALL STATE UNIVERSITY
MUNCIE, IN 47306

JOZKOWSKI, KRISTEN N, MS, PHD
PROFESSOR
INDIANA UNIVERSITY
BLOOMINGTON, IN 47405

PESKIN, MELISSA, PHD
ASSOCIATE PROFESSOR
UNIVERSITY OF TEXAS HEALTH SCIENCE CENTER AT
HOUSTON-SCHOOL OF PUBLIC HEALTH
HOUSTON, TX 77030

SAMP, JENNIFER A., PHD
PROFESSOR
UNIVERSITY OF GEORGIA
ATHENS, GA 30602

SHOREY, RYAN CHRISTOPHER, PHD
ASSISTANT PROFESSOR
DEPARTMENT OF PSYCHOLOGY
UNIVERSITY OF WISCONSIN-MILWAUKEE
MILWAUKEE, WI 53211

SMITH, CARLY P, PHD
ASSISTANT PROFESSOR
PENNSYLVANIA STATE UNIVERSITY
COLLEGE OF MEDICINE
HERSHEY, PA 17033

SWARTOUT, KEVIN MICHAEL, PHD
ASST. PROFESSOR
GEORGIA STATE UNIVERSITY
ATLANTA 30302

WEST, CAROLYN M, PHD
ASSOCIATE PROFESSOR
UNIVERSITY OF WASHINGTON
TACOMA, WA 98402

SCIENTIFIC REVIEW OFFICER

LEEKS, KIMBERLY D, PHD, MPH
SCIENTIFIC REVIEW OFFICER
NATIONAL CENTER FOR INJURY PREVENTION & CONTROL
NATIONAL CENTER FOR ENVIORNMENTAL HEALTH/ATSDR
EXTRAMURAL RESEARCH PROGRAM OPERATIONS
CENTERS FOR DISEASE CONTROL AND PREVENTION
ATLANTA, GA 30341

EXTRAMURAL SUPPORT ASSISTANT

JACKSON, LATANYA D
GRANTS TECHNICAL ASSISTANT
NATIONAL CENTER FOR INJURY PREVENTION & CONTROL
NATIONAL CENTER FOR ENVIRONMENTAL HEALTH/ATSDR
EXTRAMURAL RESEARCH PROGRAM OPERATIONS
CENTERS FOR DISEASE CONTROL & PREVENTION
ATLANTA, GA 30341

PROGRAM REPRESENTATIVE

NEURATH, SUSAN, PHD
SCIENTIFIC PROGRAM OFFICER
NATIONAL CENTER FOR INJURY PREVENTION & CONTROL
NATIONAL CENTER FOR ENVIRONMENTAL HEALTH/ATSDR
EXTRAMURAL RESEARCH PROGRAM OPERATIONS
CENTERS FOR DISEASE CONTROL AND PREVENTION
ATLANTA, GA 30341

Consultants are required to absent themselves from the room during the review of any application if their presence would constitute or appear to constitute a conflict of interest.

CE20-001 - Evaluating Practice-based Programs, Policies, and Practices from CDC's Rape Prevention Education Program

Evaluation of the Close to Home Program in California: Assessing the impact of community mobilization to prevent sexual violence at the individual, social network, and community levels

PI: Jay G. Silverman

1U01CE003201-01

Response to Weaknesses Identified by Reviewers

We are very grateful for the valuable feedback provided by reviewers of our grant application and appreciate the opportunity to consider the identified weaknesses and improve our study design. This document contains a point-by-point response to weaknesses identified by the panel who reviewed this grant proposal. Below, comments from reviewers are numbered and italicized and our research team's response follows in text with normal formatting.

1. *While the use of Twitter to tap into social norms around SV is innovative, it also is potentially problematic in that it is not clear how much Twitter is going to reflect social norms around SV if large numbers of people are not using Twitter in these marginalized communities. Moreover, teenagers typically do not use Twitter that often. About 35% to 40% of teens 15 to 18 years of age are on Twitter, which is not a majority. Instead, teens are using different social media platforms that are more private for their network of friends. Twitter also is limited in terms of demographics. However, the Twitter limitations appear to be accounted for with the more traditional SNA of having the individuals in the surveys nominate their peers*.

We would like to fully acknowledge that our proposed use of Twitter data is not intended to be a representative sample of the local communities in which the data collection will be taking place, nor do we intend to capture a youth sample with these data. Pew Research Center has documented that Twitter users in the US tend to be young adults (44% of 18-24 year olds in the US use Twitter), individuals with post-secondary education, with higher income, and who lean

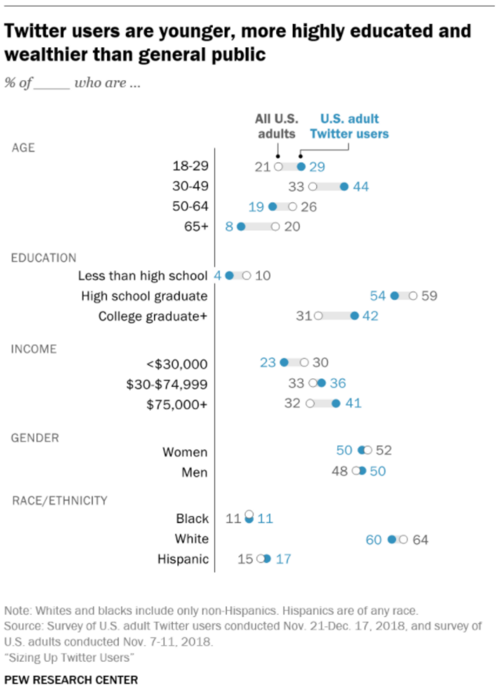

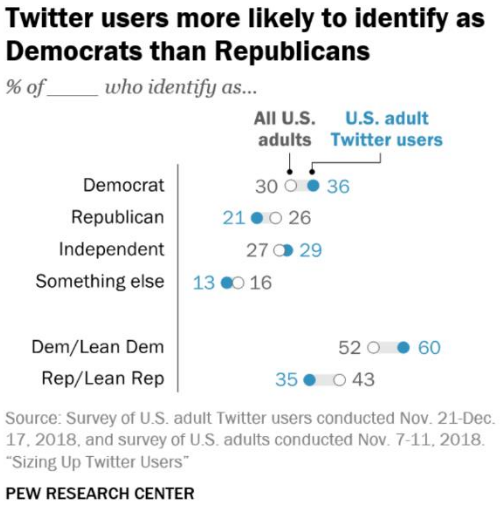

democratic in their political beliefs. We also know that only about 22% of the US population uses Twitter, only about 20% of those actively post on Twitter, and only 2-3% have geocoding attributed to their posts. These data provide only a snapshot of the adult population in our communities of interest, yet they offer a very unique ability to observe online expression of opinions, attitudes, and behaviors that could not be collected via typical survey data collection methods.

Despite these limitations of the data in representativeness of the population we are studying, Twitter data is a highly innovative and a helpful data source to learn about social norms and social commentary related to sexual violence prevention. Despite the "80/20 rule" of social media, in which only about 20% of users actively post commentary and 80% are silent observers, these 20% are the voices that create the social environment for those 80% who are "onlookers". They therefore likely represent opinion leaders and their posted comments likely help to shape social norms. Because of our deep interest in understanding social norms change as a result of the Close to Home (C2H) intervention, Twitter data, while not perfect, is a useful data source to better understand trends of some social norms that exist in these communities.

To address some of these limitations of Twitter data, we will ensure that we utilize analytical techniques to better understand the representativeness of these Twitter data and to account for the "80/20 rule" characteristics of the data. First, to get a better understanding of representativeness, we can use Twitter's ad creation platform to estimate the number of individuals from our communities of interest are using Twitter and compare this to census estimates of the population size. From what we see so far, users make up anywhere from 20-40% of the communities. The ability to assess representativeness is severely limited by the lack of demographic information publicly available for Twitter users. Second, and more robustly, we will compare the distribution of geocoded Twitter data to the larger pool of Twitter data to evaluate the representativeness of the geocoded sample to the larger Twitter user population. Presentation of the results we develop from Twitter data will include these assessments of representativeness so that it is abundantly clear that results from the Twitter data are not representative of the study population but provide an important signal of how social norms may be shifting over time and in response to the C2H intervention. To address the 80/20 rule found in social media data, we will use hierarchical analyses with fixed effects for individual users to allow for detection of individual-level change in Twitter posts from pre to post-intervention. Such hierarchical models will help ensure that we are able to see how individual opinion leader's comments change over time and that they are not lost within the population averages other statistical models provide.

2. *Regarding the approach, insufficient details are provided about the social context and matched group design. More discussion would have been beneficial on the types of individuals who would be recruited for that network, given that different terminology is clearly needed for different types of individuals within the network. It is not clear whether this will capture the diversity of experiences with SV. (E.2.1 Selection of matched control communities)*

Three levels of individuals will be recruited as part of the social network sampling: 1) C2H participants (i.e., seed egos), 2) six peers nominated by each of the seed egos (i.e., first tier alters), 3) three peers nominated by each of the first tier alters (i.e., second tier alters). For all nominated alters, the aim is for participants to nominate influential individuals within their social networks with whom they would likely share ideas/information/beliefs that are important to them, including things that they have learned from participating in C2H. We will engage in a rigorous process during formative research (Year 1) of identifying the best wording of questions to get participants to nominate these types of network members (i.e., name generator questions). A small sample of name generator questions that will likely be tested as part of our formative research includes:

- "With whom do you spend free time?"
- "With whom do you discuss important matters?"
- "With whom do you discuss something personal and private?"

General inclusion criteria for all participants is that they are age 14-24 years, live in the geographic community of a C2H/control program (defined by zip codes), and that they are willing and able to provide informed consent.

The objective of the social network sample is to observe how the impact of the C2H program is (or is not) diffusing throughout the social networks of participants. The design for sampling this social network will allow us to assess this, as well as other ways in which social network members may be influencing one another in terms of beliefs and behavior. We estimate that we will recruit a sample of approximately 2866 youth, which is highly likely to capture a wide range of experiences with the primary and secondary outcomes of interest and that variability in the wide range of sexual violence outcomes we plan to measure will not limit our ability to conduct complex social network analyses. This sample, however, is not meant to provide a representative sample of sexual violence experiences in the population, as would be the aim if we were to be conducting only an observational study on the prevalence of sexual violence. As a reference point, the Youth Risk Behavior Survey has estimated the following prevalences of sexual violence in California in 2019. Neither SV prevalence is so rare that the prevalence in our proposed sample of 2866 youth will limit our ability to assess impact of the C2H program; using these YRBS estimates, we would estimate 235 study participants to report physically forced sexual intercourse and 550 to report sexual violence. We have no reason to believe that our proposed sample will be higher or lower risk for sexual violence than this school-based YRBS sample.

California, High School Youth Risk Behavior Survey, 2019*

	Total	Female	Male
Were ever physically forced to have sexual intercourse (when they did not want to)	8.2% (6.9–9.8)<br><br>n=1313	11.3% (9.6–13.2)<br><br>n=652	5.2% (3.3–8.1)<br><br>n=654
Experienced sexual violence by anyone (including kissing, touching, or being physically forced to have sexual intercourse when they did not want to, one or more times during the 12 months before the survey)	19.2% (10.2–33.2)<br>n=1332	22.1% (14.6–31.9)<br>n=660	16.3% (6.1–36.6)<br>n=665

*The Centers for Disease Control and Prevention. 2019. “YRBSS Results: Youth Online High School Results.” Accessed 10/26/2020 at: https://www.cdc.gov/healthyyouth/data/yrbs/results.htm

The reviewers also raised concern about the level of detail provided on the way we would conduct our matched group design and we would like to provide additional details of our plan. Intervention (C2H) communities will be clearly defined with each local CDPH-funded organization by zip code and the matching to control communities will be conducted at the zip code level. The proposed control program is available in nearly every county in the state, so there is a large pool of zip codes from which the control communities can be selected. Inclusion criteria for creating the potential “pool” of control communities were selected to avoid contamination/spill-over effects, ensure access to necessary data, and to do our best at creating equivalency between underlying, unmeasured characteristics of control and intervention communities. The inclusion criteria for control communities included in the matching pool are that the zip codes are:

- **Avoiding contamination:** not within 20 miles of intervention community, does not currently have a CDPH-funded RPE program being implemented in it, does not have a community mobilization program being implemented in it.
- **Ensuring access to data:** the schools within the zip code have recent history of collecting CHKS survey data
- **Equivalency with C2H communities:** has rape crisis center within the county, has at least 1 high school in the zip code

Once our pool of potential control zip codes is determined, we will use propensity score matching to identify three communities that best match each of the C2H communities. As stated in the proposal, we will match communities on community-level factors that are theorized to be most closely related to SV and that are readily available via public data sources, including racial/ethnic composition, age distribution, poverty level, setting type (urban/rural/semi-urban), and violent crime rates. We will first check to make sure that the distribution of each variable we use for matching in the intervention communities is included in the distribution of these variables in the control pool. Then, using logistic regression we will regress treatment status (C2H vs. non-C2H) on selected community-level matching factors. This model will provide us with an estimated probability of being a treatment community, regardless of whether, in reality, they are a C2H or non-C2H community. For each C2H community, we will use their estimated probability of treatment to select non-C2H communities with the closest estimated probabilities of treatment, using optimal, nearest neighbor matching (i.e., when multiple zip codes are equally as close choose one at random). We will then try to recruit the control program implementing organization located in the “nearest neighbor” zip code match for each C2H community. If they refuse, we will try to recruit the next nearest neighbor until recruitment is successful. This matching approach will be accounted for in our analyses by including the propensity scores of the communities as a weight in the analytical models.

3. *There is some concern about the ability to maintain participants over time. While a retention strategy is described, this still may be challenging since this is going to be a long-term process. It is not clear whether an incentive of $30 will be sufficient to entice people back over such a long period of time.*

In response to this identified weakness, we would like to first acknowledge the current controversy among adolescent researchers around the use of monetary incentives. In our experience, a fine balance is needed between increasing incentives to entice youth participation in research and being sensitive to the coercive power high monetary incentives can have on youth. The amount we selected for youth participant incentives, $20 for baseline participation and $30 for follow-up participation, is based on the following considerations: minimum wage in California will be $13 per hour at the time of baseline data collection, the survey is estimated to take about 30-45 minutes of their time, the survey can be completed online, so transportation time is not needed for most participants (see #4 for details on supporting those that do need transportation), device and internet costs needed to complete the online survey.

We do acknowledge that retention of youth participants may be difficult and thorough planning to facilitate strong retention is needed. As such, we plan to further refine our current retention plan collaboratively via ongoing discussions with the proposed RAB and will develop a separate youth research advisory board in order to elicit their perspective on what would help retain participant interest in completing the study. The RAB will consist of 10 C2H participant youth who we will meet with 3 times during Year 1 and once a year as needed as the study continues. Youth RAB members will receive compensation for their time in the form of a $20 gift card per meeting. Based on previous research experience with retaining youth samples in CA, the initial ideas we will propose to the adult and youth RAB to elicit their feedback and additional ideas include:

- Offer graduated incentive amounts with completion of follow-up survey (as proposed)
- Keep up-to-date contact information every 6 months by sending youth-friendly text messages and phone calls (as proposed)

- Utilize peer connections within the social network to encourage participation (e.g., pre-written text-based check-ins that the nominating participant could send to their nominated peer to encourage participation. For example, the text might say "Hey, have you done your follow-up survey yet? I just did mine and got my $30. Cha-ching!")
- Facilitate remote access to participating in the follow-up survey in case a participant has moved away from the region by the time of follow-up data collection
- Intervention and control organizations provide regular community-wide updates, including on social media platforms, on study progress to strengthen awareness of the study in the community

All ideas above and those provided by RAB members would be discussed and prioritized according to potential effectiveness and feasibility.

4. *Also, access to technology is not sufficiently addressed in that there appears to be an assumption that all participants will have a phone, internet, and computer access. However, this may not be the case in diverse populations. It is extremely important to be mindful of the digital divide in this country among a diverse population, particularly for those who are not digital natives or English speakers. In addition, there is still limited internet access in many rural areas. Although the narrative states that all of the community in which the study will take place have internet access in public places, that does not necessarily mean that people can get to those public places*.

The digital divide and access to technology are critical issues in creating an equitable and inclusive participant experience, as well as reducing selection bias in our sample. Also, they are also not easy issues to completely solve. During Year 1 of the project and in future years as needed, we will work closely with adult and youth RAB members and their knowledge of their community and youth members to come up with strategies for minimizing barriers to participation related to technology access.

Currently, we know that most of our C2H program participants are able to access C2H activities via phone or computer, so seed egos will likely be able to access study surveys in the same way that they have been engaging with C2H program meetings during Covid-related restrictions (primarily via zoom). Similarly, we have heard from the C2H program staff that due to the way society and schools in particular have shifted to remote and virtual connection during the Covid-19 epidemic, the technology divide for students/youth is currently being reduced in large part via school-based programs. We do not know, however, how long these technology programs will last and if the devices and internet youth are currently able to use will still be available in future years of the study, but we do expect that the comfort and skills in using these devices will be stronger as a result of Covid-related virtual connection.

To address this concern, we will plan to have multiple ways of completing the survey: 1) online with a personal device, 2) online at a community-location using a device and internet available there (likely program-implementing organization or a library) for which transportation reimbursement would be provided, 3) over the phone orally with a research assistant with reimbursement provided for cost of call, as needed. We will expand this list of options in our discussions with the RAB members and prioritize those approaches that will make study participation feasible for as many people as possible.

We will also consciously consider throughout the planning process and implementation the diverse population that we seek to include in our study. We will do this by 1) discussing with the RAB the diversity of the communities and strategies to make the study more inclusive for all, 2) utilizing measures in our survey instruments that have been validated for use with a wide range of race/ethnicities, LGBTQ populations (including non-heterosexual relationships), rural and urban populations, and youth and young adults, 3) making study instruments available in the primary languages found in the study communities, as well as making an oral version of the survey available for those with limited literacy. We look forward to learning more from our RAB of how we can expand these efforts.

5. *Although the team clearly has extensive experience in SNA and data analysis, it would have been beneficial to see more information on the type of analyses proposed and how the data will be*

utilized. While DID is used a lot in evaluation work, it is not clear why there are no plans to do a lot more with these amazing multi-level longitudinal data.

We are happy to provide more detail about some of the planned analyses for the data collected in this study, which were excluded due to length constraints of the proposal. We also are confidence there will be many additional analyses that we explore once the data is available and more is known about its structure.

Main effects: Main effects will be assessed using difference in difference hierarchical models that include time, treatment condition, and a time-by-treatment interaction and accounts for clustering at the community-level and is adjusted for associated covariates using all three data sources. For models involving social network data, they will include dummy variables to indicate if the participant is a seed ego (reference category), first tier alter or second tier alter to allow us to see how far into the social network impact of the intervention has spread. If we are finding in the data strong differences between treatment and control communities at baseline, we will explore using an instrumental variable approach. If we have high levels of loss to follow-up (>20%), we will consider using an inverse probability of censoring weighting (IPCW) approach to account for these missing data.

Social network diffusion effects: To assess diffusion effects, data would be set up into alter-ego pairs (dyads) with alter outcomes at T2 as the independent variables and ego outcomes at T2 as the dependent variable. Hierarchical models clustering on ego dyads and controlling for baseline outcome values and demographic characteristics would be constructed, assuming that we will only have one observation per alter. We would then do a series of sensitivity analysis to refine our models. Based on subgroup analyses, we will identify potential moderators of these analyses and run the original models again with these interaction terms.

Some examples of additional analyses we will conduct: Both with baseline data and with follow-up data, we plan to explore combining together the social network data with the CHKS data to conduct hierarchical models in which we can consider both individual and community or school-level independent variables in the same model. We will also explore the social network data in extensive detail to better understand the network itself, including assessing how norms/attitudes/behaviors cluster among socially connected individuals, in what types of relationships, how these common characteristics may change over time.

CE20-001 - Evaluating Practice-based Programs, Policies, and Practices from CDC's Rape Prevention Education Program

Evaluation of the Close to Home Program in California: Assessing the impact of community mobilization to prevent sexual violence at the individual, social network, and community levels
PI: Jay G. Silverman
1U01CE003201-01

Covid-19 Proposal Modifications

Since submitting our proposal for the CDC funding opportunity (CE20-001), much has changed in society due to the Covid-19 pandemic and ongoing restrictions to reduce the spread of the virus. During the first year of the funding period, we anticipate that the Covid-19 restrictions will remain in place and will impact our proposed activities. We anticipate that by Fall 2021 (the beginning of Year 2 of the funding period), a Covid-19 vaccine will be available and many of the current societal restrictions will be lifted in California. In this document, we would like to propose the following ways in which we will modify our proposed activities for Year 1 to accommodate current Covid-19 restrictions in California.

1. ***Year 1 Research Advisory Board (RAB) Meetings and Site Visits:***
   - **RAB:**
     All RAB meetings will now be hosted remotely via Zoom (access currently available through UCSD Health Sciences) during Year 1 of the funding period. We anticipate that RAB members will likely be amenable to this modification and have the capacity to join virtual meetings based on the fact that currently, Close to Home (C2H) technical assistant (TA) providers and California Department of Public Health (CDPH) RPE staff have been able to maintain regular meetings with program staff via online video-conferencing with no major limitations. We anticipate, though, that facilitating relationship building within the RAB will be more challenging than if meetings were in-person. With this in mind, relationship building will be prioritized at every meeting and we will schedule meetings to be shorter but more frequent in the beginning months (1.5 hour-long meetings monthly for the first 3-4 months) to create momentum in our rapport and relationship building efforts. Each meeting will have designate time for ice breakers that allow for personal connection that may otherwise happen in between session during an in-person meeting. We will also create more time and space for discussion and feedback so voices from all members are welcomed and able to be heard.
   - **C2H Site Visits:**
     While we planned to perform in-person site visits with all C2H programs, we anticipate that interaction with and observation of C2H programs and participants to be limited under current Covid-19 restrictions. Nonetheless, virtual site-visits can be performed and will help to build relationships between the research team and each C2H organization, as well as four control organizations, and help tremendously in the research team's understanding of the C2H programs and protocols. In all cases, we will set up at least two virtual meetings with program staff to learn more about implementation of the program at that site, to review key documents, and to brainstorm with staff how to best implement study protocols in this setting. These meetings will also include the following, as appropriate:
       - Discussion about the status of their C2H implementation, activities, participants, and modifications due to Covid-19 restrictions.
       - Document review of program plans, products, and calendar of planned activities
       - Step-by-step review of study protocols with staff to understand their acceptability and feasibility in the current structure and activities of this C2H program

- Discussions with staff about technology access for study participants and plan to accommodate needs of participants

Additionally, we also proposed to conduct C2H program observations. Our preferred alternative to in-person program observations will be to see if there is opportunity to participate in C2H program activities/meetings that are held virtually. We have learned from C2H TA providers that many of the adult C2H groups are still meeting regularly on virtual platforms, allowing for an easy, possibly less disruptive way for the research staff to "attend" these meetings. Some organizations are also holding regular virtual meetings with youth C2H groups, for which we would request to do the same, while others are seeking alternative ways to engage their youth teams. If observation of some C2H groups is not possible, additional virtual meetings will be arranged with those organizations' staff to learn more, specifically, about the this component of their program and research staff will review any publicly online interactions among C2H participants (e.g., social media campaigns, website development, etc.). Based on these virtual site visits and RAB meetings, we will update proposed study protocols to accommodate the identified practical constraints and challenges to implementation.

2. ***Pilot testing study instruments:***

We plan to conduct pilot testing of study instruments in Spring 2021. We have modified our plan so that these pilot tests will be conducted virtually. We originally proposed for the majority of survey administration to be conducted virtually; the only exception was that we planned for seed egos (i.e., youth C2H/control program participants) to receive an in-person orientation to the study and to the nomination process of their social network members (i.e., alters). For pilot testing under Covid-19 restrictions, we will plan to virtually pilot test this portion that was planned to be conducted in-person. The research staff will meet with pilot test participants (n=10) via Zoom to provide the orientation to the study and alter recruitment, facilitate their completion of the pilot survey instruments online, and then host a "talk-back" session via Zoom immediately after. While pilot testing itself will be changed from an in-person experience to a virtual one, very little will change in our original plan for data collection whether or not Covid-19 restriction remain. In many ways, by conducting the pilot testing virtually, it will help the research team prepare for the "worst case scenario" in which Covid-19 restrictions are still present during data collection in Fall 2021. If Covid-19 restrictions are rolled back by Fall 2021, we will re-assess if a small amount of additional pilot testing of in-person protocols with seed egos is needed before beginning data collection.

One important consideration for data collection in Fall 2021 is the disruption Covid-19 restrictions may have had on peer relationships. Generally, we know there can be a great deal of fluidity in adolescents' relationships, with relationships sometimes shifting based on the time of year (e.g., summer vs. school year) and shifts in involvement in activities/social settings (e.g., sports teams, changes in where one is attending school, changes in classes). Covid-19 may have dramatically changed who adolescents are able to see and interact with regularly, likely limiting their social networks, and has likely shifted most peer communication to virtual platforms (e.g., social media, text messaging). This has very important implications for our social network sampling protocols. By pilot testing the social network recruitment process during Covid-19 restrictions, we will have our best opportunity to troubleshoot and plan for a "worst case scenario" for peer network recruitment while social networks are limited by Covid-19 restrictions, which is likely more limited than they may be in the fall.

To supplement pilot testing, we will also develop a youth research advisory board to discuss the implications of Covid-19 restrictions on youth relationships and brainstorm additional strategies for enhancing recruitment and retention of youth participants. For example, we will utilize the expertise of the youth RAB members to help us further develop the peer name generator questions so that they stimulate participants to think of a wide group of influential peers, yet also peers with whom they have more stable relationships. Additionally, we imagine that we will likely need to develop with the youth RAB more materials and training on how to recruit peers virtually, such as additional text message templates for recruitment and providing prompts for them to share motivational messages for

participation in the study. We plan to rely on the expertise of youth RAB members about their peers to carefully develop and prioritize the most acceptable and feasible strategies for ensuring strong recruitment and retention when data collection begins. These strategies will be integrated into our pilot testing protocols to allow us to learn from their implementation and continue to improve them before data collection.

***3. California Healthy Kids Survey Administration*:**

By Fall 2021, administration of the California Healthy Kids Survey (CHKS) should be fully functioning as normal, causing no need for modification to the proposed use of CHKS data. During the 2020/21 school year (year 1 of the funding period), however, we do anticipate schools to be highly overburdened and communication regarding plans to administer the CHKS survey with school administrators may be challenging. We will take guidance from RAB members and WestEd staff (the agency that administers CHKS) on when will be the most appropriate time to contact school administrators about the inclusion of the additional CHKS module in their planned administration of CHKS. We anticipate that this likely would be in late spring but will begin conversations early with RAB members and WestEd staff to be sure we plan appropriately.

Conclusion

With these modifications, we hope to accommodate the ways in which Covid-19 may impact our planned study activities. We understand, though, that this is a situation in constant flux that has been unpredictable and unprecedented from the start; we will need to continually monitor the situation and continue to modify our proposed activities accordingly as the funding period progresses.
