# Supplementary material for: Evaluation of 24-Month Effects of the Close to Home Program on Youth Sexual and Dating Violence Across 22 Communities in California: Protocol for a Quasi-Experimental Cluster-Matched Control Trial
Source: JMIR Res Protoc. 2026 Jul 15;15:e81249. doi: 10.2196/81249 (PMC13372292; doi:10.2196/81249)
Supplement: Peer Review Report 2 — Peer review report from the Centers for Disease Control and Prevention of the US Department of Health and Human Services [file resprot-v15-e81249-s006.pdf]

SUMMARY STATEMENT-DRAFT

PROGRAM CONTACT: (Privileged Communication) *Created on:* 06/13/2023 06:46 AM

Application Number: 3 U01 CE003201-04S1
Formerly: 6U01CE003201-02M002

Principal Investigator

SILVERMAN, JAY G

Applicant Organization: UNIVERSITY OF CALIFORNIA, SAN DIEGO

Review Group: ZCE1 TQD (01)
National Center for Injury Prevention Special Emphasis Panel
CE20-001: Evaluating Practice-based Programs, Policies, and Practices from CDCs Rape Prevention and Education (RPE) Program: Expanding the Evidence to Prevent Sexual Violence

Meeting Date: 02/07/2023 *RFA/PA:* CE20-001
Council: MAY 2023
Requested Start: 09/30/2023

Project Title: Evaluation of the Close to Home Program in California: Assessing the impact of community mobilization to prevent sexual violence at the individual, social network, and community levels
SRG Action: Impact Score:24

Human Subjects: 30-Human subjects involved - Certified, no SRG concerns
Animal Subjects: 10-No live vertebrate animals involved for competing appl.
Gender: 1A-Both genders, scientifically acceptable
Minority: 1A-Minorities and non-minorities, scientifically acceptable
Age: 6A-Children and Adults, scientifically acceptable
Clinical Research - not NIH-defined Phase III Trial

Project Year	Direct Costs Requested	Estimated Total Cost
4	358,755	564,658
5	356,013	560,342
TOTAL	714,768	1,125,000

3U01CE003201-04S1 Silverman, Jay

RESUME AND SUMMARY OF DISCUSSION: This application, by a seasoned Principal Investigator (PI), proposes to rigorously evaluate the Close to Home (C2H) intervention. C2H is a promising community-level mobilization sexual violence (SV) prevention model designed to engage members of the community to create social norm changes and enhance protective community environments. The specific aims are to: 1) develop and implement a cluster-matched control design in partnership with the established Research Advisory Board (RAB), including collecting baseline social network and social media data across 22 diverse California communities; 2) evaluate the effects of the C2H model on social norms and community connectivity regarding SV, and SV incidence via longitudinal analyses of baseline and 24-month follow-up social network data; and 3) extend Aim 2 findings via analyses of community-level effects on SV social norms and behavior based on multiple waves of community-level school-based data and geocoded social media data. Reviewers determined this proposal to have high potential for public health impact.

Regarding strengths, this is a highly detailed application overall that reviewers likened to a master class in grantsmanship and how to structure a great scientific argument. In terms of significance, there is a critical gap in the literature about community-focused prevention models in terms of SV. The proposed work represents a true community-level project and evaluation plan. C2H is a community-led intervention that aims to influence social norms by recruiting youth to spread messages throughout their social networks. The study also aims to include the experiences of communities that are disproportionately impacted by SV, particularly racial and sexual minority communities. The proposed work will target three levels of the Social Ecological Model (SEM) in terms of community norms; social networking interpersonal norms; and individual attitudes, intentions, and behaviors. The last two phases of C2H began in late 2021, so the proposed study is aimed toward a pre-/post-examination of those phases. Enhanced assessment materials are included to measure unique forms of SV victimization experienced by LGBTQ+ (lesbian, gay, bisexual, transgender, queer/questioning) and other sexual minority individuals. During the first phase of this work, the RAB and Youth Advisory Board (YAB) were established, and baseline data were collected across 22 communities (11 C2H communities and 11 control communities). The control group is the 4-H Youth Development Club that operates in 11 counties where there was not corresponding C2H implementation. The idea was to try to map those counties to the extent possible on demographics that related to SV risk. A total of 752 youth has been recruited for the overall sampling, with an aim to enroll about 1,100 participants through the end of the baseline data collection. The sample is diverse and retention rates are high at about 83% in terms of the 6-, 12-, and 18-month follow-ups. For the high school survey data, there are over 2,500 public high school students, which averages about 422 students per school with about an 89% participation rate. For the last 2 years of this work (Part B), the aim is to collect 24-month follow-up data from the same 22 C2H and control communities with the individual-, interpersonal-, and adult-level program participants along with the school-based surveys. Despite back-to-school issues due to the COVID-19 pandemic, the team is confident that they will be able to have six C2H schools and six control schools at the 24-month follow-up with an average of 500 students per school, which will provide data for about 6,000 students. The targeted communities have high rates of non-English speakers, racial and ethnic minorities, sexual and gender minorities, and those with limited access to healthcare. The investigative team is very strong and has extensive experience in areas directly related to the proposed work in their previous studies. This team has forged strong connections and partnerships with communities through the RAB and the YAB. There are many innovations in this application. For instance, a unique aspect of this project is the intent to assess social norms as they relate to reductions in SV according to social networks. Regarding the approach, this application is highly detailed and comprehensive in its plans and builds appropriately on an extensive amount of work that already has been done. A clear and strong theoretical structure underpins the study design and measurement approach. The design is appropriately aligned with the aims of the proposed study and a very strong measurement approach is described. The longitudinal assessment is well-detailed and there is a clear plan to assess risk and

protective factors across the SEM. Well-validated measures will be utilized for direct and indirect SV outcomes. Multiple levels of analysis are planned that include a C2H and control program participant individual-level analysis, an analysis of social networks at the interpersonal level, a school-based community-level analysis youth sampling approach through the California Healthy Kids Survey (CHKS), and a social media-based community-level adult sample that aims to collect geo-coded Twitter data. Potential problems and possible solutions are well-delineated. There is a considerable amount of discussion about the COVID-19 pandemic as it related to the planning for this proposal and the baseline data collection. All Part B activities appear to be feasible, and the timeline seems realistic.

Weaknesses were also identified. Regarding significance, the adult-focused data could have been discussed in more detail in terms of looking at social norms, specifically the Twitter social media platform. There may have been some changes in recent years that could impact the degree to which teens and adults use Twitter. This might result in the data not being as viable as they were in the original application and may impact the potential generalizability to the adult population of interest. Nevertheless, this analysis is innovative as long as Twitter does not implode, and it is a cost-effective way of examining social norms. The narrative indicates that the majority of the intervention sites serve predominantly non-English-speaking communities, and that the communities that have implemented the C2H intervention have prioritized non-English-speaking populations. However, there is no discussion of those communities being included in the evaluation, which seems largely focused on English speakers. The intense community buy-in for the C2H program may impact the potential scalability to other communities, though perhaps this could be accomplished through implementation manuals and other means. Regarding the approach, there are some concerns about the initial equivalence between the C2H intervention group and the youth in the 4-H Program. The 4-H program participants tend to be more homogeneous even if they are from matched counties with the C2H program. It is particularly notable that the youth who are served by 4-H are a different age group (14–19 years of age) from the youth served by C2H (14–24 years of age), which could impact internal validity. This lack of initial equivalence between the experimental and control groups potentially could hamper efforts to isolate any resulting group differences to specific program effects. Insufficient details are provided about the C2H program components, particularly with regard to SV. The plan for evaluating the Twitter data seems fairly static. The team created a data dictionary for Part A that also will be used with the 24-month follow-up. Twitter has very fast-paced, ever-evolving, issue of the moment conversations. This raises doubts that the data dictionary that was developed for the first phase of this work, which seemed to focus on post-election-related content, would be applicable for whatever is occurring when these data are reevaluated. It could have been helpful to have more attention paid to recruitment and retention efforts. For the social network analysis (SNA), there will be follow-up every two weeks. This seems like a very long timeframe in the context of the previous discussion about how people respond within two days. The retention check-ins will occur every six months, which also seems like a long time period to let lapse between connections—especially given how youth interact in these social environments. The team has established a youth connection study on Instagram and is inviting participants to join the RAB, but it is not clear how retention of control group participants would be increased and if that same method is being used. Of concern is that this could result in some design contamination.

Protection of human subjects is acceptable. Inclusion is acceptable in terms of gender, minority groups, and children. Animal welfare, biohazards, and dual use research are not applicable. Overall, this is an outstanding application that is extremely strong with negligible weaknesses.

The budget is recommended as requested. An acceptable resource sharing/data management plan (DMP) is provided.

DESCRIPTION (provided by applicant): Two approaches promoted in the current RFP and the CDC's STOP SV Strategies guidance are 1) Promoting Social Norms that Protect Against Violence, and 2)

Creating Protective Environments. The current proposal to evaluate Close to Home (C2H), a community mobilization SV prevention model, incorporates both approaches within a rigorous and innovative research design. Community mobilization to prevent SV, including the C2H model, is designed to create deep social norms change and enhance protective community environments, yet has not been evaluated. We propose to rigorously evaluate the C2H model via a cluster-matched control trial across 22 diverse communities in California via collection and analyses of social network, social media and community-wide school-based data in order to provide much-needed evidence on the effectiveness of this community-level approach to prevent SV. California is uniquely poised to conduct this research given a) the number of communities implementing C2H, b) CDPH's 13-year commitment to building capacity to support local implementation of this model, and c) CDPH's ongoing partnership on RPE evaluation with University of California San Diego's Center on Gender Equity and Health, a center with the highest levels of expertise in evaluation of sexual violence prevention programs, social network science, and assessing norms across social networks and social media. We propose a highly-innovative, multi-level evaluation of C2H involving the following aims: 1) Develop and implement a cluster-matched control design in partnership with the Research Advisory Board, including collecting baseline social network and social media data across 22 diverse California communities; 2) Evaluate effects of the C2H model on social norms and community connectivity regarding SV, and SV incidence via longitudinal analyses of baseline and 24-month follow-up social network data; and 3) Extend Aim 2 findings via analyses of community-level effects on SV social norms and behavior based on multiple waves of community-level school-based data and geocoded social media data. Through Component A funding, we are well-underway in the rigorous evaluation of the C2H model, with baseline data collection nearly completed, demonstrating the feasibility of the proposed research. The completion of the proposed research during Years 4-5 will fill a critical evidence gap in SV prevention by evaluating effects of C2H, a promising community mobilization SV prevention model, on community-level social norms, community connectivity (a key element of protective environments), and SV incidence in accordance with CDC's STOP SV guidance. Proposed longitudinal social network analyses will also provide the first evidence regarding diffusion of changes in SV social norms and behaviors. These contributions will accelerate prevention of sexual violence by equipping local, state and national stakeholders with the rigorous and generalizable evidence needed to inform implementation of community-level sexual violence prevention programming.

PUBLIC HEALTH RELEVANCE: Community mobilization to prevent SV, including the C2H model, is designed to create deep social norms change and enhance protective community environments, two approaches specified in the current RFP and the CDC's STOP SV Strategies guidance, yet community mobilization for SV prevention has rarely been evaluated. We propose to continue implementing the rigorous evaluation of the C2H model in Years 4-5 via a multi-level cluster-matched control trial across 22 diverse communities in California via collection and analyses of social network, social media and community-level school-based data in order to provide much-needed evidence on the effectiveness of this community-level approach to prevent SV, including reducing harmful social norms and creating protective environments. The proposed highly-innovative research will fill a critical evidence gap in SV prevention by evaluating effects of a promising community mobilization SV prevention model on community-level social norms and incidence of multiple forms of SV (sexual assault, in-person and online sexual harassment, dating sexual violence and coercion, LGBTQ+ sexual violence), in accordance with CDC's STOP SV guidance.

CRITIQUE 1

Significance:	1
Investigator(s):	1
Innovation:	1
Approach:	2
Environment:	1

Overall Impact
Strengths
- The proposed project aims to reduce sexual violence outcomes by targeting three social ecological levels of sexual violence-related social norm influences: community norms, social network/interpersonal norms, and individual attitudes, intentions, and behaviors.
- The applicants describe successful efforts to partner with a community-level and community-driven primary prevention program – the Close to Home (C2H) program – based in underserved and diverse communities that are at high risk for sexual violence perpetration and victimization.
- The applicants describe an impressive set of activities to recruit participants and collect baseline data during Component A in accordance with the multi-level and theory-informed framework that encompasses two key STOP SV strategies outlined in the NOFO, Promoting Social Norms that Protect Against Violence, and Creating Protective Environments.
- The measurement approach and quasi-experimental design of this primary prevention project are well-described, consisting of longitudinal assessment of key SV outcomes combined with innovative large-scale social network analyses. The applicants have been able to retain over 80% of their participants, and the broader project appears well-powered as well as practically feasible.
- The investigative team is particularly strong, with ample experience and expertise in areas directly related to this project.

Weaknesses
- More information could have been provided about how social network data might have changed in terms of viability and generalizability in the years since the initial application.
- The experimental and control groups are from quite disparate populations.

1. Significance:
Strengths
- The applicants provide a comprehensive and compelling overview of Component A activities that established the multilevel evaluative structure to examine the ability of the community based RPE program, Close to Home (C2H), to reduce rates of sexual violence (SV).
- The proposed Component B evaluation is closely aligned with the stated purpose and objectives outlined in the FOA (i.e., Promoting Social Norms and Creating Protective Environments).
- Successful completion of activities outlined for Component B will address a critical gap in the SV prevention literature regarding the effectiveness of a widely adopted and comprehensive community-focused SV prevention model (the C2H program).
- A particular strength of this evaluation project is in its ability to examine violence preventive effects at various layers of the social ecological model, including the outer-layer community level, social network-level interpersonal layer, and attitudinal-focused individual layer.
- The applicants provide a highly detailed and comprehensive evaluative plan that is highly likely to target the specific needs of communities that traditionally have experienced a disproportionate burden of SV, especially as it relates to communities with high rates of non-English speakers, racial/ethnic minorities, sexual/gender minorities, and limited access to health care.

Weaknesses
- No weaknesses are noted.

2. Investigator(s):
Strengths
- The project team, based at the Center on Gender Equity and Health at UCSD, is exceptionally experienced, highly productive, and among the world's leading experts in this area.
- The applicants outline an extensive and successful set of activities undertaken during Component A to build strong working partnerships with the C2H team as well as with related research and assessment partners (e.g., YouGov).

Weaknesses
- No weaknesses are noted.

3. Innovation:
Strengths
- A notable strength of the original Component A plan was the applicants' plan to assess SV risk and protective factors at multiple socioecological levels. The proposal makes clear that the applicants have successfully put this plan into practice in the form of their baseline data collection efforts.
- More specifically, a highly unique aspect of the broader proposal was to examine the diffusion/spread of SV-related social norms across social networks as evidenced by large scale data mapping of social media activity (i.e., Twitter). In the current proposal, the authors clearly outline the practical efforts undertaken in Component A and the planned analyses for Component B that translate this potential innovation into actionable data of relevance to SV prevention.
- The applicants provide a clear theoretical structure to their proposed Component B evaluation, with a focus on the Theory of Planned Behavior and Theory of Bounded Normative Influence.
- Overall, the applicants appear successful in their ability to translate this quasi-experimental design using complex data mapping and statistical analyses into practice, as the baseline recruitment and data collection activities are well aligned with their initial expectations.

Weaknesses
- No weaknesses are noted.

4. Approach:
Strengths
- The overall design of this primary prevention project is well-described and highly detailed across levels of programming (i.e., C2H efforts, network analyses, school-based surveys), participant recruitment, data sources, outcome measures, planned statistical analyses, power considerations, and potential mediators/moderators.
- Potential problems as well as immediate solutions were extensively discussed, especially related to pandemic disruptions to recruitment and data collection. More broadly, the applicants frequently outline potential pitfalls and limitations to their Component A activities and thoughtfully explain their solutions to those problems.
- The sample retention appears to be quite high (83%), and power estimates suggest that the broader investigation is adequately powered.
- The applicants will apply well-validated measures of key direct and indirect SV outcome measures.
- The proposed Component B evaluation activities appear feasible, and the timeline is realistic relative to the proposed data collection and data analyses.

Weaknesses
- The youths in the control group program (4-H) are not initially equivalent to the C2H program youths across a number of demographic and background characteristics. While this could potentially impact efforts to isolate any resulting group differences to specific program effects, the applicants consider this limitation and outline a series of strategies to address these core differences in the two community program samples.
- More could have been discussed about the adult-focused social media platform (Twitter), especially as it relates to any changes within this platform in recent years that might impact data viability or generalizability.

5. Environment:
Strengths

- The scientific environment is very strong, both at the institutional level at UCSD, as well as in field-related environments given the investigators' strong partnerships with the C2H.
- The Research Advisory Board and Youth Research Advisory Board meet frequently and are actively involved in innovative efforts to retain participants in the broader project.
- Given the successful implementation of Component A activities and the establishment of key partnerships essential to recruitment and data collection, it appears that the investigators are well-positioned to complete Component B research plans.

Weaknesses

- No weaknesses are noted.

Protections for Human Subjects:
Acceptable. Human Subjects involved, no concern

Data and Safety Monitoring Plan (Applicable for Clinical Trials Only):
Not Applicable

Inclusion of Women, Minorites, and Age (Applicable Only for Human Subjects research and not IRB Exemption #4.):
G1A- Includes both genders, acceptable
M1A- Includes minorities & non-minorities, acceptable
C6A- Children & adults (no older adults), acceptable

Vertebrate Animals:
Not Applicable

Biohazards:
Not Applicable

Dual Use Research of Concern:
Not Applicable

Budget and Period of Support:
Recommend as Requested

Resource Sharing Plans:
Acceptable

Data Management Plan (DMP):
Acceptable

CRITIQUE 2

Significance:	1
Investigator(s):	1
Innovation:	2
Approach:	3
Environment:	1

Overall Impact

Strengths

- This is a well-designed cluster matched control trial that appears feasible.
- This is a well-written, well-organized proposal and was a pleasure to read.

- This is a timely evaluation of a “highly evaluable” and theoretically solid primary prevention intervention targeting social norms around sexual violence -- with good uptake across racially/ ethnically and geographically diverse communities and with purposive attention to LGBTQ+ youth.
- The investigator team is very strong with very strong partnerships. The excellent use of the research advisory board and youth research advisory board is a strength.
- The novel and innovative component is seen in the longitudinal social network analysis of social norms rejecting SV (and their diffusion over time).

Weaknesses
- The majority of intervention sites serve predominantly non-English speaking communities, and investigators do say that “communities implementing C2H have prioritized non-English speaking populations.” However, there is no discussion of these communities being included in either the intervention itself or the evaluation plan.

1. Significance:
Strengths
- This is a well-timed evaluation in that the C2H program has been in existence for 13 years, with widespread implementation statewide suggesting acceptability among the community.
- This project is theoretically solid and deemed “highly evaluable” by CDC but has not been rigorously evaluated.
- Investigators state “communities implementing C2H have prioritized non-English speaking populations, rural communities, and gender and sexual minority populations who are disproportionately affected by SV.” Working with these populations is a strength.
- There is purposeful inclusion of LGBTQ+ youth in the approach through the YRAB.

Weaknesses
- No weaknesses are noted.

2. Investigator(s):
Strengths
- This is an exceptionally strong team with complementary and relevant expertise and a strong track record of accomplishments and expertise in outcome evaluation.
- The investigators have broad partnerships with 10 rape crisis centers in diverse communities that are already implementing the intervention, and with the 4-H program (control program).
- Dr. White (Co-I) has been with California Department of Health working with RPE for 16 years; CDPH and GEH (PI’s Center) have been in partnership for 7 years.

Weaknesses
- Is there time for Dr. White included in the budget?
- The work plan for Component A is included but not for Component B.

3. Innovation:
Strengths
- This is a theoretically solid program that has shown promise in changing social norms in other public health contexts but does not appear to have been rigorously evaluated for SV.
- The social media analysis component is quite innovative. If effective, this a low cost approach to measuring community level norms around sexual violence.
- Understanding pathways of diffusion of social norm change is also an important contribution.

Weaknesses
- Although there is a clear sense of the evaluation plan for the intervention, the clarity of the intervention itself is not there. It is described in the Appendix but only quite briefly.

4. Approach:
Strengths

- A multi-level cluster matched control trial with 11 treatment sites and 11 control sites is a strong design and appropriate for the proposed aims.
- This project offers a true community-level approach with impact analyzed at multiple levels – individual, interpersonal, community.
- There are multiple measures of SV, including in-person victimization and online, LGBTQ+ sexual violence and coercion. The measures are appropriate and well-chosen.
- The threats to internal validity (e.g., baseline differences of matched groups, retention issues, self-report biases) are reasonably addressed.
- The development of their "SV Twitter dictionary" of Twitter terms related to SV attitudes and behaviors and longitudinal social network analysis of social norms rejecting SV (and their diffusion over time) is novel and fascinating to read. If their approach works this is a valuable contribution in and of itself.
- The timeline appears feasible. The large baseline data set supports the sense that their data collection strategy will succeed.
- Creating an implementation package as part of the approach is another strength.
- The intervention includes a fidelity assessment.

Weaknesses

- There is some confusion in the narrative regarding Twitter data (for the community-level analysis) and who the investigators think this reflects – youth? adults? In some places it seems to say 13+, and in others, it says adults or 18+.
- There is some concern about the stability or durability of Twitter as a platform and any trends regarding this platform.
- The content/approach of intervention itself is unclear which may impact the ability to assess the evaluation plan.
- Eight out of the 11 intervention sites serve predominantly non-English speaking populations. Overall, this seemed a strength initially, but there isn't any information about inclusion of, say, Spanish speaking youth in either the intervention or the evaluation of the intervention, and it is unclear how that will impact the social media analysis.

5. Environment:

Strengths

- This is an exceptionally strong environment with excellent community engagement.
- The project includes a thoughtful use of the research advisory board and youth research advisory board, with specific attention paid to the input of LGBTQ+ youth.

Weaknesses

- No weaknesses are noted.

Protections for Human Subjects:
Acceptable. Human Subjects involved, no concern

Data and Safety Monitoring Plan (Applicable for Clinical Trials Only):
Not Applicable

Inclusion of Women, Minorites, and Age (Applicable Only for Human Subjects research and not IRB Exemption #4.):
G1A- Includes both genders, acceptable
M1A- Includes minorities & non-minorities, acceptable
C6A- Children & adults (no older adults), acceptable
Vertebrate Animals:
Not Applicable

Biohazards:
Not Applicable

Dual Use Research of Concern:
Not Applicable

Budget and Period of Support:
Recommend as Requested

Resource Sharing Plans:
Acceptable

Data Management Plan (DMP):
Acceptable

CRITIQUE 3

Significance:	2
Investigator(s):	2
Innovation:	1
Approach:	3
Environment:	1

Overall Impact
Strengths
- This project offers an innovative approach with multi-level evaluation approach.
- A strength is the inclusion of diverse participants to increase generalizability to other communities.
- A strength is the development of implementation manuals.

Weaknesses
- Because this project consists of extensive community support/buy-in, this may limit adaptation and scalability to other communities.
- The comparison group is demographically different from the intervention group at baseline, which is a concerning threat to internal validity.

1. Significance:
Strengths
- This project focuses on community-level social norms change.
- There are strong community partnerships that make this project highly feasible.
- The inclusion of highly diverse populations (which experience disproportionate burdens of sexual violence) enhances generalizability.

Weaknesses
- Huge amounts of community buy-in and investment limit the scalability of this approach beyond this project/community.

2. Investigator(s):
Strengths
- This is a strong research team with enough experience and expertise to complete the project.
- This team has numerous relevant publications across the study personnel, as well as successful completion of previously funded projects.
- The partnering organizations clearly have the capacity to carry out programmatic goals.
- A strength is the inclusion of a robust research advisory board.

Weaknesses

- Dr. Fowler is listed as the social media expert, but there is a lack of evidence from provided materials that Dr. Fowler has experience analyzing Twitter data specifically.

3. Innovation:
Strengths
- This is the first project to evaluate community mobilization for SV at multiple levels.
- There are no new innovations to methodology, but applying the methods to new areas (community mobilization, social network for SVP, development of LGBTQ+ specific victimization) is an innovation.

Weaknesses
- No weaknesses are noted.

4. Approach:
Strengths
- The approach is an appropriately rigorous design for the project (quasi-experimental design with matched comparison group).
- There is an excellent use of multi-level data to evaluate the impact of the program across individual, interpersonal, and community levels.
- Measures are well described and appear appropriate and reliable.
- There is a clear plan for recruiting/retaining participants informed by the advisory board (including the addition of retention check-ins).

Weaknesses
- This proposal did not include sufficient information to evaluate whether the C2H program focuses on the primary prevention of SV. Although the applicants note that the program addresses two of the three required criteria (promoting social norms and creating protective environments), not enough information on the program components is presented to allow for evaluation of this.
- The selection of 4H-programs as a comparison group creates many threats to internal validity (some of which were acknowledged and some of which were not). As noted from baseline data, the youth who participate in 4H are demographically less diverse than C2H participants. Although applicants identify a statistical workaround for this, it introduces bias, nonetheless. Additionally, from the 4H letter of support, it appears that the target participant in the program are youth ages 14-19, as opposed to the age range of C2H (14-24). Observed differences in program outcomes could be attributable to these different participants, and the applicants have not addressed the potential problem.
- There are several limitations to Twitter data. It is unclear how Twitter data is an appropriate measure of community-level outcomes, as Twitter users skew older than the target audience of C2H (the most common group of Twitter users are 25-34 year olds). Additionally, while the data dictionary has been developed, it appears to be already outdated in its key terms (e.g., dementiajoe). The applicants could plan for an updating/refinement process of the data dictionary.
- The social network surveys pilot yielded lower participations for the second pilot which focused on the LBGTQ+ students/items. The applicants discuss throughout strategies to increase general SNA response rates, but do not specify how these strategies will be tailored to LGBTQ+ youth.
- The applicants describe measures of social capital, but do not describe what is being done with those measures. Because the survey is long (40 minutes), the applicants could eliminate this and other items.
- There is concern that the applicants will not be able to recruit the desired/required number of schools. Additionally, the mean number of students per school right now is 422, but the applicants are anticipating an average of 500 students per school.

- The follow-up time period of 24-months SNA seems long (every two weeks), especially since demonstrated that most youth respond in two days. Additionally, a clear rationale was not provided for why outcomes are being tracked at 24 months (and not any time in between).
- While the inclusion of retention check-ins was a good idea, would more frequency be needed rather than every 6 months? Additionally, the suggestion to connect youth to the YouthConnectStudy on Instagram and inviting youth to join the YRAB is interesting. However might this contribute to contamination for control group participants?

5. Environment:
Strengths
- This is an excellent scientific environment.
- There is a clear description of partnering organizations and roles/responsibilities.
- The application has the necessary letters of support with detailed information about the nature of involvement and support for this rigorous evaluation.

Weaknesses
- No weaknesses are noted.

Protections for Human Subjects:
Acceptable. Human Subjects involved, no concern

Data and Safety Monitoring Plan (Applicable for Clinical Trials Only):
Not Applicable

Inclusion of Women, Minorites, and Age (Applicable Only for Human Subjects research and not IRB Exemption #4.):
G1A- Includes both genders, acceptable
M1A- Includes minorities & non-minorities, acceptable
C6A- Children & adults (no older adults), acceptable

Vertebrate Animals:
Not Applicable

Biohazards:
Not Applicable

Dual Use Research of Concern:
Not Applicable

Budget and Period of Support:
Recommend as Requested

Resource Sharing Plans:
Acceptable

Data Management Plan (DMP):
Acceptable

THE FOLLOWING SECTIONS WERE PREPARED BY THE SCIENTIFIC REVIEW OFFICER TO SUMMARIZE THE OUTCOME OF DISCUSSIONS OF THE REVIEW COMMITTEE, OR REVIEWERS' WRITTEN CRITIQUES, ON THE FOLLOWING ISSUES:

PROTECTION OF HUMAN SUBJECTS: ACCEPTABLE

INCLUSION OF WOMEN PLAN: ACCEPTABLE
Includes both women and men, which is scientifically acceptable.

INCLUSION OF MINORITIES PLAN: ACCEPTABLE
Includes both minorities and non-minorities, which is scientifically acceptable.

INCLUSION ACROSS THE LIFESPAN: ACCEPTABLE
Includes children and adults, but no older adults, which is scientifically acceptable.

COMMITTEE BUDGET RECOMMENDATIONS: The budget was recommended as requested.

COMMITTEE RESOURCE SHARING PLAN RECOMMENDATIONS:
The Resource Sharing Plan is acceptable.

COMMITTEE DATA MANAGEMENT PLAN RECOMMENDATIONS:
The Data Management Plan is acceptable.

MEETING ROSTER

National Center for Injury Prevention Special Emphasis Panel
NATIONAL CENTER FOR INJURY PREVENTION AND CONTROL
CE20-001: Evaluating Practice-based Programs, Policies, and Practices from CDCs Rape Prevention and Education (RPE) Program: Expanding the Evidence to Prevent Sexual Violence
ZCE1 TQD (01)
02/07/2023

CHAIRPERSON(S)

COKER, ANN L, PHD, MPH, BS
PROFESSOR
UNIVERSITY OF KENTUCKY
COLLEGE OF MEDICINE
LEXINGTON, KY 40536

MEMBERS

BLOOM, TINA L, PHD
ASSOCIATE PROFESSOR AND CHAIR
NOTRE DAME OF MARYLAND UNIVERSITY
SCHOOL OF NURSING
BALTIMORE, MD 65211

ECKHARDT, CHRISTOPHER I, PHD
PROFESSOR & DIRECTOR OF CLINICAL TRAINING
PURDUE UNIVERSITY
DEPARTMENT OF PSYCHOLOGICAL SCIENCES
WEST LAFAYETTE, IN 47907

GREEN, JULII MONETTE, BS, MS, PHD
ASSISTANT PROFESSOR
ALLIANT INTERNATIONAL UNIVERSITY
LA MESA, CA 91941

MENNICKE, ANNELISE, BS, MSW, PHD
UNIVERSITY OF NORTH CAROLINA, CHARLOTTE
CHARLOTTE, NC 28223

SABINA, CHIARA, PHD, MA, BA
RUTGERS UNIVERSITY
NEW BRUNSWICK, NJ 08301

SCIENTIFIC REVIEW OFFICER

GENTLES, CARLISHA, PHMD
SCIENTIFIC REVIEW OFFICIAL
NATIONAL CENTER FOR INJURY PREVENTION & CONTROL
NATIONAL CENTER FOR ENVIRONMENTAL HEALTH/ATSDR
EXTRAMURAL RESEARCH PROGRAM OPERATIONS
CENTERS FOR DISEASE CONTROL & PREVENTION
ATLANTA, GA 30341

EXTRAMURAL SUPPORT ASSISTANT

ROBERTS, KENNETH H
GRANTS TECHNICAL ASSISTANT
NATIONAL CENTER FOR INJURY PREVENTION & CONTROL
NATIONAL CENTER FOR ENVIRONMENTAL HEALTH/ATSDR
EXTRAMURAL RESEARCH PROGRAM OFFICE
CENTERS FOR DISEASE CONTROL & PREVENTION
ATLANTA, GA 30341

Consultants are required to absent themselves from the room during the review of any application if their presence would constitute or appear to constitute a conflict of interest.

Peer Review Summary Statement Response

PI: Jay Silverman

Project: *Evaluation of the Close to Home Program in California. Assessing the Impact of Community Mobilization to Prevent Sexual Violence at the Individual, Social Network, and Community Levels.*

We are grateful for the feedback on our Component B proposal: *Evaluation of the Close to Home Program in California. Assessing the Impact of Community Mobilization to Prevent Sexual Violence at the Individual, Social Network, and Community Levels.* We write to address the identified application weaknesses highlighted by the review committee and shared with our team upon notice of award. The response is organized broadly into the following themes: *Study Design*, *Intervention Design, Data Sources, and Budget and Key Personnel*. We have bolded text of reviewers' comments and used non-bolded text for our response. We appreciate the opportunity to respond to this feedback.

1. Study Design

1.1. Control group

Reviewer 1: The experimental and control groups are from quite disparate populations...The youths in the control group program (4-H) are not initially equivalent to the C2H program youths across a number of demographic and background characteristics. While this could potentially impact efforts to isolate any resulting group differences to specific program effects, the applicants consider this limitation and outline a series of strategies to address these core differences in the two community program samples.

Reviewer 3: The selection of 4H-programs as a comparison group creates many threats to internal validity (some of which were acknowledged and some of which were not). As noted from baseline data, the youth who participate in 4H are demographically less diverse than C2H participants. Although applicants identify a statistical workaround for this, it introduces bias, nonetheless. Additionally, from the 4H letter of support, it appears that the target participant in the program are youth ages 14-19, as opposed to the age range of C2H (14-24). Observed differences in program outcomes could be attributable to these different participants, and the applicants have not addressed the potential problem.

The California 4-H Youth and Development Program was selected as the control group for this study because it is widely present across California (nearly in every county), engages youth in prosocial learning projects unrelated to sexual/dating violence prevention (e.g., agriculture, computer science, art), and employs an adult-youth partnership model similar to the Close to Home (C2H) youth-adult model. Reviewers point out that the demonstrated difference in racial/ethnic and age demographic between C2H groups and their matched 4-H groups demeans the equivalency of the intervention and control group. Indeed, despite our best efforts to identify a willing, widely available, and similarly structured control group that would draw similar types of youth, we have found significant differences between these groups. As noted in the Component B proposal, however, we found there to be no significant differences across C2H and 4-H members (i.e., egos) in sexual violence outcomes, which is a critically important finding regarding equivalency for assessing effects. Because this is a quasi-experimental design that is likely to have limitations to "exchangeability" between intervention and control groups, we have used and will use a variety of strategies to mitigate these demographic baseline differences:

1) The research team recruited propensity score matched 4-H clubs in communities with larger Latino and urban populations to offset intervention and control sample demographic differences following the submission of the Component B proposal.
2) Though the purported age range for 4-H is up to age 18, in practice we found young people ages 18-24 still involved with the 4-H groups. Still, age differences between the groups were observed; the average age of control participants is 16.5 years (range 14-24 years, standard deviation (SD): 2.2) and that of intervention participants is 17.1 (range 14-24, SD: 2.3), which are significantly different. To limit the impact of this difference across groups, we may limit the intervention sample to the equivalent age range that is available in the control sample, making ages more equivalent across groups. Because there were not a large number of 18-24 years olds in the ego intervention sample either, we do not expect the need to drop many people from the sample. Both groups were allowed to nominate peers in the full eligible age range (14-24 years old).
3) We will minimize the impact of any baseline differences using inverse probability of treatment weighting to re-weight data to minimize differences across groups at baseline coupled with a difference-in-differences estimator for primary outcome analyses to look at the relative difference from baseline within a group, without assuming equivalence at baseline.
4) If more severe differences are observed across groups, we may alternatively employ a cluster-based individual-level propensity score matching techniques (i.e., employ propensity score matching based on key demographics and outcomes within each community sample with those individuals in the matched community) to preserve the community-level matching but increase the equivalency of the individuals. We would limit the sample to those for whom reasonable matches were available.
5) These strategies may limit the statistical power of our analyses, so may impact our ability to see differences between groups at follow-up on rarely reported outcomes, like perpetration. This may become a limitation of our evaluation but we are hopeful that the sample size will remain close to 900 even with these unmatched participants dropped from the sample.

<u>1.2 Follow-up data collection</u>

Timing of Follow-up:

Reviewer 3: A clear rationale was not provided for why outcomes are being tracked at 24 months (and not any time in between).

Follow-up social network (SNA) data collection is being implemented at 24-months to allow C2H groups to move through all four phases of the C2H model, which typically takes about 1-2 years. Data collection for this evaluation aligns with the implementation period of C2H, with baseline data collection occurring at the start of C2H implementation specific to each participating C2H site (either Fall/Winter 2021-22 or 2022-23) and 24-month follow-up in Fall/Winter 2023-24 or 2024-25. The four phases of C2H can vary in duration, with most organizations moving through the model over 1 to 2 years. Given the longest implementation period being about 2 years and that this is a long-term, deep intervention approach that we would expect to have lasting effects for at least 6-12 months, we chose a 24-month follow-up period. The effort of collecting data and the burden on participants to do more frequent follow-up was determined to be costly to our retention to 24 months.

Reviewer 3: The follow-up time period of 24-months SNA seems long (every two weeks), especially since demonstrated that most youth respond in two days.

It was proposed that baseline SNA participants be sent a message to inform them that they will receive the follow-up survey link one week before the survey link is sent out. Following the circulation of the survey link, participants would receive follow-up reminders to take the survey every 2 weeks. This interval was selected because another important learning from our baseline data collection was that participants perceived our more frequent reminders to take the survey as being "spam" and a nuisance (anecdotal from YRAB members and egos). We experienced improved response rates when we spaced our reminders more, even though we found that if they were going to respond to a reminder, it would happen within 2-3 days. Reviewers point out that the proposed 2 week interval seems too long based on the learning from baseline data collection that most survey responses occur within 2-3 days of the survey link being sent and after that period participation rates drop substantially. In response to this feedback, the new plan is as follows: Follow-up participants will be texted with reminders to participate once a week at various days/times for one month. If nothing is heard back via text, invited participants will also be sent email reminders following the same frequency structure. Participants who did not provide a phone number at baseline will receive two months of weekly email reminders at various days/times during the week. If the invited participant does not respond during this initial reminder period, the research team will send an additional survey invitation once a month within one year of the initial follow-up invitation.

1.3 Recruitment

Reviewer 3: The social network surveys pilot yielded lower participations for the second pilot which focused on the LBGTQ+ students/items. The applicants discuss throughout strategies to increase general SNA response rates, but do not specify how these strategies will be tailored to LGBTQ+ youth.

Reviewers highlighted that while increasing SNA response rates in general was addressed by the research team, we did not discuss if and how we were tailoring these efforts to LGBTQ+ youth. Efforts to increase response rates were focused on removing barriers for all youth to nominate their friends to participate and were not tailored to LGBTQ+ youth because low response rate was a universal issue. The baseline SNA sample has a large LGBTQ+ representation (43% LGBTQ+ youth; n=294), so the research team had no challenges recruiting an LGBTQ+ sample and will be able to carry out analyses for this population, including comparisons between LGBTQ+ and non-LGBTQ+ youth. Because the SNA survey included expansive response options for sexual and gender identity, we are also able to compare experiences of sexual and dating violence between multiple sub-categories of gender and sexual identities. In future work, we note that tailoring recruitment efforts to LGBTQ+ youth is of continued importance in ensuring that the sample size is large enough to run analyses, particularly for transgender and non-binary individuals.

Reviewer 3: There is concern that the applicants will not be able to recruit the desired/required number of schools. Additionally, the mean number of students per school right now is 422, but the applicants are anticipating an average of 500 students per school.

Another concern highlighted by reviewers was the research team's ability to recruit the required number of schools to participate in the California Healthy Kids Survey custom module on sexual and dating violence. Due to immense burden on schools and school administrators in the aftermath of the COVID-19 pandemic and the nature of the survey topic, the research team faced difficulty in recruiting all the schools in intervention and control communities. Despite great effort on the part of the research team and increased recruitment incentives to schools (up to $2000/school), the final baseline CHKS sample included 4 intervention community high schools and 2 control community high schools. Schools that participated at baseline will be recruited for follow-up data collection during either the 2023/24 or 2024/25 academic

school years. In addition to the 6 high schools that were recruited to participate, a large school district in California agreed to administer the module to all their 9th/11th grade students for two academic school years. A representative CHKS sample (N=8,436) of youth, 29% identifying as LGBTQ+ was gathered from this school district. This large sample size has allowed the research team to examine the validity of the novel LGBTQ+ sexual/dating violence measure, as well as the expansive sexual and gender identity items that were included in the SNA and CHKS surveys as part of supplemental funding for Component A.

An alternative strategy we may explore to account for the very low participation of control community schools could be to use the data from the large school district to serve as an alternative source of control data. Using propensity score matching at the individual level (based on demographics and risk behaviors) and propensity score matching between intervention community school students (n=6) and schools in the large school district that match best (based on school and community demographics) with those intervention community schools, we could identify best matches at the individual and school level at baseline to serve as controls. Data were collected 2021/22, 2022/23, and are planned for 2023/24 and 2024/25 at this school district, such that we have the opportunity to use this school districts data at corresponding baseline and follow-up time points for each of the four intervention schools that participated. While this was not the originally intended match, it could serve as a helpful proxy control given the limited participation of control community schools.

1.4. Retention

Retention check-ins

Reviewer 3: While the inclusion of retention check-ins was a good idea, would more frequency be needed rather than every 6 months?

Retention check-ins were originally proposed to be carried out every six months. Retention check-ins are actually carried out roughly every two weeks because it takes multiple attempts to confirm contact information with youth participants. If participants respond after one attempt, they are not re-contacted for 6 months. This is very rare. On average, it takes study personnel four attempts to confirm contact information with participants. Study personnel attempt to reach out via text first, and then email if the first strategy does not work. This equates to 2-3 months of semi-regularly reaching out to participants via multiple avenues of communication in attempts to confirm contact information for follow-up data collection. This method of rolling retention has shown to be effective, with an 83% retention rate of baseline participants.

Study Instagram

Reviewer 3: Additionally, the suggestion to connect youth to the YouthConnectStudy on Instagram and inviting youth to join the YRAB is interesting. However might this contribute to contamination for control group participants?

A study Instagram account was created following suggestions from the Youth Research Advisory Board (YRAB) that having an Instagram could help to legitimize the study in the eyes of youth participants during recruitment of baseline participants and retention efforts, particularly social network nominated participants who had no direct contact with the study team. This suggestion was acted upon in tandem with evidence from other studies that show 1) that engaging youth via Instagram is a viable strategy for increasing youth participation,[1] and 2) that involving youth in the design of retention strategies improves

retention.[2] In the future, the study Instagram page will also serve as a platform for grassroots dissemination of baseline and follow-up study findings to youth across California. The creation of the Instagram page (@youthconnectstudy) coincided with a re-branding of the study name to make it more accessible and recognizable to youth (from "Close to Home Evaluation" to "Youth Connect Study"). The Instagram handle was added to survey communications and survey participants were encouraged to follow the page (both intervention and control). During orientations the research coordinator would share the Instagram handle and encourage people to follow it for updates on the study. The Instagram page was also advertised to study participants during retention check-ins. The YRAB curated the Instagram page during social network recruitment, deciding what to post and creating posts and stories under the supervision of the research team. Posts broadly summarized the study goals, recruited YRAB participants, highlighted current YRAB members, highlighted Close to Home community activities (such as a Dia de los Muertos celebration), introduced the 4-H Club, shared stories about community data collection in both C2H and 4-H communities, and discussed adjacent but programmatically unrelated content, such as Title IX (topic selected by YRAB). Detailed C2H or 4-H programmatic content was not shared to avoid contaminating the sample. Sample contamination is not a concern for this reason, and because the research team believes a small number of potential study participants follow the page. Currently, the Youth Connect Study Instagram has 110 followers, of which 25% are organizations, not individuals.

1.5. Inclusion of Monolingual Spanish Speakers in Evaluation

Reviewer 2: The majority of intervention sites serve predominantly non-English speaking communities, and investigators do say that "communities implementing C2H have prioritized non-English speaking populations." However, there is no discussion of these communities being included in either the intervention itself or the evaluation plan....Eight out of the 11 intervention sites serve predominantly non-English speaking populations. Overall, this seemed a strength initially, but there isn't any information about inclusion of, say, Spanish speaking youth in either the intervention or the evaluation of the intervention, and it is unclear how that will impact the social media analysis.

It is true that the C2H program sites have prioritized non-English-speaking populations. Many of the C2H programs have monolingual adult C2H groups that are conducted entirely in Spanish. We found, however, that even in these same communities, the youth C2H groups, although predominately Latino and many from monolingual households, were comprised of youth who felt comfortable reading and writing in English, primarily because of their exposure to English in school. Despite C2H programming being available in English and Spanish (with bilingual facilitators) at these sites with predominantly non-English-speaking adults, youth groups were run in either English or a mix of English and Spanish because facilitators found the youth to be fluent in both languages. Reflecting this, prior to each data collection site visit, the research team communicated with the site facilitator privately to understand the needs around Spanish-translation of the study material, including the assent/consent forms, the survey orientation, and the survey, for their youth members. This need for Spanish translation was only identified twice throughout the duration of baseline data collection. In the first case, the potential participants decided to not move forward with the C2H program for unknown reasons (all C2H programming was available in English and Spanish at this site). The second time the research team was aware of the need for translation, we made translated versions of the assent/consent form and the survey available and offered the survey orientation in Spanish and in English, giving participants a choice for which orientation they joined so as not to single anyone out. The Spanish version of the survey was verified and approved by UC San Diego IRB. From that point forward, all participants (including nominated alters) could select to take the survey in Spanish or English. While the

sample does not, therefore, represent a large proportion of monolingual Spanish-speakers, it likely represents a large sample of Spanish-speakers who are bilingual.

For engagement with the RAB and dissemination, one of the adult C2H coordinators is a monolingual Spanish speaker and a part of the Research Advisory Board (RAB). Since this individual joined the RAB, the research team has worked to send emails in English and Spanish, to provide translated PowerPoint slides prior to meetings, and to provide live translation during the meeting so this individual can fully participate. A communications consultant with expertise in grassroots communications campaigns will be hired to work with the research team to design an inclusive and community-based dissemination of the study results. All dissemination materials will be translated into Spanish. Similar to how C2H implements community conversations, the research team will work with community- based advocates to hold bilingual events to share the results of the evaluation.

For the social media analysis, see section titled, "Twitter Data Analysis”.

1.6. Measurement

Reviewer 3: The applicants describe measures of social capital, but do not describe what is being done with those measures. Because the survey is long (40 minutes), the applicants could eliminate this and other items.

One reviewer noted that we should consider removing measures of social capital to shorten the survey. There are only two items in the SNA survey that fall under the social capital construct. One is about community connectedness and the other about social cohesion. It would have been clearer if we had used the latter construct names instead of “social capital” the importance of these items. Because increasing community connectedness and social cohesion are outcomes of the C2H model, the research team will keep these items in the survey. We will use these secondary outcomes in all evaluation analyses.

1.7. Social network data

Reviewer 1: More information could have been provided about how social network data might have changed in terms of viability and generalizability in the years since the initial application.

The social network sample, though much smaller than originally proposed, is viable and strong for the intended evaluative purposes. While collection of the social network data turned out to be extremely difficult and slow, we were able to recruit a sample of 1000 youth from 22 communities across California. On average, program youth successfully recruited 1.61 of their peers to participate in the study. As we included in the Component B proposal, the social network dyads show signs at baseline of correlation, indicating that youth and their peers are likely to exhibit similar attitudes and behaviors, a positive validity check on the social network sample. While social network sampling is not intended to provide a representative sample, the youth in the sample are from urban, suburban, and rural regions and are racial/ethnic diverse. Because selection for the intervention was pre-determined and not random, a random sample for this evaluation is not appropriate.

1.8. Twitter Data Analysis

Reviewer 1: More could have been discussed about the adult-focused social media platform (Twitter), especially as it relates to any changes within this platform in recent years that might impact data viability or generalizability.

Reviewer 2: There is some confusion in the narrative regarding Twitter data (for the community-level analysis) and who the investigators think this reflects – youth? adults? In some places it seems to say 13+, and in others, it says adults or 18+.

Reviewer 3: There are several limitations to Twitter data. It is unclear how Twitter data is an appropriate measure of community-level outcomes, as Twitter users skew older than the target audience of C2H (the most common group of Twitter users are 25-34 year olds). Additionally, while the data dictionary has been developed, it appears to be already outdated in its key terms (e.g., dementiajoe). The applicants could plan for an updating/refinement process of the data dictionary.

After much consideration of the known challenges with the social media data source for the evaluation and after monitoring the rapid and dramatic changes to Twitter (now called "X") since the original Component A proposal, the research team has decided to not move forward with the Twitter follow-up data collection and analysis. This decision was reached based on the comments from reviewers regarding limitations to this analysis and recent data access barriers introduced as a result of the switch in ownership of the Twitter platform. The original proposal was to use machine learning to create a tool that could be used to link "tweets" or language posted on Twitter with attitudes and perceived norms around sexual violence and that this tool would be applied to geocoded tweets from the intervention and control communities. Because at the time of proposal, primarily adults (vs. Adolescents) were Twitter users, this was a strategy to assess change in the online normative space among adults in the study communities. he tool was created using survey data collected from a YouGov panel of adults (N=1,000) who were willing to provide their Twitter handle/access to their tweets. Questions in this survey related to attitudes, behaviors, and social norms related to sexual violence. Machine learning models allowed us to understand the differences in patterns of speech (using respondent's original tweets) between those with positive attitudes and behaviors related to sexual violence vs. those holding negative attitudes and behaviors. These speech and content patterns constituted the tool/model that could then be used to predict the status of attitudes and behaviors in specific geographies (i.e., the intervention and control sites) based on the tweets generated from those areas. The key rationale behind this exercise was that tweets posted by users would reflect their attitudes and behavior to a large extent.

Limitations

This was an innovative, risky component of our original proposal, even had the conditions of the Twitter environment stayed similar across the study period. Now, given all that has changed in the way Twitter is managed and the impact that has had on use of the platform and its user profile, the utility of this analysis seems weak. A key limitation of the proposed Twitter/"X" data analyses was that the generalizability of findings from this tool would be limited due to the difference in demographics of the sample of "X" users who participated in the YouGov survey and the demographics of intervention communities. Another key limitation was that the predictive tool was built based on tweets posted in 2021 and 2022. As reviewers pointed out, "X" conversations are typically reflective of current affairs and events. As such, the identified word patterns that formed the basis of the predictive tool were only to a specific time-period. The developed tool is therefore only valid for predictions of "X" user attitudes, beliefs, and behaviors from timeframes closer to 2021 and 2022. For example, many predictive phrases were related to 2020 election politics in the United States, such as "dementiajoe" and "libshit". To address this limitation, the research team could work with YouGov and the UCSD IRB to get permission to re-access the Twitter handles of the original sample of survey respondents to: 1) carry out another API pull of recent tweets, and 2) use these

tweets to build a second predictive tool for follow-up data collection, rather than using the same tool created from tweets collected during baseline as was originally proposed. This would allow for a more accurate proxy measure of the geographic area attitudes and norms at two timepoints, pre- and post-intervention. *However, the effort and costs of doing this approach that would be necessary for the validity of results seems to not warrant the end product, given the major changes in "Twitter" users and use of the social media platform that would obscure changes due to the intervention.*

Instability of Data Source

Elon Musk acquired Twitter in October of 2022 and changed the name to "X" in July of 2023. The leadership of Musk has brought significant changes in who is using Twitter ,[3] how tweets can be viewed,[4] and how tweets are moderated.[5] The Pew Research Center reported in July of 2023 that by Spring of 2023, 60% of U.S. adults who had used Twitter in the past year said that they'd taken a break from the platform in the past 12 months.[6] Women were more likely than men to say they had taken a break, and Black users were more likely than White users to say the same. Pew also found that following Musk's acquisition of the site, most highly active users continued to use the platform, but posted less frequently. Important to our proposed analyses that would rely on original tweets, the majority (three-quarters) of tweets between October 2022 and April 2023 were re-tweets or replies, rather than original tweets.[6] In July of 2023, "X" rolled out new restrictions on viewing tweets, including restricting viewing tweets to those who have "X" accounts (previously anyone could view), and restricting the number of tweets that could be viewed in a day for unverified accounts (originally to 600 per day and later changed to 1,000 per day).[4] There have also been concerns about the platform's ability to safely monitor tweet content following the mass laying off employees and a surge in hate speech on the platform. In December of 2022, Reuters reported that Musk was utilizing automation to moderate content and had mostly done away with manual review of content.[5]

In addition to concerns about the shifting nature of the "X" platform, the research team was concerned that access to the Twitter API v2 could be restricted following the change in ownership. This prompted the research team to download, and back-up all baseline geo-coded tweet data needed for building the tool in November of 2022. As feared, a new three-tier pricing system for API access was unveiled in March of 2023, including a free account (no tweet pulls allowed), a Basic account ($100/month, 10,000 tweet pull cap), and a Pro account ($5,000/month, 1,000,000 tweet pull cap). This change constitutes two major barriers to the research team in moving forward: the cost of the Pro account, and the cap of 1,000,000 tweets (the first tool created used 1,508,643 tweets).

While the creation of this tool served as an important opportunity to explore a novel and cost-effective method for proxy measurement of community-level attitudes and norms relating to sexual and dating violence, the instability of the "X" platform and the existing limitations make moving forward with follow-up data collection hard to justify.

2. Intervention Design

2.1. C2H Model

Reviewer 2: Although there is a clear sense of the evaluation plan for the intervention, the clarity of the intervention itself is not there. It is described in the Appendix but only quite briefly... The content/approach of intervention itself is unclear which may impact the ability to assess the evaluation plan.

Reviewer 3: This proposal did not include sufficient information to evaluate whether the C2H program focuses on the primary prevention of SV. Although the applicants note that the program addresses two of the three required criteria (promoting social norms and creating protective environments), not enough information on the program components is presented to allow for evaluation of this.

Due to space limitations and given what was already included in the Component A application, we chose to limit the description of the C2H program because it had already been reviewed positively as a promising SV primary prevention program.

C2H is a community mobilization approach to preventing sexual and dating violence at individual and community levels. Participants in Close to Home participate in a program orientation focused on providing a foundation in knowledge about the issue of sexual and dating violence. The orientation is broken into the following eight sections: 1) the roots of violence; 2) teen dating violence; 3) sexual violence; 4) healthy relationship skills; 5) media/tech/gender; 6) upstander intervention to prevent sexual and dating violence; 7) community connection; and 8) leadership skills. Once youth and adult participants are oriented to the issue of sexual and dating violence, they commence the C2H phase model to learn about how sexual and dating violence impacts their communities, share that knowledge with the community, and then act to shift social norms to prevent violence. The first phase is *Assess*, in which the community of focus is identified, learning questions around the impacts of sexual and dating violence and related risk and protective factors are developed, community mapping is undertaken, community surveys are developed and administered, and the results of the survey are shared with the community via group events or 1-1 conversations. During the *Talk* phase, participants conduct kitchen table talks, art and storytelling activities, and community conversations in public meeting spaces to dialogue about sexual and dating violence prevention in the community. Then, during the *Build* phase, C2H participants divide into project teams, develop ideas for campaigns and actions related to sexual and dating violence prevention, and foster team skills. In the final *Act* phase, C2H participants implement campaigns and actions related to sexual and dating violence prevention and hold community building events to recruit community-members to join in on the actions. While the program is oriented to preventing sexual and dating violence, sometimes this looks like addressing related risk and protective factors related to community safety broadly (e.g., gang violence prevention, safe space creation) and other social determinants of health. While the community-led aspects of C2H make it challenging to standardize and evaluate, we believe there is sufficient structure to the C2H program to reasonably expect impact on SV outcomes, as well as social norms and protective environments.

2.2. Model Scalability

Reviewer 3: Because this project consists of extensive community support/buy-in, this may limit adaptation and scalability to other communities...Huge amounts of community buy-in and investment limit the scalability of this approach beyond this project/community.

We believe that C2H is highly scalable across a wide variety of communities in California, as has been done for 13 years in California with CDPH support. One of the strengths of the C2H program is the flexibility of the model. While it does require community buy-in, the flexibility allows for this intervention to be tailored to each community it is implemented in to ensure that buy-in and acceptability is feasible. The C2H model meets the community where it is and focuses on creating and mobilizing community-based agents of social change to shift social norms for sustainable community violence prevention. In addition to the nature of the model, the California Department of Public Health has funded C2H implementation in California for 13 years and has demonstrated its widespread acceptability and feasibility across a diverse group of

communities. Process data from our evaluation will be reviewed to continue to learn about the scalability of this model in a post-COVID landscape. These data will allow us to assess costing of the program, necessary staff skills and resources, necessary community characteristics for successful implementation and key community characteristics that are barriers to implementation. Of the 11 sites funded to implement C2H included in this evaluation, all were successful in implementing the model. Their experiences offer a wealth of information about what resources and community characteristics are needed to facilitate implementation.

As proposed, we will use this process data and evaluation outcome data from subgroup analyses with high fidelity programs to understand essential elements for implementation and to develop manuals and support materials for implementation. These materials will build upon the many that are already available through CDPH and ValorUS's TA support for the C2H model over the past 13 years.

2.3. C2H Inclusion of Monolingual Spanish Speakers

Reviewer 2: The majority of intervention sites serve predominantly non-English speaking communities, and investigators do say that "communities implementing C2H have prioritized non-English speaking populations." However, there is no discussion of these communities being included in either the intervention itself or the evaluation plan. Eight out of the 11 intervention sites serve predominantly non-English speaking populations. Overall, this seemed a strength initially, but there isn't any information about inclusion of, say, Spanish speaking youth in either the intervention or the evaluation of the intervention, and it is unclear how that will impact the social media analysis.

Spanish-speaking communities are the community of focus for eight of the eleven C2H groups participating in the evaluation. These communities are often comprised of adults who primarily or only speak Spanish, and their children, who are most often bilingual, having grown up and attended English-speaking school in the U.S. Several of the adult C2H groups are primarily conducted in Spanish and intergenerational group meetings are often conducted in Spanish and in English. Many C2H actions are tailored to the Latinx community, such as Dia de los Muertos vigils that honor women in the community who experienced lethal intimate partner violence.

3. Budget, Key Personnel, and Workplan

3.1. Budget

Reviewer 2: Is there time for Dr. White included in the budget?

Mina White, a Co-investigator on this evaluation, was not included in the budget because she is a employed full time by the California Department of Public Health and cannot accept payment because her role is funded by a CDC RPE grant.

3.2. Study personnel expertise

Reviewer 3: Dr. Fowler is listed as the social media expert, but there is a lack of evidence from provided materials that Dr. Fowler has experience analyzing Twitter data specifically.

Dr. Fowler has been involved in many Twitter data analyses and has been the source of Twitter data for our team:

Christakis, N. A., & Fowler, J. H. (2013). Social contagion theory: examining dynamic social networks and human behavior. *Statistics in medicine*, *32*(4), 556-577.

Garcia-Herranz, M., Moro, E., Cebrian, M., Christakis, N. A., & Fowler, J. H. (2014). Using friends as sensors to detect global-scale contagious outbreaks. *PloS one*, *9*(4), e92413.

Kryvasheyeu, Y., Chen, H., Obradovich, N., Moro, E., Van Hentenryck, P., Fowler, J., & Cebrian, M. (2015). Nowcasting disaster damage. *arXiv preprint arXiv:1504.06827*.

Steinert-Threlkeld, Z. C., Mocanu, D., Vespignani, A., & Fowler, J. (2015). Online social networks and offline protest. *EPJ Data Science*, *4*(1), 1-9.

Kryvasheyeu, Y., Chen, H., Obradovich, N., Moro, E., Van Hentenryck, P., Fowler, J., & Cebrian, M. (2016). Rapid assessment of disaster damage using social media activity. *Science advances*, *2*(3), e1500779.

Settle, J. E., Bond, R. M., Coviello, L., Fariss, C. J., Fowler, J. H., & Jones, J. J. (2016). From posting to voting: The effects of political competition on online political engagement. *Political Science Research and Methods*, *4*(2), 361-378.

Baylis, P., Obradovich, N., Kryvasheyeu, Y., Chen, H., Coviello, L., Moro, E., ... & Fowler, J. H. (2018). Weather impacts expressed sentiment. *PloS one*, *13*(4), e0195750.

3.3. Workplan

Reviewer 2: The work plan for Component A is included but not for Component B.

The RPE workplan included in both of our Component A and B proposals is the workplan for CDPH's RPE program that corresponds to the RPE program that is being evaluated. We understood that there was no need to update this from our Comp A proposal.

References

1. Thomas VL, Chavez M, Browne EN, Minnis AM. Instagram as a tool for study engagement and community building among adolescents: A social media pilot study. *Digital Health.* 2020;6:2055207620904548.
2. Taylor RM, Aslam N, Lea S, Whelan JS, Fern LA. Optimizing a retention strategy with young people for BRIGHTLIGHT, a longitudinal cohort study examining the value of specialist cancer care for young people. *Journal of adolescent and young adult oncology.* 2017;6(3):459-469.
3. Dang S. Exclusive: Twitter is losing its most active users, internal documents show. *Reuters*2022.
4. Taylor J, Milano D. How Twitter's new drastic changes will affect what users can view on the site. *Guardian.* 7/3/2023, 2023.
5. Paul K, Dang S. Exclusive: Twitter leans on automation to moderate content as harmful speech surges. *Reuters.* 12/5/2022, 2022.
6. Dinesh S, Odabas M. *8 facts about Americans and Twitter as it rebrands to X.* Pew Research Center; 7/26/2023 2023.
